# Primo: integration of multiple GWAS and omics QTL summary statistics for elucidation of molecular mechanisms of trait-associated SNPs and detection of pleiotropy in complex traits

**DOI:** 10.1186/s13059-020-02125-w

**Published:** 2020-09-11

**Authors:** Kevin J. Gleason, Fan Yang, Brandon L. Pierce, Xin He, Lin S. Chen

**Affiliations:** 1 Department of Public Health Sciences, University of Chicago, 5841 South Maryland Ave MC2000, Chicago, 60637 IL USA; 2 Department of Biostatistics and Informatics, Colorado School of Public Health, University of Colorado Anschutz Medical Campus, 13001 E. 17th Place, Aurora, 80045 CO USA; 3 Department of Human Genetics, University of Chicago, 920 E 58th St, Chicago, 60637 IL USA

**Keywords:** Integrative genomics, Multi-omics, GWAS, Omics QTL, Molecular mechanisms, Conditional association analysis, Pleiotropy

## Abstract

To provide a comprehensive mechanistic interpretation of how known trait-associated SNPs affect complex traits, we propose a method, Primo, for integrative analysis of GWAS summary statistics with multiple sets of omics QTL summary statistics from different cellular conditions or studies. Primo examines association patterns of SNPs to complex and omics traits. In gene regions harboring known susceptibility loci, Primo performs conditional association analysis to account for linkage disequilibrium. Primo allows for unknown study heterogeneity and sample correlations. We show two applications using Primo to examine the molecular mechanisms of known susceptibility loci and to detect and interpret pleiotropic effects.

## Background

In the post-genomic era, genome-wide association studies (GWAS) have identified tens of thousands of unique associations between single nucleotide polymorphisms (SNPs) and human complex traits [1, 2]. Most of the trait-associated SNPs have small effect sizes and many reside in non-coding regions [3, 4], obscuring their functional connections to complex traits. It is known that trait-associated SNPs are more likely to also be expression quantitative trait loci (eQTLs) [5]; thus, many of these SNPs likely affect complex traits through their effects on expression levels and/or other “omics” traits. Extensive evaluations of genetic effects on omics traits such as gene expression [6], protein abundance [7], DNA methylation [8], histone modification [9, 10], and RNA splicing [11] have revealed an abundance of quantitative trait loci (QTLs) for omics traits (omics QTLs) throughout the genome. These findings suggest that integrating data from omics and multi-omics QTL studies with GWAS would help to elucidate functional mechanisms that underlie trait/disease processes. Moreover, the integrative analysis of omics and multi-omics traits would also enhance confidence in detecting true omics associations while reducing false-positive findings by observing co-occurrence of associations in multiple different data types and borrowing information across multi-omics data sources. The increasing availability of summary statistics for complex traits and omics QTL studies in many conditions and cellular contexts [6, 12–14] provides a valuable resource to conduct integrative analyses in a variety of settings and presents an unprecedented opportunity to gain a system-level perspective of the regulatory cascade, which may highlight targets for disease prevention and/or treatment strategies.

To integrate GWAS and omics QTL summary statistics, several methods have been proposed to identify trait-associated loci that share a common casual variant with omics QTLs (often referred to as “colocalization”) [15–18]. Most of these methods allow for integration of GWAS summary statistics with one or few sets of QTL summary statistics [15, 17, 18]. There are also methods that have been proposed to directly test the molecular mechanisms through which genetic variation affects traits by integrating GWAS and eQTL summary statistics [19, 20]. By applying the integrative methods to multi-omics data, some QTL pairs such as eQTL and methylation (me)QTL pairs have also been identified with evidence of a shared causal mechanism [16, 21]. Integrating studies of multiple complex and omics traits could produce a more comprehensive picture of how cellular processes contribute to variation in complex traits.

Compared to integrating GWAS with single omics QTL statistics, studying multi-omics QTLs increases the chances of detecting the regulatory mechanisms underlying trait/disease-associated SNPs. The effect of any particular SNP may be strong for some omics traits and weak or absent for others. For example, protein (p)QTLs exist for genes lacking an apparent eQTL [22], suggesting post-transcription regulation [23]. And there could be multiple different omics QTLs in a gene region with different functions. As another example, SNPs affecting RNA splicing (splicing QTLs) may not be eQTLs in a gene region [11]. Moreover, QTL effects may vary across molecular phenotypes [24], tissue types [6], cell types [25, 26], or other contexts [27, 28]. For example, lead SNPs for eQTLs (eSNPs, and a “lead SNP” is the SNP with the smallest association *P* value with a particular trait in the region) often vary by tissue type [6]. Jointly analyzing the omics QTL association summary statistics to more than one type of omics trait from different conditions/studies could yield a more complete portrait of the regulatory landscape. Given the increasing availability of summary statistics for omics QTLs from different studies/conditions/cell contexts, novel methods and tools are needed to integrate GWAS with many relevant sets of omics QTL summary statistics for an improved understanding of the mechanisms of trait-associated SNPs.

Jointly analyzing multiple complex and omics traits can also be viewed as an approach for identifying shared mechanisms that underlie multiple complex traits—pleiotropic effects. Pleiotropy is ubiquitous in the genome [29, 30]. Since pleiotropic effects often occur among related diseases and traits [31–33], shared mechanisms are likely to exist. By integrating omics QTL summary statistics from multiple trait-relevant tissue types with GWAS statistics, one can also boost power in detecting pleiotropic effects while simultaneously providing mechanistic interpretations.

**Fig. 1:**
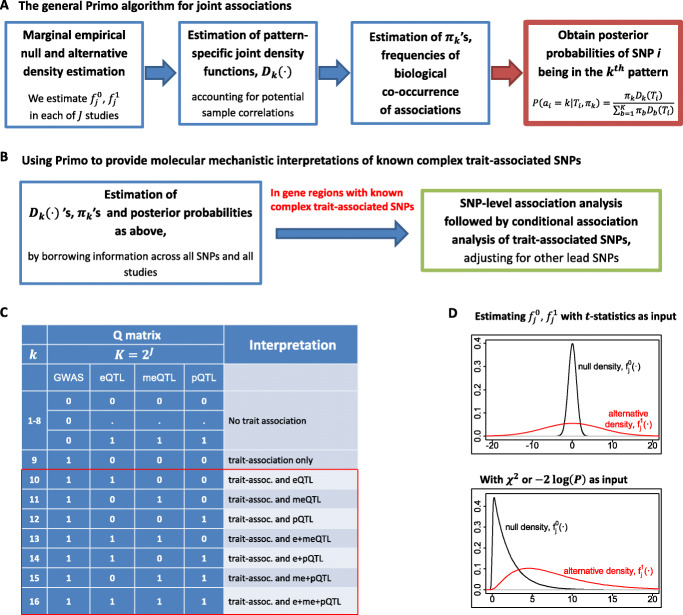
An overview of Primo and an illustration of interpretations of results. **a** The main steps of the Primo algorithm for assessing joint associations. **b** Steps of the Primo algorithm to provide mechanistic interpretations of known complex trait-associated SNPs. **c** An example—the **Q** matrix and interpretations of association patterns for an analysis of complex trait, eQTL, meQTL, and pQTL studies for *j* = 1, 2, 3, and 4, respectively. The red box shows how association patterns can be collapsed into groups of interest (here, summing probabilities across the patterns in the red box would yield the probability of association with the complex trait and *at least one* omics trait). **d** An example of the estimated marginal null and alternative densities of a moderated *t*-distribution (top) and *χ*^2^ or −2 log(*P*) values (bottom) for a study *j*

Given the rich availability of omics and multi-omics QTL summary statistics and their dynamic effects in different cellular conditions, in order to provide a comprehensive mechanistic interpretation of known trait-associated SNPs, it is desirable to develop new methods that can integrate multiple sets of GWAS statistics and omics QTL statistics from different conditions/studies while accounting for study heterogeneity, potential sample correlations, and linkage disequilibrium (LD). Additionally, as the number of traits/studies/conditions being considered grows, it will be more likely to detect joint associations by chance, necessitating proper multiple testing adjustment. To address those challenges, in this work, we develop a method to integrate summary statistics from multiple GWAS and omics QTL studies, and implement the method in an R package—Primo (Package in R for Integrative Multi-Omics association analysis). Figure 1a provides an overview of the algorithm.

Primo is flexible in many aspects: it allows unknown and arbitrary study heterogeneity and can detect coordinated effects from multiple studies while not requiring the effect sizes to be the same, it allows the summary statistics to be calculated from studies with independent or overlapping samples with unknown sample correlations, and it is not an omnibus test for association, but rather can be used to calculate the probability of each SNP belonging to each type (or groups) of interpretable association patterns (e.g., the probability of a trait-associated SNP also being associated with at least one/two cis omics traits). For gene regions harboring known susceptibility loci, the conditional association analysis of Primo examines the conditional associations of a known trait-associated SNP with other complex and omics traits adjusting for other lead SNPs in a gene region. It moves beyond joint association towards colocalization and provides a thorough inspection of the effects of multiple SNPs within a region to reduce spurious associations due to LD (Fig. 1b).

We conduct extensive simulations to evaluate the performance of Primo under various scenarios in analyzing multiple sets of summary statistics from studies with correlated samples. We apply Primo to examine the omics trait association patterns for known SNPs associated with breast cancer risk by integrating multi-omics QTL summary statistics from the Genotype-Tissue Expression (GTEx) project [6] and The Cancer Genome Atlas (TCGA) [34] with GWAS statistics from The Breast Cancer Association Consortium (BCAC) [35]. We also apply Primo to detect known trait-associated SNPs with pleiotropic effects to two complex traits in gene regions harboring susceptibility loci for at least one trait, while also providing mechanistic interpretations by integrating publicly available GWAS summary statistics [36–39] with multi-tissue eQTL summary statistics from GTEx. In this work, we focus on only trait-associated SNPs and aim to provide comprehensive mechanistic interpretations of how known GWAS SNPs affect complex traits. It should be noted that the goal of the analyses is not to identify true causal SNPs. However, the Primo algorithm is generally applicable to integrative association analysis, and when applied in other contexts, the interpretations of the results may be different.

## Results

### Primo as a general framework for assessing joint associations across data types

Here, we first introduce the general Primo association framework (Fig. 1a) and then discuss the tailored development in using Primo to provide mechanistic interpretations of known trait-associated SNPs (Fig. 1b), moving from association to colocalization. As a general integrative association method, Primo takes as input multiple sets of association summary statistics from different studies of different data types. The multiple sets of summary statistics could be one set of GWAS statistics and multiple sets of omics/multi-omics QTL statistics, or two or more sets of GWAS statistics of related traits and multiple sets of omics/multi-omics QTL statistics from trait-relevant tissue types, or could even be from studies beyond the complex and omics trait associations of germline variation.

Consider an *m*×*J* matrix of association statistics, **T**, consisting of the summary statistics for the associations of *m* SNPs with *J* types of traits from *J* studies with independent or correlated samples. Note that here, a “study” refers to a study of SNPs’ associations to a particular trait in a particular condition/cell type/tissue type. For each SNP (here a row in the matrix **T**), the underlying association status to the *j*th (*j*=1,…,*J*) trait is binary. Considering all SNPs in the genome, there are a total of *K*=2^*J*^ possible association patterns to *J* traits. We use a *K*×*J* binary matrix, **Q**, to denote all of the possible association patterns. And *q*_*kj*_=1 implies *the presence of association* with the *j*th trait in the *k*th association pattern, and *q*_*kj*_=0 implies *no association*.

For each SNP *i*, there must be one and only one true underlying association pattern. Primo calculates the probability of a given SNP being in each of the *K* mutually exclusive association patterns by borrowing information across SNPs in the genome and across *J* traits. More specifically, let *a*_*i*_ denote the true association pattern for SNP *i*. Then, the probability that SNP *i* belongs to association pattern *k* is given by:

1 $$  P\left(a_{i} = k|T_{i},\pi_{k}\right) = \frac{\pi_{k} D_{k}(T_{i})}{{\sum\nolimits}_{b=1}^{K} \pi_{b} D_{b}(T_{i})},  $$

where *T*_*i*_ is a vector of *J* association statistics and is also the *i*th row in the **T** matrix, *π*_*k*_ represents the overall proportion of SNPs in the genome belonging to the *k*th association pattern (*k*=1,…,*K*), and *D*_*k*_(·) is the multivariate density function of *J* sets of statistics, conditioning on the *k*th association pattern. Here, *π*_*k*_ captures the biological co-occurrence frequency of the *k*th association pattern in the genome, with $\sum _{k} \pi _{k} =1$. For example, in Fig. 1c, *π*_16_ is the proportion of SNPs in the genome that are associated with all of the three omics traits and the complex trait.

In estimating a mixture distribution of *K* components, the performance of estimation and subsequent inference depend on how well different mixing components separate from each other. When *K* is moderate to large, it is challenging to simultaneously estimate the distributions of mixing components (*D*_*k*_’s) and the mixing proportions (*π*_*k*_’s). Different from previous work [40], Primo first estimates the pattern-specific multivariate density function *D*_*k*_ for each of the association pattern by borrowing information across SNPs and traits. See the “Methods” section for detailed estimation procedures when *J* sets of association statistics were calculated from independent or correlated samples as well as discussion of two versions of the method for integrating *t*-statistics or *P* values, respectively. Then, Primo estimates *π*_*k*_’s via the Expectation-Maximization algorithm [41]. When *D*_*k*_’s are reasonably estimated, the one-step estimates of *π*_*k*_’s can well capture the overall proportions of different association patterns and there is no need to re-iterate and re-estimate *D*_*k*_’s and *π*_*k*_’s. Based on (1), we can obtain the posterior probabilities of SNP *i* being in each of the *K* possible association patterns.

Note that the *t*-statistic-based Primo—Primo(*t*)—and the *P*-value-based Primo—Primo(*P*)—may produce slightly different results due to different estimation algorithms of the *D*_*k*_’s. Primo(*t*) requires both effect sizes and standard errors as input. When those statistics are not available, or when *F* tests or other second-order tests are used in association analysis, or when one-sided tests are preferred if a same direction of association effects is expected for biological reasons, then users may instead use Primo(*P*).

An advantage of Primo is that one may collapse many association patterns based on biological interpretations and obtain the posterior probabilities of groups of patterns of interest by summing over the probabilities of those mutually exclusive patterns. As illustrated in Fig. 1c, when *J*=4, there are 16 possible association patterns. We may collapse the association patterns into interpretable groups. For example, here, we are interested in the trait-associated SNPs that are also associated with at least 1 omics trait. And we can obtain the probability estimate by summing over the posterior probabilities of patterns 10–16. When *J* is large, some specific patterns might not be present in the genome. With collapsed patterns, this would not be an issue, and both interpretability and robustness of the results are enhanced.

For a pattern of interest, we can also calculate the estimated false discovery rate (FDR) [42] for multiple testing adjustment:

2 $$\begin{array}{@{}rcl@{}}  \textrm{estFDR}(\lambda) = \frac{{\sum\nolimits}_{i} \left(1-\hat P_{i}\right)1\!\left(\hat P_{i}\geq \lambda\right)}{\#\left\{\hat P_{i}\geq \lambda\right\}}, \end{array} $$

where *λ* is the probability threshold and $\hat P_{i}$ is the estimated probability of SNP *i* being in the (collapsed) pattern of interest.

### Mechanistic interpretations of trait-associated SNPs via Primo conditional association analysis in gene regions harboring susceptibility loci

In order to elucidate the molecular mechanisms of known trait-associated SNPs, one may examine the omics trait associations of those SNPs by integrating GWAS and omics QTL summary statistics. However, a major challenge in such analyses is the complex LD structure among SNPs in the same gene region.

**Fig. 2:**
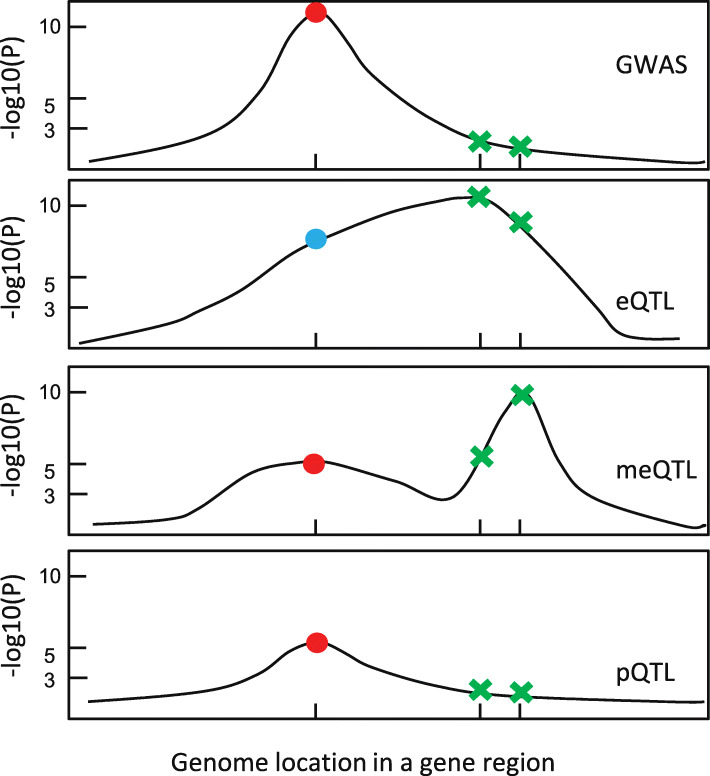
A conceptual illustration of the conditional association analysis of Primo. Consider a joint analysis of GWAS summary statistics and summary statistics of eQTL, meQTL, and pQTL. In a gene region harboring trait-associated SNPs, there is a GWAS SNP of interest (red/blue dot) and two other confounding SNPs—the lead SNPs for eQTL and meQTL (green cross). Before conditional association analysis, the GWAS SNP is estimated to be associated with cis expression, methylation, and protein levels. After adjusting for the two lead omics SNPs, the GWAS SNP is no longer associated with cis expression levels (blue dot) but is still estimated to be a me- and pQTL

To assess whether a GWAS SNP is associated with omics traits not due to it being in LD with other lead omics SNPs, we propose to conduct conditional association analysis within gene regions harboring susceptibility loci, with summary statistics of the GWAS SNP and other lead omics SNPs as input. Here, we consider a GWAS SNP *i* of interest and a set of lead omics SNPs *I*^′^ in the gene region, where *I*^′^ is a set of indices. We can model the joint association statistics for SNPs *i* and *I*^′^ in study *j* using a multivariate normal distribution and further calculate the conditional density functions of SNP *i* adjusting for other lead omics SNPs given their most plausible association patterns. See the “Methods” section for details. Then, with the estimated *π*_*k*_’s, we can assess the probabilities of associations for SNP *i* in (1). Figure 2 shows a conceptual illustration of the conditional association analysis. If the GWAS SNP is an independent meQTL and pQTL, it remains associated with methylation and protein after adjusting for other lead SNPs in the region, and if the GWAS SNP is associated with cis expression levels because it is in LD with the lead eSNP, it will be no longer significantly associated with expression after adjusting for the lead eSNP. With conditional association analysis, we can reduce spurious associations due to LD.

As a summary, to elucidate the molecular mechanisms of trait-associated SNPs, we first obtain the estimates of key parameters (*π*_*k*_’s, *D*_*k*_’s) by borrowing information across all SNPs and across traits/studies. Then, we focus on each gene region harboring known trait-associated SNPs and conduct a SNP-level association analysis to all traits for all SNPs in the gene region, followed by a conditional association analysis for each GWAS SNP of interest accounting for LD with other lead omics SNPs. If a GWAS SNP is no longer associated with a particular omics trait after conditioning on the lead omics SNPs, we will not consider it as being truly associated with the omics trait, i.e., the GWAS SNP is not affecting the complex trait via modulating the omics trait. Estimated FDR can be calculated as described.

In the “Methods” section, we also discuss extensions of Primo when the number of traits being considered is large (>15). We implemented Primo in Rcpp. It is computationally efficient and can analyze the associations of 30 million SNPs to five sets of complex and omics traits within 30 min on a single machine with 32 GB of memory and a 3-GHz processor.

### Simulation studies to evaluate the performance of Primo

We evaluated the performance of Primo in a variety of simulated scenarios. In each scenario, we simulated genotypes and phenotypes for 1000 subjects and calculated the test statistics for associations of SNPs with *J* traits. To simulate phenotypes from the simulated genotypes, we grouped SNPs into gene regions of *m*^′^=500 SNPs. In each gene region, the phenotypes of *J* traits for subject *s* were generated based on additive genetic models. SNPs’ effect sizes under the alternative were simulated from a normal distribution with mean 0 (allowing effect sizes to be positive or negative), and error terms are potentially correlated. Test statistics were estimated using single-variant linear regression. The simulated data structure and test statistic distributions mimic what we have observed in the eQTL data from GTEx. For each simulated dataset, we ran two versions of the Primo algorithm, Primo(*t*) and Primo(*P*), respectively. We repeated each simulation 100 times and compared the performance of the two versions of Primo versus competing methods (if applicable).

#### Accurate estimation of proportions (**π**) even for very sparse joint associations

It is known to be challenging to estimate *π*_*k*_’s when associations are sparse, i.e., *π*_*k*_’s being very close to zero for patterns with associations. In scenarios 1a and 1b, we showed that in analyzing correlated sets of summary statistics, when true associations are sparse and very sparse, respectively, Primo can well estimate the *π*_*k*_’s. In each scenario, we simulated test statistics for *J*=3 traits for 10 million SNPs, first under independence and then with pairwise (Pearson) correlation of 0.2 between traits. Non-zero effect sizes were simulated from a standard normal distribution. In scenario 1a, we simulated true *π*_*k*_=(7×10^−4^, 2×10^−4^, 1×10^−4^) for SNPs being associated with only one, exactly two, and all three traits, respectively. Scenario 1b simulated even sparser associations for the third trait, with *π*_*k*_=(7×10^−6^, 2×10^−6^, 1×10^−6^) for SNPs being associated with only the third, the third and first or second, and all three traits, respectively. Table 1 shows true *π*_*k*_’s and the average estimates for *π*_*k*_’s by Primo based on *t*-statistics or *P* values. In Additional file 1: Table S1, we also show the performance of estimation of *π*_*k*_’s when the marginal alternative proportions $\theta ^{1}_{j}$’s are highly mis-specified. As shown, Primo estimates the *π*_*k*_’s with reasonable accuracy even when the associations are very sparse and when the marginal alternative proportions $\theta ^{1}_{j}$’s are under-specified.

**Table 1:** Average estimates of $\hat {\pi }$

Scenario	Method	*π*_*k*_(*%*)							
		*q*_*k*_ = (0 0 0)	(1 0 0)	(0 1 0)	(0 0 1)	(1 1 0)	(1 0 1)	(0 1 1)	(1 1 1)
1a		Independent							
	True	99.720	0.070	0.070	0.070	0.020	0.020	0.020	0.010
	Primo(*t*)	99.712	0.074	0.074	0.074	0.019	0.019	0.019	0.010
	Primo(*P*)	99.749	0.065	0.065	0.065	0.016	0.016	0.016	0.007
		Correlated							
	True	99.720	0.070	0.070	0.070	0.020	0.020	0.020	0.010
	Primo(*t*)	99.712	0.073	0.073	0.073	0.019	0.019	0.019	0.011
	Primo(*P*)	99.749	0.065	0.065	0.065	0.016	0.016	0.016	0.007
1b		Independent							
	True	99.839	0.070	0.070	0.0007	0.020	0.0002	0.0002	0.0001
	Primo(*t*)	99.832	0.073	0.073	0.0031	0.019	0.0002	0.0002	0.0001
	Primo(*P*)	99.857	0.063	0.063	0.0014	0.016	0.0002	0.0001	0.0001
		Correlated							
	True	99.839	0.070	0.070	0.0007	0.020	0.0002	0.0002	0.0001
	Primo(*t*)	99.832	0.073	0.072	0.0029	0.019	0.0002	0.0002	0.0001
	Primo(*P*)	99.857	0.063	0.063	0.0013	0.016	0.0002	0.0002	0.0001

Scenario 1a simulates sparse associations for *J*=3 traits. Scenario 1b simulates even sparser associations for the third trait

#### Comparison with existing methods for jointly analyzing associations to three traits

In scenario 2, we simulated genotypes and phenotypes with pairwise sample correlations of 0.2 among *J*=3 studies for 1 million SNPs. The proportions of SNPs associated with only one, exactly two, and all of the three traits were 5×10^−3^, 5×10^−4^, and 5×10^−4^, respectively. Non-zero effect sizes were simulated from a *N*(0,*σ*^2^) distribution, with *σ*^2^ = 0.25, 0.5, and 1.0 each in one third of gene regions. We then calculated the SNP-level test statistics and *P* values as input for Primo.

Here, we compared the true and estimated FDRs and power to detect associations to all three traits and to at least one trait, based on Primo versus two competing methods, “moloc” [16] and Fisher’s method [43]. The results with correctly specified, under-specified (by 10-fold), and over-specified (by 10-fold) marginal non-null proportions ($\theta ^{1}_{j}$’s) are shown in Table 2. When $\theta ^{1}_{j}$’s are well-specified (scenario 2a in Table 2), Primo nicely controlled the FDR even in the presence of unknown study/sample correlations—highlighting one advantage of Primo in integrating potentially correlated multi-omics data. Note that moloc and Primo are not directly comparable as moloc aims to assess whether multiple traits of interest share a causal variant in a gene region, while Primo first identifies SNPs’ joint associations to multiple traits and then reduces spurious associations due to LD. Nevertheless, we show comparisons between Primo and moloc in the simulated setting. Since moloc does not output the posterior probabilities for all SNPs in every association pattern, we are only able to compare the power and FDR of Primo versus moloc in detecting associations to all three traits. We observed that Primo generally enjoys substantial power improvement, which is not surprising because the goal of moloc is more restrictive. As shown in Table 2, the estimated FDR (estFDR) is very close to the true FDR for Primo. Fisher’s method, as a combination method for testing omnibus hypotheses, can only be used to detect SNPs with associations to at least one trait and is not applicable to detect associations to all traits. The estimated FDR [42, 44] for Fisher’s method based on nominal *P* values is not well controlled due to correlations among test statistics, as expected. At similar power levels, the FDRs observed across simulations of Fisher’s method are also much higher than those of Primo.

**Table 2:** Simulation results evaluating the performance of Primo. *PP* posterior probability, *estFDR* estimated FDR. (A) When *J*=3 with correlated samples, we compared Primo versus moloc and Fisher’s method in detecting associations to at least 1 trait and associations to all traits and when parameters are correctly, under-, and over-specified. (B) When *J*=5 with correlated samples, we evaluated the performance of Primo

	

In this simulation, the true $\theta ^{1}_{j}$’s are 2.5×10^−3^. In scenario 2b, we under-specified $\theta ^{1}_{j}$ to be $\theta ^{1}_{j}/10$. As shown in Table 2 (A), although power might decrease to some extent, the FDRs are reasonably controlled. In scenario 2c, when $\theta ^{1}_{j}$’s are over-specified by an order of magnitude as $10\times \theta ^{1}_{j}$, we observed slightly inflated FDRs. As such, we suggest to obtain reasonable estimates for $\theta ^{1}_{j}$’s based on the current data and the literature, or under-specify $\theta ^{1}_{j}$’s to be more conservative. When $\theta ^{1}_{j}$’s are correctly or under-specified in a certain range, Primo is robust to parameter specification.

#### The performance of Primo in jointly analyzing more than three traits

In scenario 3, we simulated genotypes and phenotypes with pairwise sample correlations of 0.2 among *J*=5 studies for 1 million SNPs. Non-zero effect sizes were simulated from a standard normal distribution. *π*_*k*_=5×10^−4^ for the patterns where SNPs are associated with one trait, and *π*_*k*_=1×10^−4^ for the patterns where SNPs are associated with two, three, four, and all of the five traits, respectively. We then calculated the SNP-level test statistics and *P* values as input for Primo. The results of Primo analyses are presented in Table 2 (B). Overall, Primo yields good control of FDRs and high power in detecting various patterns of joint associations, even for a moderately large number of sets of summary statistics and in the presence of study correlations.

#### Evaluation of the performance of Primo conditional association analysis accounting for LD and sample correlations

In this section, we simulated association statistics for correlated SNPs in moderate to high LD and evaluated the performance of the proposed conditional association approach in the presence of LD. To simulate genotype data with a realistic LD structure, we used the sim1000G package [45] to simulate 1 million variants for 1000 subjects using chromosomes 8, 9, and 10 in the CEU 1000 Genomes population [46]. We divided the genotypes into regions of 1000 consecutive SNPs in order to form gene regions. Within each region, we randomly selected one SNP with MAF >0.1 to be the “known trait-associated SNP” and randomly selected two “confounding SNPs” in moderate to strong LD with both the trait-associated SNP and each other (pairwise *r*∈[0.5,0.8]). Within each gene region, we then generated *J*=4 traits. The first trait is a “complex trait” for all 1000 subjects, and the three other traits are “omics traits” with sample sizes of 500, 300, and 200, respectively, resampled from the 1000 subjects. In 20% of the LD blocks, the true underlying association pattern for the trait-associated SNP is (1,0,1,0) while the association patterns for the two confounding SNPs are (1,1,0,0) and (0,1,1,1), respectively. These LD blocks represent gene regions with no SNP truly associated with all traits but with multiple SNPs in LD with different association patterns. The effect sizes in these blocks ranged from 0.1 to 0.4. We further simulated another 20% of LD blocks where the true underlying association pattern for the trait-associated SNP is (1,1,1,1) while the association patterns for the two confounding SNPs are (0,1,0,0) and (0,0,1,0), respectively. These LD blocks represent gene regions with one true causal SNP associated with all traits as well as two confounding SNPs in high LD with it. For the remaining 60% of the LD blocks, no SNPs are associated with any traits. Then, we obtain the single-variant association statistics **T** for 1 million SNPs with *J*=4 traits.

We applied Primo with **T** as input to identify SNPs associated with all traits. For each index SNP detected as significant at the probability cutoffs of 0.8 and 0.9, we further conducted conditional association analysis, conditioning on its two confounding SNPs in moderate to high LD. The trait-associated SNPs that no longer have the highest probabilities in the pattern of (1,1,1,1) after conditional association analysis were not considered to be positive findings. In the calculations of the FDRs, we use the same denominators before and after conditional association analysis for fair comparison. That is, the denominators are the number of identified SNPs with associations to all traits at a given cutoff before the conditional associating analysis. After conditional analysis, the numerator (i.e., # false positive) of the true FDR is the number of SNPs that are not truly associated to all traits, yet continue to show the highest probability in the pattern of (1,1,1,1) after conditional association analysis. In the calculation of the numerator of the estimated FDR, for each SNP *i* that is no longer significant after conditional analysis, its contribution to the numerator $\left (1-\hat P_{i}\right)1\!\left (\hat P_{i}\geq \lambda \right)$ in the formula (2) is corrected to be 1 since we considered it as an estimated false discovery.

Table 3 summarizes the results over 100 simulations. As shown in the table, when SNPs are in LD, we observed some slightly inflated FDRs without conditional association analysis even when $\theta _{j}^{1}$’s are correctly specified (scenario 4a). In contrast, after accounting for LD, true FDRs are reduced and are well controlled by the estimated FDRs. In scenarios 4b and 4c, we under-specified and over-specified $\theta _{j}^{1}$’s by 10-fold. Overall, Primo after conditional association analysis could yield nice control of FDR and maintain good power when $\theta _{j}^{1}$’s are correctly or under-specified.

**Table 3:** Comparison of results before and after conditional association analysis. *PP* posterior probability

Scenario	PP ≥0.9						PP ≥0.8					
	Before accounting for LD			After accounting for LD			Before accounting for LD			After accounting for LD		
	True	estFDR	Power	True	estFDR	Power	True	estFDR	Power	True	estFDR	Power
	FDR (%)	(%)	(%)	FDR (%)	(%)	(%)	FDR (%)	(%)	(%)	FDR (%)	(%)	(%)
4a	5.1	2.5	71.3	4.1	4.7	70.3	8.7	4.6	82.8	6.4	8.6	80.8
4b	3.8	2.4	67.4	2.9	5.6	65.7	7.0	4.6	79.2	4.7	10.1	75.9
4c	7.6	2.7	76.4	6.9	3.4	76.3	13.6	5.0	88.5	11.8	7.0	88.0

### Application I: Understanding the mechanisms of breast cancer susceptibility loci

With over 100,000 breast cancer cases and a similar number of controls, BCAC [35] has recently reported 174 common genetic variants associated with breast cancer risk. In order to understand the underlying mechanisms of those susceptibility risk loci and their potential cis target genes, a recent study [47] conducted cis-eQTL analysis using both normal and tumor breast transcriptome data and identified multiple genes likely to play important roles in breast tumorigenesis.

In addition to transcription, SNPs may affect cis-epigenetic features, protein abundances, and other omics traits. Functional relationships may exist among those omics traits. Therefore, we propose to jointly examine the susceptibility risk loci and their effects on multiple omics traits in tumor and normal tissues in order to better understand the mechanisms through which risk-associated SNPs act in different conditions. Moreover, this analysis will enhance our understanding of the regulatory cascade and their roles in breast tumorigenesis. The regulatory SNPs with “cascading effects” [22, 48] on gene regulation and downstream gene products are of particular interest.

In this work, we applied Primo to integrate GWAS summary statistics from BCAC with the eQTL, meQTL, and pQTL association summary statistics obtained from 1012, 762, and 74 breast tumor samples, respectively, from TCGA [34, 49] (see Additional file 1) and eQTL summary statistics obtained from 396 normal breast mammary samples from GTEx [50]. A total of 162 of the GWAS SNPs reported by Michailidou et al. [35] reached genome-wide significance (*P*<5×10^−8^) in the meta-analysis. And there are 158 of these SNPs with MAF >1*%* in TCGA data. And the 158 breast cancer GWAS SNPs are the SNPs we examined for mechanistic interpretations, while we used genome-wide summary statistics from all SNPs to obtain estimations of key parameters. Note that one SNP could be mapped to multiple genes and multiple CpG sites. We assessed the probabilities of 32 (2^5^, for GWAS and 4 omics QTLs) association patterns for each SNP-gene-CpG-protein quartet. In the conditional association analysis of gene regions harboring at least one GWAS SNP, we selected the lead SNP for each omics trait in the region and adjusted for any lead SNP outside a 5 kb distance of and with LD *R*^2^<0.9 with the GWAS-reported SNP (those with *R*^2^>0.9 or within 5 kb were considered likely to share a causal variant or too close to assess individual associations, respectively).

**Fig. 3:**
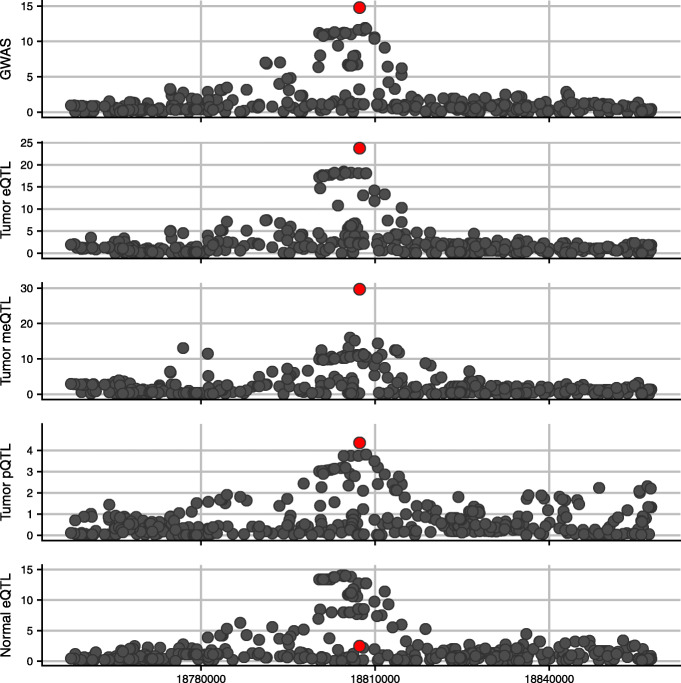
An example of a known breast cancer susceptibility locus being associated with multi-omics traits. At a posterior probability threshold of 80%, Primo identified SNP rs2992756 as being associated with all four omics traits for the gene *KLHDC7A*. Here shows the − log10(*P*) values by position on chromosome 1 in the region of the gene *KLHDC7A* for all SNPs including the breast cancer susceptibility locus (rs2992756, red dot) in GWAS (top panel) and eQTL, meQTL, and pQTL analyses in tumor tissue (the next three panels, respectively) and eQTL analysis in normal tissue (bottom panel) for the gene and protein KLHDC7A and CpG site cg05040210

At the 80% probability cutoff and after conditional association analysis (estimated FDR of 4.2, 9.6, 20.2, and 13.2%), there were 52, 26, 9, and 1 GWAS SNPs out of 158 examined being associated with at least 1, 2, 3, or 4 omics traits, respectively. The three GWAS SNPs (rs11552449, rs3747479, and rs73134739) in the three genes (*DCLRE1B*, *MRPS30*, and *ATG10*, respectively) reported in Guo et al. [47] had high probabilities of being an eQTL in both tumor and normal tissues (with probabilities of 61.1, 95.6, and >99.9%, respectively). In the *KLHDC7A* gene region, the GWAS SNP rs2992756 (indicated by red dot in Fig. 3) is associated with the expression, methylation, and global protein abundance levels of the cis-gene *KLHDC7A*. Figure 3 shows the plot of − log10(*P*) values of associations to breast cancer risk and the three omics traits (with expression traits in both tumor and normal tissue types) of *KLHDC7A* for the SNPs in the gene region. Note that the GWAS SNP is only moderately associated with the gene expression levels in the normal GTEx breast tissue with a *P* value of 0.0034, highlighting the need to study omics QTLs under different conditions.

**Fig. 4:**
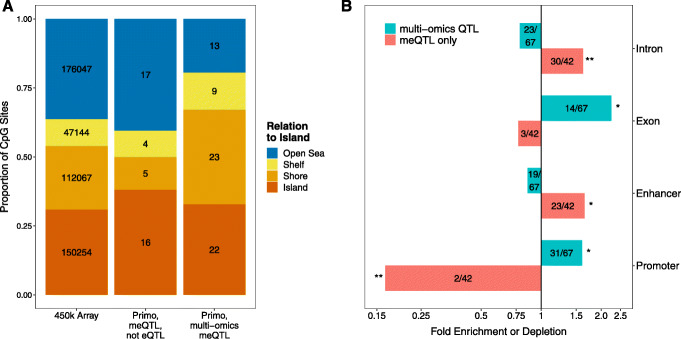
Enrichment of CpG targets of breast cancer susceptibility loci among genomic features. **a** Distribution with relation to islands of CpG targets of Primo-identified multi-omics QTLs and meQTL-only susceptibility loci compared with distribution of all CpGs on 450k array. Numbers represent counts of CpGs in each relationship to islands. **b** Fold enrichment or depletion of genomic features among CpG targets of multi-omics QTL (cyan) and meQTL-only (pink) susceptibility loci. Feature counts out of total CpG targets are displayed within each bar. *X*-axis displayed on log scale. *P* values were obtained by bootstrapping samples of CpGs from the full 450k array [* *P*<0.01, ** *P*<10^−3^]

Due to limited sample sizes (74) in the pQTL analysis, only 1 out of the 158 examined breast cancer susceptibility loci was associated with cis-protein abundance levels with high confidence, although the cis-gene expression levels and cis-protein abundances for those loci were often highly correlated with an averaged (Pearson) correlation coefficient of *r* = 0.396 and a median of *r* = 0.411. There were 16 out of 158 susceptibility loci uniquely associated with cis-methylation levels but not expression levels in either tumor or normal tissue, echoing a recent work showing both unique and shared causal mechanisms of epigenome variations and transcription [21]. We analyzed the CpG targets of meQTLs identified by Primo for enrichment in several genomic features. As shown in Fig. 4, CpG targets of multi-omics QTLs (breast cancer susceptibility loci associated with methylation as well as gene expression and/or protein abundance) were enriched in CpG Island Shores (*P*<0.05) and depleted in Open Seas (*P*<0.01). CpG targets of multi-omics QTLs were enriched in exons (*P*<0.01) while CpG targets of meQTL-only loci were enriched in introns (*P*<0.001). In promoter regions, CpG targets of multi-omics QTLs were enriched (*P*<0.01) while CpG targets of meQTLs not also associated with gene expression levels were depleted (*P*<0.001), consistent with the involvement of promoter regions in transcription. This also shows that the integration of GWAS and multi-omics traits can provide additional insights in understanding the complex and dynamic mechanisms.

### Application II: Detecting SNPs with pleiotropic effects and elucidating their mechanisms

Many genetic variants are associated with more than one complex trait [29, 30, 51]. Identifying such pleiotropic variants and elucidating the molecular mechanisms which underlie these multi-trait associations may enhance our understanding of the etiology of complex traits and provide additional insights into clinical treatment development [51]. In this section, we applied Primo to detect SNPs with pleiotropic effects to two complex traits in gene regions harboring susceptibility loci for at least one trait, and provide mechanistic interpretations by integrating pairs of publicly available complex-trait GWAS summary statistics with eQTL association summary statistics obtained from trait-relevant tissue types in the GTEx project.

We applied Primo to height [37] and body mass index (BMI) [38] GWAS summary statistics from the GIANT consortium (sample size >250,000) with eQTL summary statistics in subcutaneous adipose (*n*=581) and skeletal muscle (*n*=706) tissues from GTEx for all SNPs in the genome. There are 697 height-associated SNPs reported by Wood et al. [37] and 97 BMI-associated SNPs reported by Locke et al. [38]. Out of those SNPs reaching genome-wide significance (5×10^−8^) for either trait, 683 were present in both sets of GWAS summary statistics and could be mapped to GTEx SNPs in cis with at least one gene measured in both tissue types. Of the 683 SNPs, 612 reached genome-wide significance for height and 78 reached genome-wide significance for BMI, with 7 reaching genome-wide significance for both. Those 683 GWAS SNPs are the SNPs of interest in our analysis of pleiotropy, while again we estimated key parameters used in Primo using genome-wide summary statistics. At the 80% probability cutoff and after conditional association analysis accounting for LD, 32 SNPs out of 683 were detected by Primo as being associated with both complex traits (estimated FDR of 17.5%). Of these, 17 were associated with expression of at least one gene in at least 1 tissue (estimated FDR of 21.8%) and 12 were associated with expression of at least one gene in both tissues (estimated FDR of 18.4%). Furthermore, 12 of the SNPs were associated with the expression of multiple genes, highlighting the possibility that pleiotropic SNPs may affect multiple complex traits through their co-regulation of multiple genes.

To validate the 32 identified pleiotropic SNPs being associated with both height and BMI regardless of association status to cis-gene expression levels, we used GWAS summary statistics from the UK Biobank [39] (>336k samples have both height and BMI measured) as a replication study. At *P*<0.0008 (the Bonferroni threshold is calculated as 0.05/(32 ×2), since there are two traits), 27 out of the 32 SNPs were associated with both traits in the UK Biobank, including 16 of the 17 SNPs that were also associated with gene expression. Plots of −log_10_(*P*) values for associations with height, BMI, and expression in each tissue are presented in Additional file 1: Fig. S2 for the genomic regions containing the 27 replicated SNPs.

In Additional file 1, we also presented another set of analysis integrating GWAS summary statistics of Crohn’s disease and ulcerative colitis [36] with eQTL summary statistics from sigmoid colon (*n*=318) and transverse colon (*n*=368) tissues from GTEx. Both analyses showed that Primo can be used to detect SNPs with pleiotropic effects on (potentially more than two) complex traits while simultaneously providing mechanistic interpretations by examining their effects on cis-gene expression levels in trait-relevant tissue types. A majority of our detected and replicated pleiotropic SNPs do not have associations reaching genome-wide thresholds for both traits. Our analyses and results underscored the value of integrating GWAS summary statistics of multiple traits with eQTLs in relevant tissue types.

## Discussion

We proposed a general integrative genomics association approach—Primo—for assessing the joint associations across studies and data types, allowing for unknown study heterogeneity and sample correlation and taking only summary statistics as input. In the current work, we made a tailored development of Primo to comprehensively elucidate the molecular mechanisms of known complex trait-associated SNPs, where we assessed the omics or other trait associations of known complex trait-associated SNPs by conducting conditional association analysis in gene regions harboring known trait-associated SNPs to account for LD with other SNPs in the region. Note that in our analyses, we focused on known trait-associated SNPs reported in GWAS.

With the rapidly increasing availability of GWAS and omics QTL association summary statistics from different studies, populations, and cellular contexts, it is commonly observed that there could be multiple causal SNPs for different complex and omics traits in the same gene regions. Conducting integrative analysis of GWAS summary statistics and a limited number of sets of omics QTL statistics may provide only a partial view of the genomic activities in a region; meanwhile, if multiple omics QTL statistics are jointly analyzed, one also needs to consider the associations identified by chance and perform multiple testing adjustment. The advantage of Primo is that it can integrate a moderate to large number of sets of summary statistics from different data sources as input to provide a more comprehensive evaluation while also considering multiple testing adjustment. Additionally, Primo enjoys other unique advantages and shows great flexibility in integrative analysis. It allows the input summary statistics to be from independent or partially overlapped studies with unknown study correlations. It detects SNPs with coordinated effects allowing different effect sizes (and different directions of effect sizes) on different types of traits. It can also integrate one-sided *P* values if the same direction of effect sizes is expected and desired. Primo can identify SNPs in different combinations of association patterns to molecular omics and complex traits. Moreover, with the conditional association analysis of Primo, we can move one step beyond association towards causation by assessing whether a GWAS SNP is also an omics QTL while adjusting for the effects of multiple lead SNPs in a gene region. The conditional association analysis can reduce spurious omics trait associations of GWAS SNPs due to LD with the lead omics SNPs.

We implemented two versions of Primo taking either *t*-statistics (or effect sizes and standard error estimates) or *P* values as input. Primo is computationally very efficient and can analyze the joint associations of 30 million SNPs to five traits in dozens of minutes. We applied Primo to examine and interpret the associations to omics traits in tumor/normal tissues for known breast cancer susceptibility loci. We also applied Primo to integrate pairs of GWAS summary statistics of complex traits with eQTL summary statistics from trait-relevant tissue types from GTEx to detect pleiotropic effects and examine their mechanisms.

There are a few additional points we would like to emphasize. First, we recommend a stringent specification of the marginal study-specific alternative proportion parameters ($\theta _{j}^{1}$’s), especially when there is limited a priori knowledge guiding the parameter specification. Primo may suffer from slightly inflated FDR when those parameters are highly over-specified, whereas when those parameters are under-specified to an extent, there might not be much power loss. Second, the focus of the current work is to comprehensively evaluate the molecular mechanisms of known trait-associated SNPs, rather than to identify new causal SNPs for complex traits from other regions in the genome. When applying Primo in other integrative association analyses, the interpretations of results may be different. Third, there are many existing functional annotations for SNPs that are not incorporated in the current version of Primo but have also proved to be useful. We will explore this direction in future work. Last but not least, when jointly analyzing more than 15 sets of summary statistics, the computation time of Primo to assess all possible association patterns can increase substantially. The current work proposed a quick extension by applying Primo to groups of sets of summary statistics, while in a work-in-progress, we will develop an integrative analysis method for jointly analyzing dozens of sets of summary statistics.

Primo is motivated by the analysis of trait-associated SNPs for their molecular trait associations. It should be noted that Primo can also be broadly applied to many other settings when data integration is needed. Primo can be used to detect associations repeatedly observed in multiple correlated or independent conditions, and those repeatedly observed associations may enhance the confidence for new discoveries or at least provide a more comprehensive examination of how those associations may occur in different conditions.

## Methods

### Estimating empirical null and alternative marginal density functions for each of the *J* studies using the limma method

For each of the *J* studies, we first adopt the limma method [52, 53] to calculate a set of *moderatedt*-statistics by replacing the error variance estimates in the classical *t*-statistic calculation with the posterior variances. Here, for genetic association studies, we calculated the error variance for each SNP based on the *t*-statistic and the minor allele frequency (MAF) assuming that covariates are independent from genotypes. That is, the error variance for SNP *i* is given by $s_{ij}^{2} = \text {se}^{2}\!\left (\hat {\beta }_{ij}\right) \cdot 2 N_{j}(\textrm {MAF}_{i})(1-\textrm {MAF}_{i})$, where *N*_*j*_ is the sample size for study *j*. Alternatively, one may directly obtain the effect size estimate and its variance estimate as the summary statistics, if the information is available. The new variance shrinks the observed sample variance towards a prior that is estimated across all SNPs in the data, and stabilizes the variance estimation across the genome. It also penalizes the SNPs with large *t*-statistics but small variances.

Next, for each study *j*, we estimate the empirical null and alternative marginal density functions, $\hat f_{j}^{0}(\cdot)$ and $\hat f_{j}^{1}(\cdot)$, respectively, based on all the moderated *t*-statistics in the genome for the study. Here, one needs to specify a key parameter for each study, the proportion of study-specific non-null statistics (i.e., with associations), $\theta ^{1}_{j}$. Note that we used $\theta ^{1}_{j}=10^{-3}$ and 10^−5^ for omics QTL studies and GWAS, respectively, in the two applications. We then adopt the limma method to estimate $\hat f_{j}^{0}(\cdot)$ and $\hat f_{j}^{1}(\cdot)$ (illustrated in Fig. 1d). Under the null hypothesis, the moderated *t*-statistic follows a *t*-distribution with a mean of zero and moderated degrees of freedom *d*_*j*_ in the *j*th study, allowing for an empirical null distribution slightly deviating from the parametric *t*-distribution. Under the alternative, the moderated *t*-statistic follows a scaled *t*-distribution, still with degrees of freedom *d*_*j*_ and a mean of zero allowing for different directions of effects in different studies, and a SNP-specific scaling factor *v*_*ij*_ (*v*_*ij*_≥1) estimated from the data. The scaling factor is calculated as *v*_*ij*_=(1+*v*_0*j*_/*w*_*ij*_)^1/2^, where *v*_0*j*_ is the variance hyperparameter for the prior placed on non-zero effect size coefficients and *w*_*ij*_ is a SNP-specific weight for SNP *i*. In the presented analyses, we set *w*_*ij*_=1/(2*N*_*j*_·MAF_*i*_(1−MAF_*i*_)). The degrees of freedom *d*_*j*_ is estimated from the data as *d*_*j*_=*d*_0*j*_+*d*_1*j*_, where *d*_1*j*_ is the (original) degrees of freedom of the summary statistics in study *j* and *d*_0*j*_ is the degrees of freedom hyperparameter for the prior on the unknown variances of effect sizes. Estimation is performed using an empirical Bayes approach as described in Smyth [52] and implemented in the limma package in R [53]. With the estimated marginal null and alternative density functions from each study, the joint density functions for all *K* association patterns can be calculated as described in the next subsection.

### Estimating pattern-specific multivariate density functions when input summary statistics are calculated from independent or overlapping samples

With *J* independent studies, the pattern-specific multivariate density function *D*_*k*_ for the *k*th association pattern is given by:

3 $$  D_{k}(T_{i}) = \prod\limits_{j=1}^{J} f^{0}_{j}\!\!\left(t_{ij}\right)^{1-q_{kj}} f^{1}_{j}\!\!\left(t_{ij}\right)^{q_{kj}}.  $$

where *q*_*kj*_ is the association status of the *k*th pattern in study *j*. For example, given the association status being *q*_*k*_=(1,1,0,0), the joint density *D*_*k*_ is modeled as the product of the alternative marginal density functions from the first two studies and the null marginal density functions from the other two studies, $D_{k} = f_{1}^{1} \cdot f_{2}^{1} \cdot f_{3}^{0} \cdot f_{4}^{0}$.

In estimating a pattern-specific multivariate density function *D*_*k*_ from *J* correlated studies, we obtain the empirical null and alternative marginal distributions as non-scaled and scaled *t*-distributions, respectively, in each of the *J* studies. Then, we further approximate them with normal distributions with zero means and variances being $\sigma _{ikj}^{2}= v_{ij}^{2\times q_{kj}}\cdot \frac {d_{j}}{d_{j}-2}$, where *v*_*ij*_ is the scaling factor under the alternative. When the association status indicator *q*_*kj*_=0 for the *j*th study under pattern *k*, i.e., no association, $\sigma _{ikj}^{2}=\frac {d_{j}}{d_{j}-2}$. Since *J* studies are correlated due to possible sample overlap with an unknown correlation matrix of **Γ**, similar to Urbut et al. [54], we pool all the statistics likely to be from the null pattern to estimate their correlation matrix as the estimate for **Γ**. Under certain assumptions, the correlation matrix of test statistics approximates the sample correlation matrix and the sample correlation under the null represents the correlation due to sample overlap. Here, we estimate the *J*×*J* correlation matrix using SNPs with absolute statistics less than 5 in all *J* studies. Then, we approximate the pattern-specific multivariate density function *D*_*k*_ as $\mathcal {N}\left (\mathbf {0}, \mathbf {\Sigma }_{k}^{1/2} \mathbf {\Gamma } \mathbf {\Sigma }_{k}^{1/2}\right)$, where **Σ**_*k*_ is a diagonal matrix with diagonal elements of $\sigma _{ikj}^{2}$’s. Note that here, the normal approximations of multivariate density functions enjoy computational efficiency, and moreover, in the next section, they facilitate the estimation of conditional density functions. Also note that Primo separates sample correlations **Γ** from biological correlations/co-occurrences captured by *π*_*k*_’s in the subsequent estimation and inference.

### Conditional association analysis accounting for LD

To assess whether the trait association of a SNP *i* reflects an independent causal variant or is simply due to being in LD with a nearby lead SNP *i*^′^, conditional association analysis is often conducted [55]. It tests the conditional association of SNP *i* with the trait of interest adjusting for the genotype of the lead SNP *i*^′^ and other covariates. If SNP *i* is no longer statistically significant after adjusting for the lead SNP, it is unlikely that the trait association of SNP *i* reflects an independent causal effect.

Following this idea, to assess whether a GWAS SNP is associated with omics traits due to it being in LD with lead omics QTLs, we propose to conduct conditional association analysis with summary statistics of the GWAS SNP and lead omics QTLs as input. Here, we consider a GWAS SNP *i* of interest and a set of lead omics SNPs *I*^′^ in the gene region, where *I*^′^={1^′^,...,*L*^′^} is a set of indices. We can model the joint association statistics for SNPs *i* and *I*^′^ in study *j*, i.e., $\phantom {\dot {i}\!}\left (t_{ij}, t_{1'j},...,t_{L'j}\right)$, using a multivariate normal distribution, $\mathcal {N}\left (\mathbf {0}, \mathbf {\Lambda }_{j}\right)$, where **Λ**_*j*_ is the 1+*L*^′^ by 1+*L*^′^ variance-covariance matrix described as follows. The diagonal elements of **Λ**_*j*_ correspond to the study-specific variances of statistics of the SNPs. Specifically, the (1,1) entry of **Λ**_*j*_ is given by $\sigma ^{2}_{ij}$, which is the marginal variance of the statistic *t*_*ij*_ for SNP *i* in study *j* with $\sigma ^{2}_{ij}=\frac {d_{j}}{d_{j}-2}$ under the null and $\sigma ^{2}_{ij}=v_{ij}^{2}\cdot \frac {d_{j}}{d_{j}-2}$ under the alternative. For each lead SNP *i*^′^∈*I*^′^ with its most plausible association pattern $\phantom {\dot {i}\!}k_{i'}$, the variance of the corresponding *t*-statistic $\phantom {\dot {i}\!}t_{i'j}$ is given by $\sigma ^{2}_{i'k_{i'}j} = v_{i'j}^{2q_{k_{i'}j}}\cdot \frac {d_{j}}{d_{j}-2}$. The off-diagonal elements of **Λ**_*j*_ are calculated based on the study-specific variances of the SNPs and the LD among the SNPs assuming additional covariates are independent of the SNP genotypes [56]. For instance, the covariance between *t*_*ij*_ and $t_{i'j}\phantom {\dot {i}\!}$ is $\sigma _{ij}\cdot \sigma _{i'k_{i'}j}\cdot \rho _{ii'}\phantom {\dot {i}\!}$ where $\phantom {\dot {i}\!}\rho _{ii'}$ is the genotype correlation coefficient of the SNPs *i* and *i*^′^(∈*I*^′^). Partitioning the variance-covariance matrix **Λ**_*j*_ as follows, $\Lambda _{j} = \left (\begin {array}{ll}\Lambda _{j, 11} &\Lambda _{j, 12} \\ \Lambda _{j, 21} &\Lambda _{j, 22} \end {array}\right)$with sizes $\left (\begin {array}{ll} 1 \times 1 &1 \times L' \\ L' \times 1 & L' \times L'\end {array}\right)$, we can obtain the conditional null and alternative distributions for SNP *i* in study *j* as:

$$ t_{ij}\mid \left(\begin{array}{ll}t_{1'j} \\ \vdots \\ t_{L'j} \end{array}\right) \sim \mathrm{N}\left(\Lambda_{j, 12}\Lambda_{j,22}^{-1} \left(\begin{array}{ll}t_{1'j} \\ \vdots \\ t_{L'j} \end{array}\right), ~ \Lambda_{j, 11}-\Lambda_{j, 12}\Lambda_{j,22}^{-1}\Lambda_{j,21}\right)  $$

where $\Lambda _{j,22}^{-1}$ denotes the inverse of the matrix *Λ*_*j*,22_. Here, we approximate the conditional *t*-distributions with the conditional Gaussian distribution for efficient density estimation since most GWAS and omics QTL studies have sample sizes large enough for good approximation.

With the conditional null and alternative density functions for SNP *i* in study *j* adjusting for other lead omics SNPs in the region, we can proceed to obtain the pattern-specific *J*-variate density functions for all association patterns as outlined in the previous subsection and re-assess the probabilities of each association pattern in (1). We propose to conduct gene-level conditional association analysis accounting for LD structures only in selected gene regions, after the SNP-level association analysis.

### Primo for integrating *P* values from multiple studies

In addition to integrating *t*-statistics or effect sizes and variance estimates, Primo can also jointly analyze *J* sets of *P* values, chi-squared statistics, or other second-order association statistics. We model the pattern-specific multivariate density functions and still use Eq. (1) in obtaining the posterior probabilities for each SNP being in each pattern.

In estimating the marginal null and alternative density functions for each study *j*, $f^{0}_{j}$ and $f^{1}_{j}$ (as illustrated in Fig. 1d), we make the following modification. We first take negative two times the log of *P* values as our test statistics, **T**. Under the null hypothesis, *t*_*ij*_=−2 log (*p*_*ij*_) follows a $\chi ^{2}_{2}$ distribution. Under the alternative, the *P* value distributions may vary locus by locus. In the genome, the alternative distribution of −2 log (*p*_*ij*_)(*i*=1,…,*m*) follows a mixture of non-central chi-squared distributions, which can be approximated by a scaled chi-squared distribution with certain degrees of freedom, $A\chi _{d}^{2}$ [57, 58]. Note that we do not assume *P* values under the alternative follow the same distribution, rather we approximate the mixture of chi-squared distributions using a scaled chi-squared distribution. To estimate a study-specific scaling factor *A*_*j*_>0 and degree of freedom $d^{\prime }_{j}$ that best approximate the tail of the alternative distribution in study *j*, we use a numerical optimization algorithm to find values which minimize the differences between the *P* values of *T*_*j*_ under a mixture of $A_{j}\chi ^{2}_{d'_{j}}$ and $\chi ^{2}_{2}$ distributions given the mixing proportion $\theta ^{1}_{j}$ for the study, and their nominal *P* values based on their ranks.

More specifically, let *t*_*ij*_=−2 log (*p*_*ij*_) for SNP *i* in study *j*. Then, the cumulative distribution function of *t*_*ij*_ is given by:

$$ F\!\left(t_{ij} ; A_{j}, d'_{j}, \theta\right) = \left(1 - \theta^{1}_{j}\right) G\!\left(t_{ij} ; 2\right) + \theta^{1}_{j} G\!\left(\frac{1}{A_{j}} t_{ij} ; d'_{j}\right) $$

where *G*(·;*ν*) is the cumulative distribution function of a $\chi ^{2}_{\nu }$ variable. Let *r*_*ij*_ be the rank of SNP *i* in study *j* when the *t*_*ij*_ are sorted in descending order. To estimate *A*_*j*_ and $d^{\prime }_{j}$, we use the optimization algorithms implemented in the R nloptr package to minimize the following function [59]:

$$ \sum\limits_{i \colon r_{ij} \le \max\left\{20, \frac{m}{2}\theta^{1}_{j}\right\}} \left| 1 - F\!\left(t_{ij} ; A_{j}, d'_{j}, \theta^{1}_{j}\right) - \frac{r_{ij}-0.5}{m} \right|. $$

Since associations can be sparse (i.e., $\theta ^{1}_{j}$ being close to zero) in the genome, it is more important to well approximate the tail of the alternative distribution than the first two moments (mean and variance). As such, we sum over the most extreme tail statistics or at least the 20 most extreme statistics. In Additional file 1: Fig. S1, we have assessed the performance of the approximation via simulation studies, especially when associations are sparse. When the *J* studies are independent, the multivariate density function is modeled as the product of the individual density functions, as in Eq. (3). When the *J* studies are correlated, we proceed in a similar manner as when *t*-statistics are used as input, except that the multivariate normal distribution is replaced by the multivariate gamma distribution.

### Extensions of Primo when *J* is large

When jointly analyzing a large number of sets of association summary statistics, the number of possible joint association patterns *K*=2^*J*^ increases exponentially with the number of sets of statistics, *J*. When *J*=15, there are 32,768 possible association patterns and the calculation for all *K* patterns can be computationally expensive. One may reduce the number of patterns under consideration to only the major and interpretable patterns [54]. However, the selection of major and interpretable patterns is still a challenge. Additional work is still needed in future research. When analyzing a large number of sets of association statistics of similar types (for example, integrating multiple sets of eQTLs from different GTEx tissue types for cross-tissue eQTLs), one possible strategy is to group sets of statistics into major and independent groups *g*=1,…,*G*, each with *J*_*g*_<10 sets of statistics. Then, one can apply Primo to calculate the posterior probabilities within each group and take the products of the probabilities between groups to obtain the overall probabilities for all groups in the association patterns of interest. For example, the posterior probability of a SNP being associated with at least 1 (omics) trait in *G* groups of studies is given by:

$$P=1-\prod\limits_{g=1}^{G}\Pr(\text{the SNP is not associated with any trait in group \textit{g}}), $$

where the probability of the SNP being not associated with any trait in group *g* can be calculated by separately applying Primo to the low-dimensional *J*_*g*_ set of statistics within the *g*th group.

When jointly analyzing unbalanced numbers of summary statistics of different data types (e.g., 10 sets of eQTL and 1 set of pQTL statistics), caution should be taken as the joint association results can be dominated by one data type (here, eQTL), which is not ideal. One may first collapse those *J* sets of statistics by data types and apply Primo in a hierarchical fashion to the (converted) summary statistics from multiple data types. This direction will be explored in future work.

### The connection of Primo to “colocalization” and meta-analysis methods

The Primo method shares some similarities with colocalization methods, as well as meta-analysis methods. Similar to colocalization methods [15–18], Primo aims to integrate GWAS summary statistics with omics QTL statistics to provide molecular mechanistic interpretations of known trait-associated SNPs. Existing colocalization methods [15–18] are designed to study GWAS statistics with a limited number of sets of omics QTL statistics at a time, and that limits the potential of the methods given the rich availability of omics QTL statistics from different cellular contexts and studies. Additionally, the “coloc” [15] and “moloc” [16] methods assume that there is only up to one true causal variant in a region. However, the lead SNPs associated with expression levels in a gene can be different in different tissue types and cell types [6], and the SNPs for different omics QTLs may or may not share a same causal variant [21]. Motivated by those facts, Primo integrates GWAS statistics with omics and multi-omics QTL association statistics and conducts conditional association analysis in gene regions harboring known trait-associated SNPs to assess their omics trait associations accounting for LD with other lead SNPs for omics traits in the same gene regions.

Additionally, Primo enjoys a few advantages that are not shared with existing methods: Primo can integrate multiple sets of summary statistics, while also allowing some of those statistics to be from studies with correlated/overlapping samples; Primo requires only a total of *J* pre-specified parameters, $\theta _{j}^{1}$’s (which often can be estimated from the data or based on a priori knowledge), and the results are not sensitive to under-specification of those parameters; and Primo estimates the *π*_*k*_’s based on the data and separates the biological correlations/co-occurrences from sample correlations, i.e., it allows studies to be correlated. Additionally, Primo provides FDR estimates to guide the data-dependent choices of posterior probability cutoffs.

In comparison with meta-analysis, as a general association method, Primo is more flexible in accounting for study heterogeneity, allowing different GWAS and omics QTL studies to have different effect sizes even in different directions. Note that if the same directions of effect sizes are expected for a biological reason, one can also use the one-sided *P* values as input in Primo. Primo does not require the samples to be independent among different studies and can take summary statistics calculated from studies with independent, correlated, and/or overlapping samples. More importantly, in addition to the omnibus test in identifying associations in at least one study, Primo can identify SNPs in different combinations of association patterns, many of which may have biological interpretations.

Primo is a flexible integrative association method with only summary statistics used as input. It makes minimal assumptions about the data structure underlying different sets of summary statistics and assesses the joint associations across a moderate to large number of traits/data types/conditions/studies.

## Supplementary information

**Additional file 1** Supplemental methods, supplementary Table 1 and supplementary figures.

**Additional file 2** Review history.

## Data Availability

The R package Primo is freely available at https://github.com/kjgleason/Primo[60] and released under GPL-3 license. The version used in this manuscript is available under 10.5281/zenodo.3555533[61]. GTEx (V8) cis-eQTL summary statistics can be downloaded through the GTEx portal (https://www.gtexportal.org). TCGA data can be queried and downloaded through the Genomic Data Commons (GDC) portal (https://portal.gdc.cancer.gov/). GWAS summary statistics can be obtained for BCAC from http://bcac.ccge.medschl.cam.ac.uk, the GIANT consortium from http://www.broadinstitute.org/collaboration/giant, the International Inflammatory Bowel Disease Genetics Consortium from https://www.ibdgenetics.org, and the UK Biobank at http://www.nealelab.is/uk-biobank. Analysis code is available at 10.5281/zenodo.3533190[62].
