# Supplementary material for: Primo: integration of multiple GWAS and omics QTL summary statistics for elucidation of molecular mechanisms of trait-associated SNPs and detection of pleiotropy in complex traits
Source: Genome Biol. 2020 Sep 11;21:236. doi: 10.1186/s13059-020-02125-w (PMC7488447; doi:10.1186/s13059-020-02125-w)
Supplement: Supplementary file 1 — Additional file 1 Supplemental methods, supplementary Table 1 and supplementary figures. [file 13059_2020_2125_MOESM1_ESM.pdf]

METHOD

Supplemental Materials for "Primo: integration of multiple GWAS and omics QTL summary statistics for elucidation of molecular mechanisms of trait-associated SNPs and detection of pleiotropy in complex traits"

Kevin J Gleason[1†], Fan Yang[2†], Brandon L Pierce[1,3], Xin He[3] and Lin S Chen[1*]

[*]Correspondence:
lchen@health.bsd.uchicago.edu
[1]Department of Public Health Sciences, University of Chicago, 5841 South Maryland Ave MC2000, Chicago, IL 60637
Full list of author information is available at the end of the article
[†]Equal contributor

Supplemental Methods

Genotyping and imputation

GTEx (V8) samples underwent whole genome sequencing (WGS) at a median depth of 32x on Illumina HiSeq 2000 or Illumina HiSeq X. Additional details about the genotyping pipeline and sample and variant quality control have been reported elsewhere [1].

Genotype data and clinical covariates for TCGA breast cancer subjects were downloaded through the Genomic Data Commons (GDC) Data Portal [2]. TCGA subjects were genotyped on the Affymetrix Genome-wide Human SNP Array 6.0. Germline genotypes were measured in blood-derived DNA samples primarily. For subjects missing genotyping from blood samples, genotype measured in solid normal tissue was used as a surrogate. Restricting to bi-allelic variants on autosomes yielded 859,193 SNPs. After removing subjects with duplicate blood genotypes that did not match (i.e. labeling problems), there were 1094 subjects remaining. IMPUTE2 [3] was used to conduct genotype imputation using 1000 Genomes as the reference panel (phase3 v5) [4]. We performed 30 MCMC iterations, discarding the first 10 as burn-in, using 1 MB intervals for inference. SNPs with an imputation info score $<$ 0.3 or with a minor allele frequency (MAF) $<$ 0.01 were removed post-imputation.

Gene expression

GTEx RNA sequencing was performed using the Illumina TruSeq RNA protocol. Data was aligned using STAR (v2.5.3a) [5]. Picard [6] was used to process raw sequence data. RNA-SeQC [7] was used for quality control and gene-level expression quantification, and TMM [8] was used to normalize read counts. Additional details on the RNA-Sequencing pipeline and processing are reported elsewhere [1].

Gene expression, protein abundance, and DNA methylation data for TCGA subjects were downloaded using TCGA-Assembler 2 [9]. RNA sequencing was performed in tumor tissues using the Illumina HiSeq 2000 RNA Sequencing platform. RNA-Seq expression levels were quantified using HTSeq-count [10]. Expression data were quantile normalized prior to analysis.

DNA methylation

DNA methylation was measured in tumor tissue samples using the Infinium HumanMethylation450 BeadChip. The level of methylation at each CpG site was measured as a β value ranging from 0 (completely unmethylated) to 1 (completely methylated). Data were quantile normalized prior to analysis.

Protein abundance

Protein abundance was measured in tumor tissue samples using iTRAQ (isobaric tag for relative and absolute quantitation) mass-spectometry (MS) in experiments conducted by the Clinical Proteomic Tumor Analysis Consortium (CPTAC) [11]. The protein abundance was measured using the Log Ratio (i.e. the log of the ratio between the spectral count of a protein in a sample versus the spectral count of the protein in the reference sample). We restricted our analysis of protein abundance to the 77 high-quality samples as identified by Mertins, *et al.* [12]. We further restricted the analysis to the 74 of these 77 samples from female subjects with measured tumor purity scores [13].

QTL analyses

GTEx *cis*-eQTL summary statistics were generated by linear regression as implemented in FastQTL [14], adjusting for sex, genotyping platform, WGS library construction protocol (PCR-based or PCR-free), five genotype principal components (PCs), and up to 60 PEER [15] variables.

Prior to QTL analyses, expression, methylation and protein measurements for TCGA Samples were transformed to the quantiles of the standard normal distribution (separately for each gene, CpG site or protein). Imputed genotypes were converted to expected counts of the alternative allele. We generated *cis*-QTL summary statistics using linear regression as implemented in Matrix eQTL [16], adjusting for subject covariates. QTL analyses were restricted to female subjects. Covariates included tumor purity scores [13], cancer stage, histological subtype (infiltrating ductal, infiltrating lobular, mucinous, metaplastic, mixed histology or other), estrogen receptor (ER) and progesterone receptor (PR) status, and genotype principal components (15 PCs for expression and methylation; 3 PCs for protein). Genotype PCs were generated in PLINK 1.90 [17, 18] using measured bi-allelic variants on autosomes with the following filters: minor allele frequency ≥ 0.05; Hardy-Weinberg Equilibrium p-value ≥ 0.0001; pairwise linkage disequilibrium $R^2 \leq 0.2$.

Cis associations were defined as: $<$ 250 kb apart from transcription start site (TSS) for expression and protein; $<$ 50 kb apart from a CpG site for methylation. For GWAS-reported SNPs that were missing based on these criteria, we selected the nearest gene (based on distance between the SNP and transcription start site) for which both gene expression and protein abundance levels were available (Application I in the main text) or for which gene expression levels were measured in both tissues (Application II in the main text). For these regions, we included associations $<$ 1 Mb apart from the transcription start site. Note that using different definitions of cis window sizes may have yielded slight differences in results. For genes in regions of GWAS-reported SNPs missing protein or methylation data, and for GWAS-reported SNPs that could not be mapped to a GTEx variant, a t-statistic of 0 was added for the missing data to allow integration of the non-missing test-statistics.

Annotation of CpG sites

For the enrichment analysis of CpG targets of breast susceptibility loci, we performed annotation using publicly available datasets. Exons and introns were annotated using the refGene database provided through the UCSC Table Browser [19]. Promoter regions for genes were defined as the regions 0–1500 bases upstream of the TSS. Enhancer regions were annotated using H3K4me1 and H3K27ac histone marks in human mammary epithelial and breast myoepithelial cells using data from the Roadmap Epigenomics Project [20] and the Encyclopedia of DNA Elements Project (ENCODE) [21]. We downloaded the call sets from the ENCODE portal (https://www.encodeproject.org/) [22] with the following identifiers: ENCFF001SWW, ENCFF001SWZ. To test for enrichment of features among the CpG targets, we used 10,000 bootstrapped samples of all CpG sites on the 450k array and calculated P-values using the proportion of bootstrapped samples with more extreme counts than were observed in the CpG targets identified by Primo.

Supplemental Results

Simulation of π_k estimation for mis-specification of alternative proportions

We evaluated the performance of estimating the proportion of SNPs belonging to each association pattern (π_k) when the marginal alternative proportions θ_j^1's are mis-specified. Results are shown in Table S1. Scenario S1a simulates sparse associations, with true ($\pi_k = 7 \times 10^{-4}$, 2×10^{-4}, 1×10^{-4}) for SNPs being associated with only one, exactly two, and all three traits, respectively. Scenario S1b simulated even sparser associations for the third trait, with $\pi_k = (7 \times 10^{-6}, 2 \times 10^{-6}, 1 \times 10^{-6})$ for SNPs being associated with only the third, the third and first or second, and all three traits, respectively. The θ_j^1's are under-specified as $\theta_j^1/10$ and over-specified as $\theta_j^1 \times 10$. Primo estimates the π_k's with reasonable accuracy even when the associations are very sparse and when the marginal alternative proportions θ_j^1's are under-specified.

Numerical optimization simulation for P-values

We evaluated the performance of Primo in estimating the scaling factor (A_j) and degrees of freedom (d'_j) parameters of the alternative distribution for $t_{ij} = -2\log(p_{ij})$ $(1, \ldots, m)$. Under different specifications of the proportion of statistics coming from the alternative distribution (θ_j^1), we simulated 10 million test statistics. Test statistics under the null hypothesis of no association were simulated from a χ_2^2 distribution; test statistics under the alternative were simulated from a $A_j\chi^2_{d'_j}$ distribution. Fig. S1 compares the density curves of the true alternative density to the alternative densities estimated by Primo over 1000 simulations for $A_j = 4.5$ and $d'_j = 7$. As shown, the density curves estimated by Primo reasonably approximate the true density curve even when the proportion of statistics coming from the alternative distribution is sparse ($\theta_j^1 = 1 \times 10^{-4}$; panel S1A) or very sparse ($\theta_j^1 = 1 \times 10^{-5}$; panel S1B).

Detecting SNPs with pleiotropic effects on Crohn's disease and ulcerative colitis and elucidating their mechanisms

Here we applied Primo to integrate GWAS summary statistics of Crohn's disease and ulcerative colitis from a study of over 20,000 samples of European Ancestry

Scenario	Specific.	Method	$\pi_k(\%)$							
			q_k=(0 0 0)	(1 0 0)	(0 1 0)	(0 0 1)	(1 1 0)	(1 0 1)	(0 1 1)	(1 1 1)
S1a			Independent							
		True	99.720	0.070	0.070	0.070	0.020	0.020	0.020	0.010
	Under	Primo (t)	99.746	0.065	0.065	0.065	0.017	0.017	0.017	0.008
		Primo (P)	99.856	0.042	0.042	0.042	0.006	0.006	0.006	0.001
	Over	Primo (t)	98.991	0.262	0.263	0.262	0.057	0.057	0.057	0.051
		Primo (P)	95.944	0.967	0.967	0.966	0.320	0.320	0.320	0.196
			Correlated							
		True	99.720	0.070	0.070	0.070	0.020	0.020	0.020	0.010
	Under	Primo (t)	99.746	0.065	0.065	0.065	0.017	0.017	0.017	0.008
		Primo (P)	99.856	0.042	0.042	0.042	0.006	0.006	0.006	0.001
	Over	Primo (t)	99.024	0.247	0.248	0.247	0.058	0.058	0.058	0.059
		Primo (P)	95.894	0.928	0.930	0.929	0.359	0.360	0.360	0.241
S1b			Independent							
		True	99.839	0.070	0.070	0.0007	0.020	0.0002	0.0002	0.0001
	Under	Primo (t)	99.854	0.064	0.064	0.0018	0.016	0.0002	0.0002	0.0001
		Primo (P)	99.922	0.036	0.036	0.0004	0.005	0.0001	0.0001	< 0.0001
	Over	Primo (t)	99.317	0.282	0.282	0.0447	0.068	0.0030	0.0030	0.0007
		Primo (P)	97.260	1.117	1.116	0.0788	0.389	0.0175	0.0175	0.0047
			Correlated							
		True	99.839	0.070	0.070	0.0007	0.020	0.0002	0.0002	0.0001
	Under	Primo (t)	99.854	0.064	0.064	0.0017	0.016	0.0002	0.0002	0.0001
		Primo (P)	99.922	0.036	0.036	0.0004	0.005	0.0001	0.0001	< 0.0001
	Over	Primo (t)	99.340	0.269	0.270	0.0410	0.071	0.0038	0.0038	0.0021
		Primo (P)	97.267	1.079	1.081	0.0630	0.439	0.0267	0.0266	0.0181

Table S1: Average estimates of π_k's with mis-specification of the alternative proportions, θ_j^1's. In the simulations, we used $\theta_j^1/10$ when under-specifying the parameters and used $\theta_j^1 \times 10$ when over-specifying them. Scenario S1a simulates sparse associations for $J = 3$ traits. Scenario S1b simulates even sparser associations for the third trait. All numbers presented in the table are percentages. When θ_j^1's are over-specified, $\hat{\pi}_k$'s deviate from true π_k's. When θ_j^1's are under-specified, $\hat{\pi}_k$'s are close to true π_k's.

conducted by the International Inflammatory Bowel Disease (IBD) Genetics Consortium [23] with eQTL summary statistics from sigmoid colon (n = 318) and transverse colon (n = 368) tissues from GTEx. Of the 232 SNPs reported in the initial meta-analysis, 67 SNPs have reached genome-wide significance for at least one of Crohn's disease or ulcerative colitis and could be mapped to GTEx SNPs in cis with at least one gene measured in each tissue. At the 80% probability cutoff (estimated FDR of 0.8%, 5.8% and 6.4%) and after conditional association analysis accounting for LD, 37, 15 and 11 of the 67 SNPs were associated with both complex traits, both complex traits plus gene expression in at least 1 tissue, and both complex traits plus gene expression in both tissues, respectively. We used GWAS summary statistics of self-reported Crohn's disease and self-reported ulcerative colitis from the UK Biobank [24] to replicate our findings. At $P < 0.0007$ (0.05/(37 $\times$ 2)), 4 of the 37 SNPs were associated with both traits in the UK Biobank. The relatively low replication rate might be due to the fact that in the UK Biobank data, self-reported disease status was used for both traits and the numbers of cases of Crohn's disease and ulcerative colitis are low (< 2000 and < 3000, respectively).

Reference

Author details
[1]Department of Public Health Sciences, University of Chicago, 5841 South Maryland Ave MC2000, Chicago, IL 60637. [2]Department of Biostatistics and Informatics, Colorado School of Public Health, University of Colorado Denver, 13001 E. 17th Place, Aurora, Colorado 80045. [3]Department of Human Genetics, University of Chicago, 920 E 58th St, Chicago, IL 60637.

References

1. Aguet F, Barbeira AN, Bonazzola R, Brown A, Castel SE, Jo B, et al. The GTEx Consortium atlas of genetic regulatory effects across human tissues. bioRxiv. 2019;doi: 10.1101/787903.
2. Grossman RL, Heath AP, Ferretti V, Varmus HE, Lowy DR, Kibbe WA, et al. Toward a Shared Vision for Cancer Genomic Data. N Engl J Med. 2016 Sep;375(12):1109–1112.
3. Howie BN, Donnelly P, Marchini J. A flexible and accurate genotype imputation method for the next generation of genome-wide association studies. PLoS Genet. 2009 Jun;5(6):e1000529.
4. Auton A, Brooks LD, Durbin RM, Garrison EP, Kang HM, Korbel JO, et al. A global reference for human genetic variation. Nature. 2015 Oct;526(7571):68–74.
5. Dobin A, Davis CA, Schlesinger F, Drenkow J, Zaleski C, Jha S, et al. STAR: ultrafast universal RNA-seq aligner. Bioinformatics. 2013 Jan;29(1):15–21.
6. Broad Institute. Picard Tools; 2019. Available from: `http://broadinstitute.github.io/picard/`.
7. DeLuca DS, Levin JZ, Sivachenko A, Fennell T, Nazaire MD, Williams C, et al. RNA-SeQC: RNA-seq metrics for quality control and process optimization. Bioinformatics. 2012 Jun;28(11):1530–1532.
8. Robinson MD, Oshlack A. A scaling normalization method for differential expression analysis of RNA-seq data. Genome Biol. 2010;11(3):R25.
9. Wei L, Jin Z, Yang S, Xu Y, Zhu Y, Ji Y. TCGA-assembler 2: software pipeline for retrieval and processing of TCGA/CPTAC data. Bioinformatics. 2018 May;34(9):1615–1617.
10. Anders S, Pyl PT, Huber W. HTSeq–a Python framework to work with high-throughput sequencing data. Bioinformatics. 2015 Jan;31(2):166–169.
11. Ellis MJ, Gillette M, Carr SA, Paulovich AG, Smith RD, Rodland KK, et al. Connecting genomic alterations to cancer biology with proteomics: the NCI Clinical Proteomic Tumor Analysis Consortium. Cancer Discov. 2013 Oct;3(10):1108–1112.
12. Mertins P, Mani DR, Ruggles KV, Gillette MA, Clauser KR, Wang P, et al. Proteogenomics connects somatic mutations to signalling in breast cancer. Nature. 2016 06;534(7605):55–62.
13. Aran D, Sirota M, Butte AJ. Systematic pan-cancer analysis of tumour purity. Nat Commun. 2015 Dec;6:8971.
14. Ongen H, Buil A, Brown AA, Dermitzakis ET, Delaneau O. Fast and efficient QTL mapper for thousands of molecular phenotypes. Bioinformatics. 2016 05;32(10):1479–1485.
15. Stegle O, Parts L, Piipari M, Winn J, Durbin R. Using probabilistic estimation of expression residuals (PEER) to obtain increased power and interpretability of gene expression analyses. Nat Protoc. 2012 Feb;7(3):500–507.
16. Shabalin AA. Matrix eQTL: ultra fast eQTL analysis via large matrix operations. Bioinformatics. 2012 May;28(10):1353–1358.
17. Chang CC, Chow CC, Tellier LC, Vattikuti S, Purcell SM, Lee JJ. Second-generation PLINK: rising to the challenge of larger and richer datasets. Gigascience. 2015;4:7.
18. Purcell S, Chang C. PLINK 1.90; 2017. Available from: `http://www.cog-genomics.org/plink/1.9/`.
19. Karolchik D, Hinrichs AS, Furey TS, Roskin KM, Sugnet CW, Haussler D, et al. The UCSC Table Browser data retrieval tool. Nucleic Acids Res. 2004 Jan;32(Database issue):D493–496.
20. Kundaje A, Meuleman W, Ernst J, Bilenky M, Yen A, Heravi-Moussavi A, et al. Integrative analysis of 111 reference human epigenomes. Nature. 2015 Feb;518(7539):317–330.
21. Dunham I, Kundaje A, Aldred SF, Collins PJ, Davis CA, Doyle F, et al. An integrated encyclopedia of DNA elements in the human genome. Nature. 2012 Sep;489(7414):57–74.
22. Davis CA, Hitz BC, Sloan CA, Chan ET, Davidson JM, Gabdank I, et al. The Encyclopedia of DNA elements (ENCODE): data portal update. Nucleic Acids Res. 2018 01;46(D1):D794–D801.
23. Liu JZ, van Sommeren S, Huang H, Ng SC, Alberts R, Takahashi A, et al. Association analyses identify 38 susceptibility loci for inflammatory bowel disease and highlight shared genetic risk across populations. Nat Genet. 2015 Sep;47(9):979–986.
24. Churchhouse C, Neale B. Rapid GWAS of thousands of phenotypes for 337,000 samples in the UK Biobank; 2017. Available from: `http://www.nealelab.is/blog/2017/7/19/rapid-gwas-of-thousands-of-phenotypes-for-337000-samples-in-the-uk-biobank`.

Supplementary figures

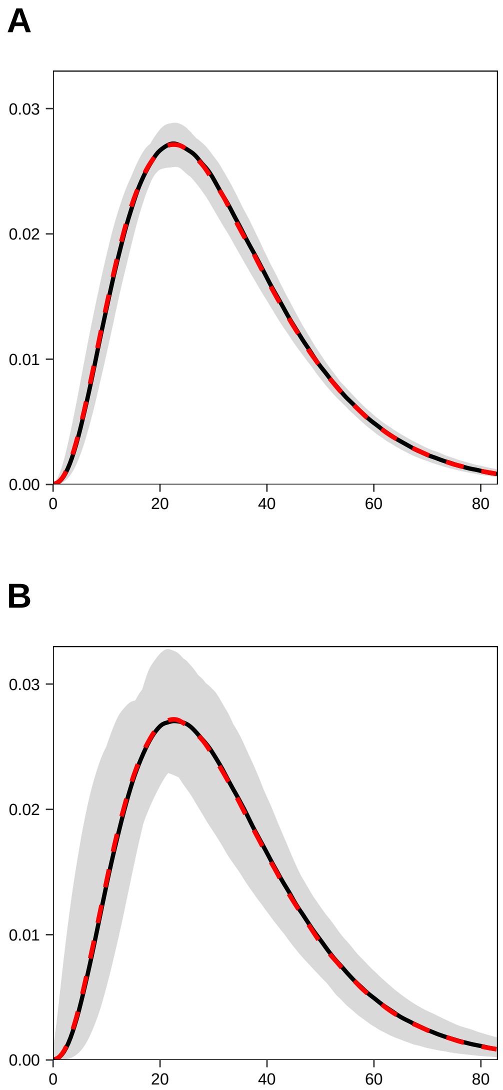

Fig. S1: Primo reasonably estimates the scaling factor (A_j) and degrees of freedom (d'_j) for the alternative distribution, even when associations are sparse. For $A_j = 4.5$ and $d'_j = 7$, the true density curve is shown by the dotted red curve in A and B. For $\theta_j^1 = 1 \times 10^{-4}$ (A) and $\theta_j^1 = 1 \times 10^{-5}$ (B), the density curves estimated by the median estimates of the parameters over 1000 simulations are given by the black curve. The shaded gray area shows the curves of the parameters between the $5th$ and $95th$ percentiles.

Fig. S2: Locus-zoom plots of $-\log_{10}(P)$-values for associations with Height, BMI (from GWAS) and gene expression levels (from GTEx eQTL analysis) for gene regions with pleiotropic SNPs being replicated. Each page shows a set of four association plots for a locus: one for Height, one for BMI, and one for gene expression in each of the two tissue types – subcutaneous adipose and skeletal muscle. In each plot, the GWAS-reported SNP is colored red if associated with the given trait and colored blue if not associated with the given trait (at an 80% posterior probability threshold and after conditional association analysis accounting for LD). Lead omics SNPs which were adjusted for in conditional association analysis are shown by a green "X". For any SNP which was associated with expression of multiple genes, the gene with which it has the strongest association is presented in each tissue.

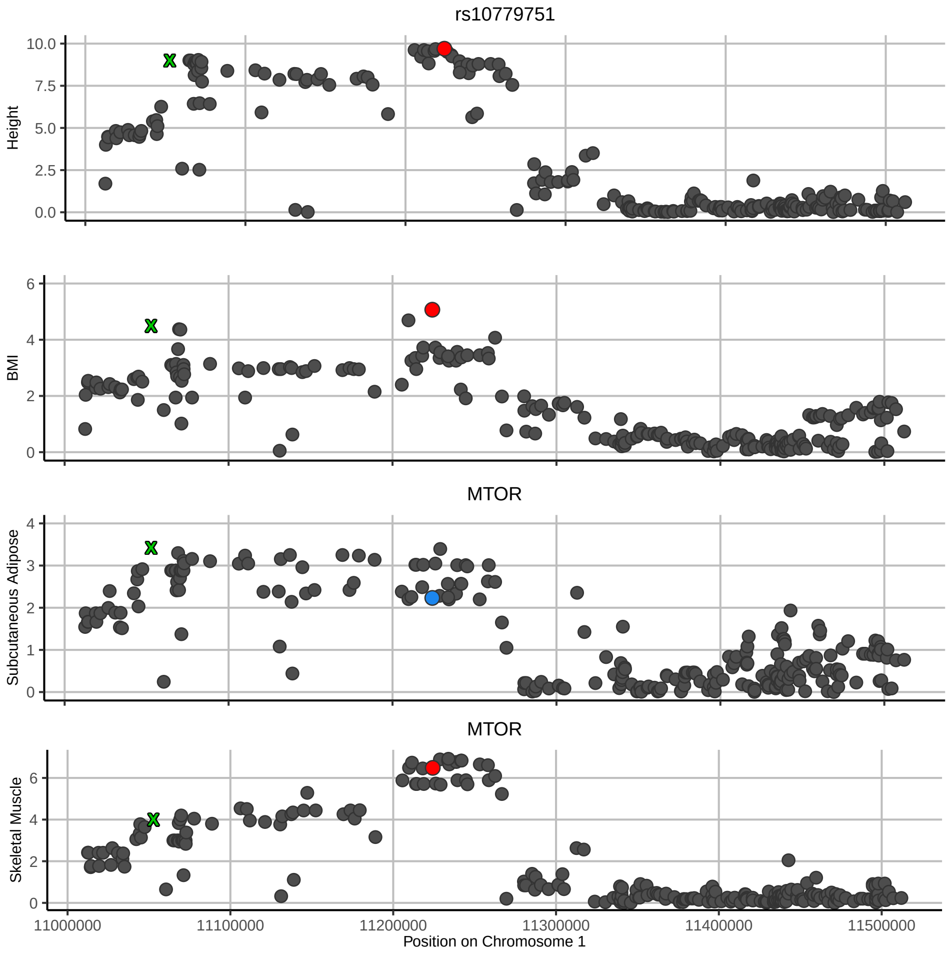

rs10779751
Height
BMI
MTOR
Subcutaneous Adipose
MTOR
Skeletal Muscle
11000000
11100000
11200000
11300000
11400000
11500000
Position on Chromosome 1

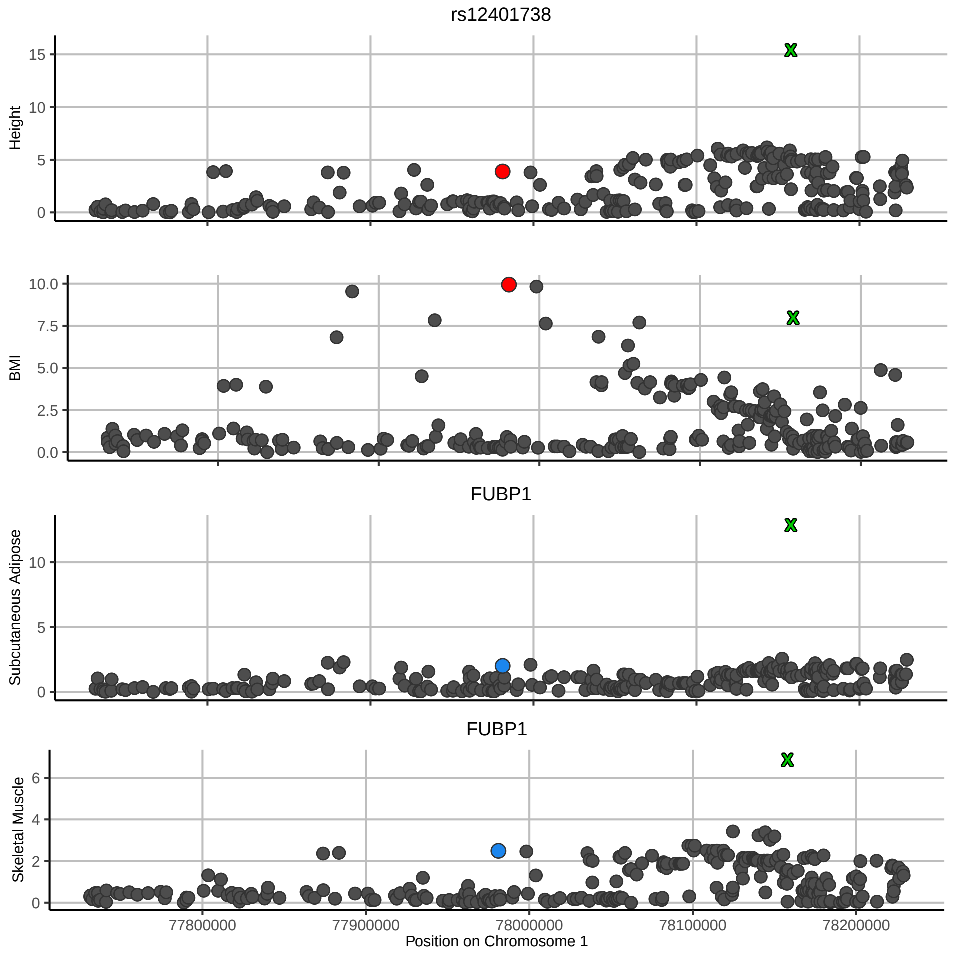
rs12401738
Height
BMI
FUBP1
Subcutaneous Adipose
FUBP1
Skeletal Muscle
77800000
77900000
78000000
78100000
78200000
Position on Chromosome 1

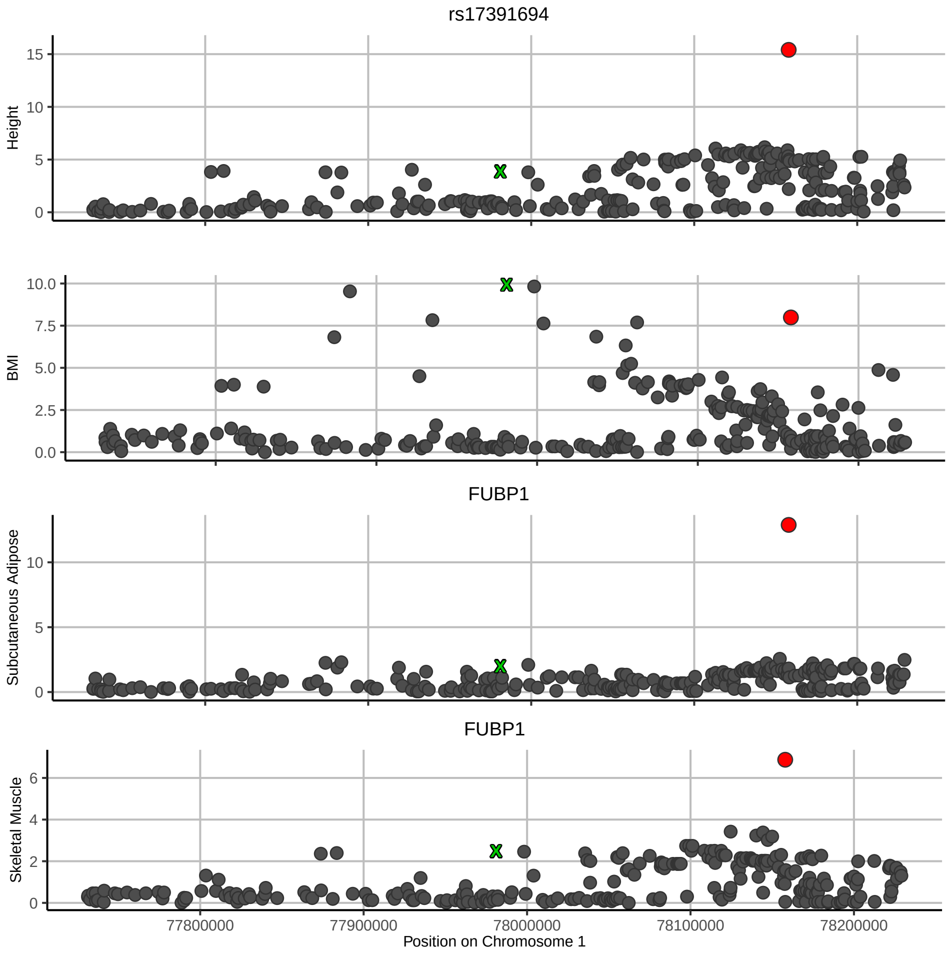

rs17391694
Height
15
10
5
0
BMI
10.0
7.5
5.0
2.5
0.0
FUBP1
Subcutaneous Adipose
10
5
0
FUBP1
Skeletal Muscle
6
4
2
0
77800000
77900000
78000000
78100000
78200000
Position on Chromosome 1

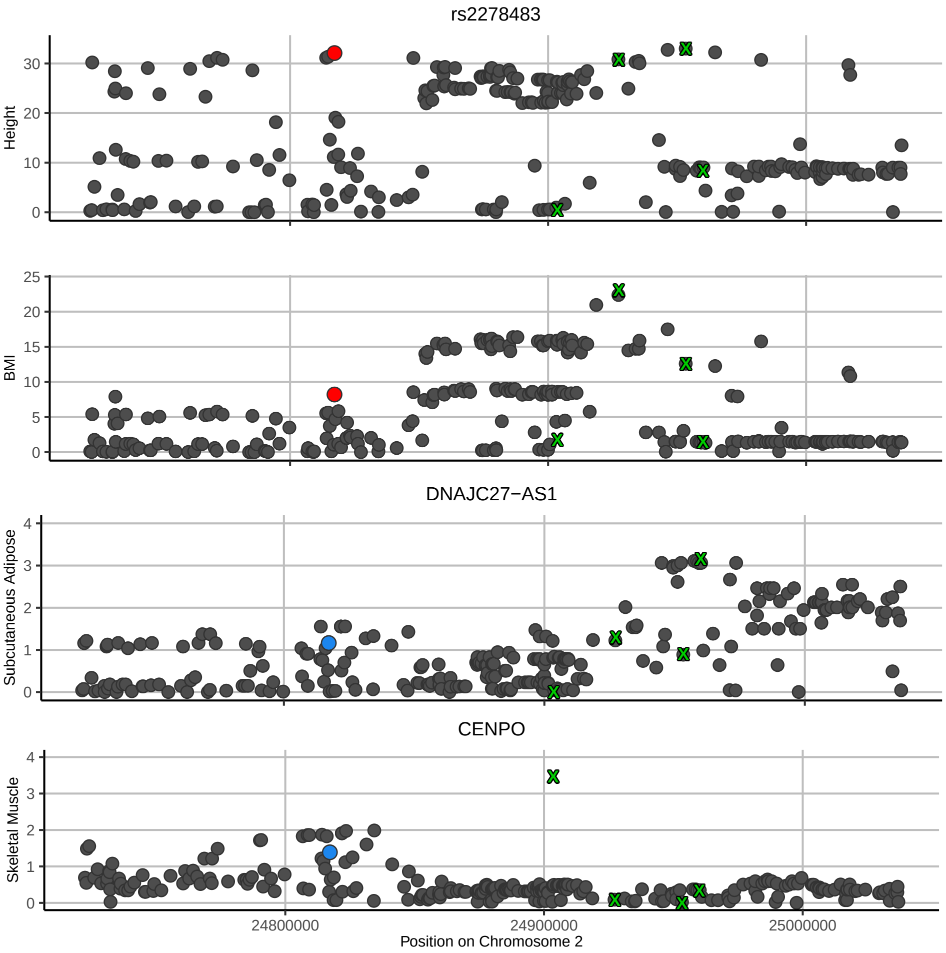

rs2278483
Height
BMI
DNAJC27−AS1
Subcutaneous Adipose
CENPO
Skeletal Muscle
24800000
24900000
25000000
Position on Chromosome 2

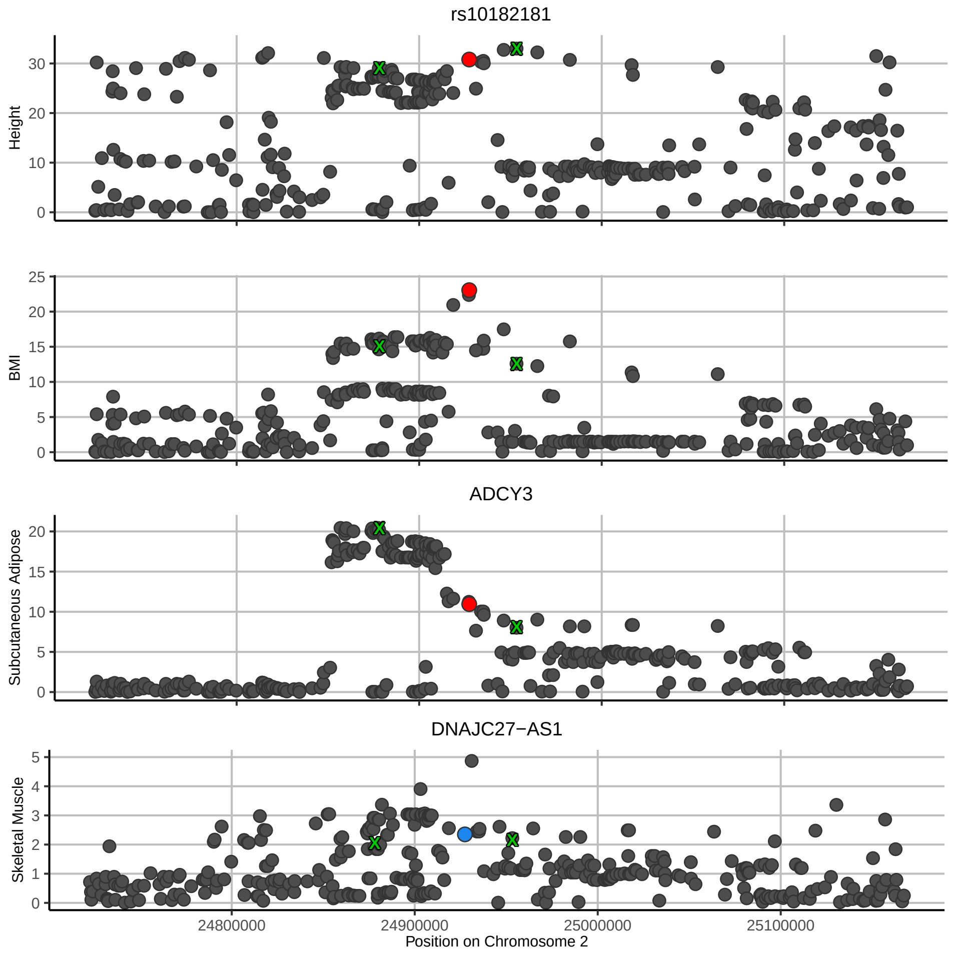

rs10182181
Height
BMI
ADCY3
Subcutaneous Adipose
DNAJC27−AS1
Skeletal Muscle
24800000
24900000
25000000
25100000
Position on Chromosome 2

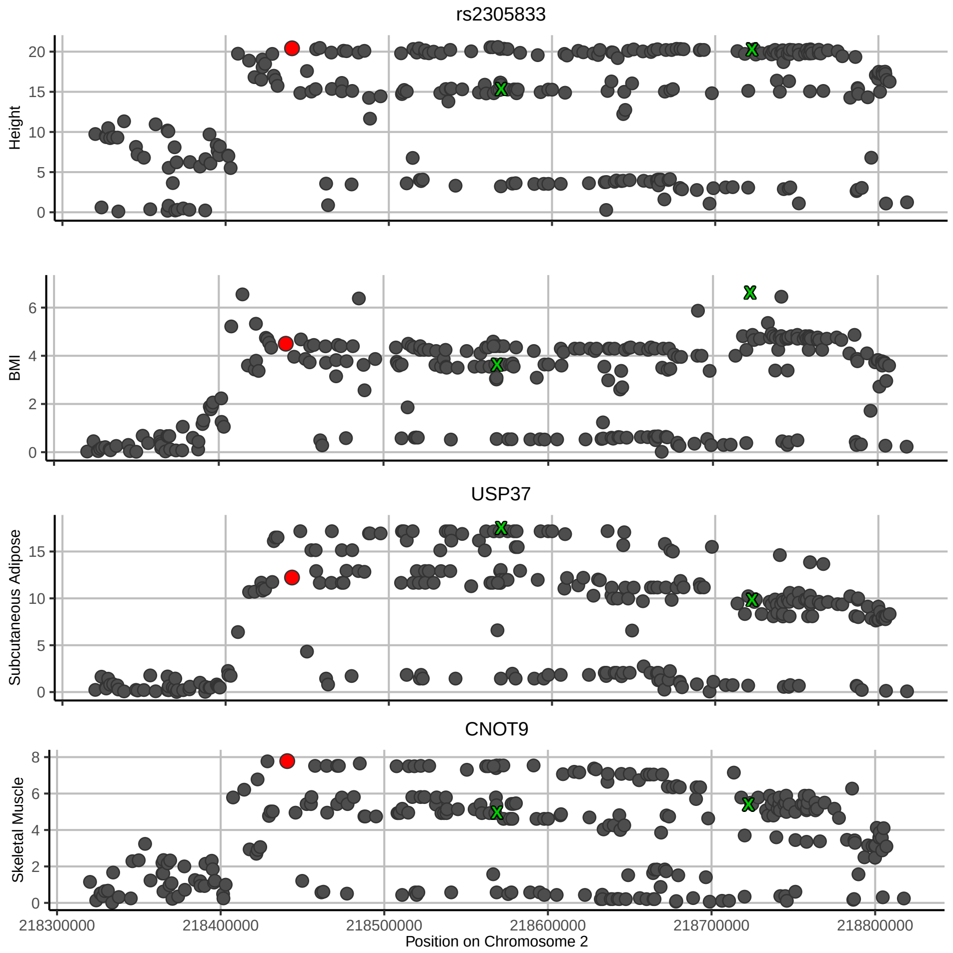
rs2305833
Height
BMI
USP37
Subcutaneous Adipose
CNOT9
Skeletal Muscle
218300000
218400000
218500000
218600000
218700000
218800000
Position on Chromosome 2

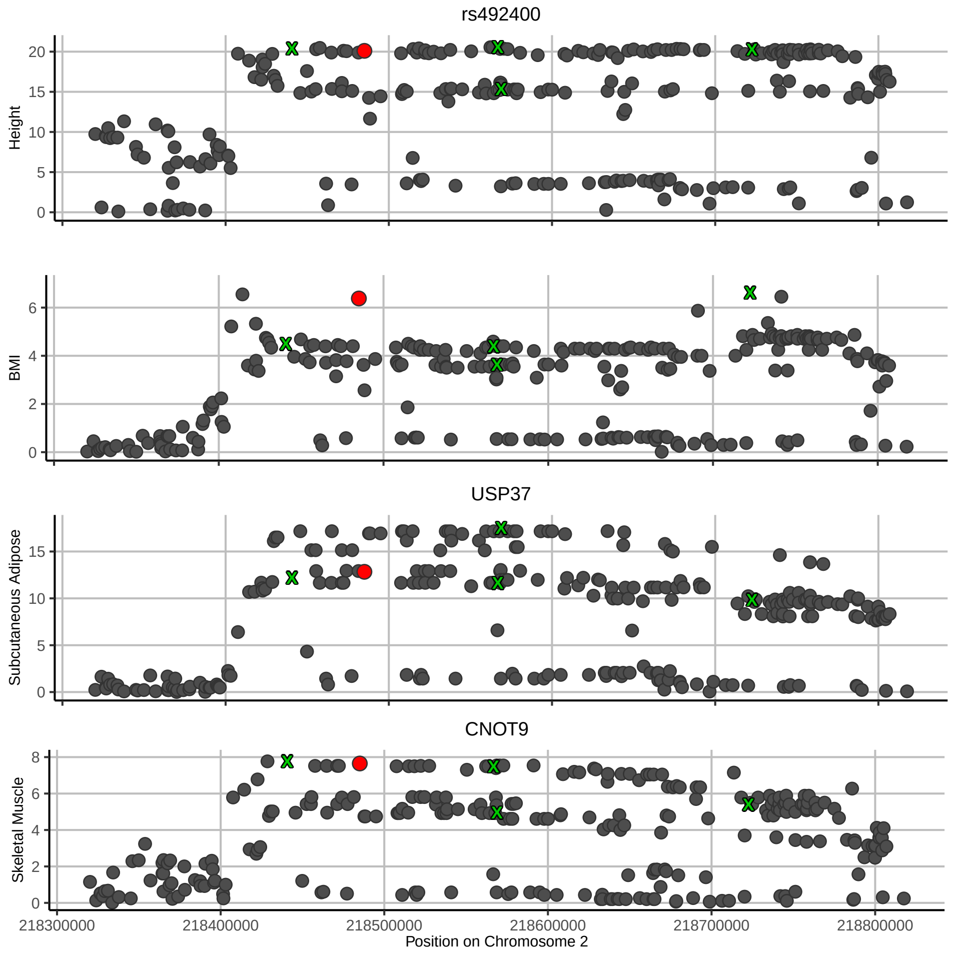

rs492400
Height
BMI
USP37
Subcutaneous Adipose
CNOT9
Skeletal Muscle
218300000
218400000
218500000
218600000
218700000
218800000
Position on Chromosome 2

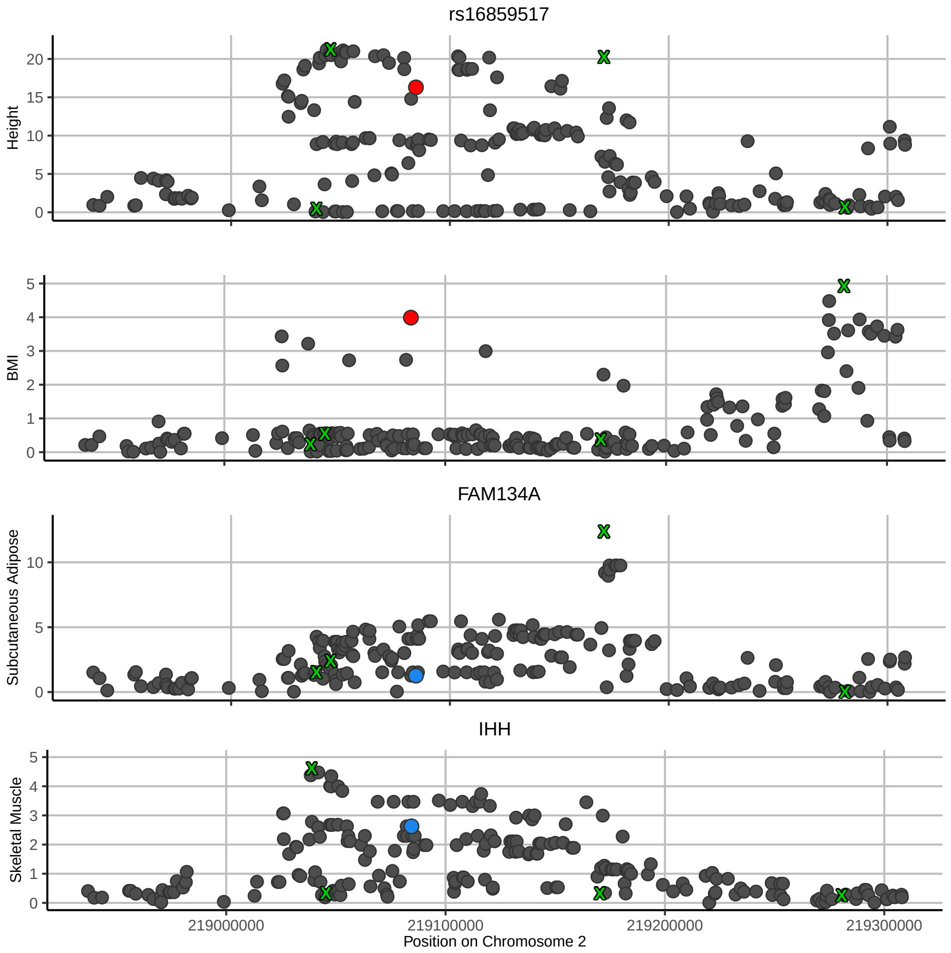

rs16859517
Height
BMI
FAM134A
Subcutaneous Adipose
IHH
Skeletal Muscle
219000000
219100000
219200000
219300000
Position on Chromosome 2

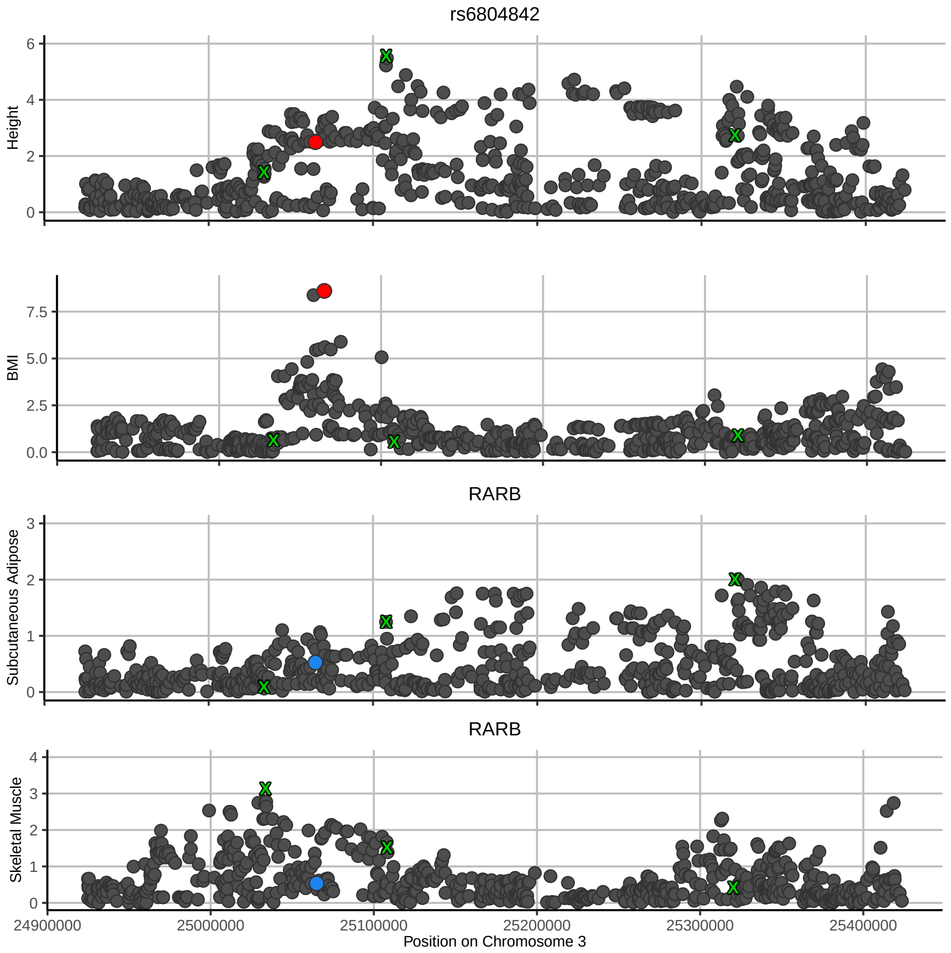

rs6804842
Height
6
4
2
0
BMI
7.5
5.0
2.5
0.0
RARB
Subcutaneous Adipose
3
2
1
0
RARB
Skeletal Muscle
4
3
2
1
0
24900000
25000000
25100000
25200000
25300000
25400000
Position on Chromosome 3

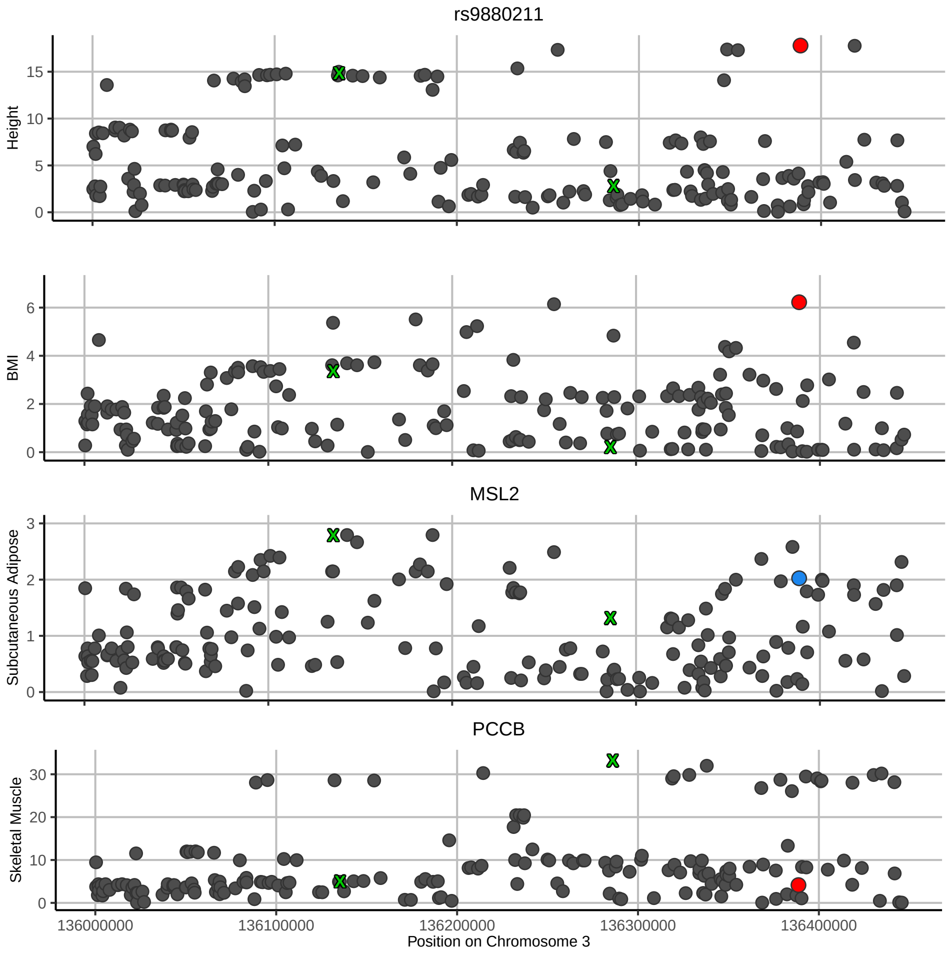

rs9880211
Height
BMI
MSL2
Subcutaneous Adipose
PCCB
Skeletal Muscle
136000000
136100000
136200000
136300000
136400000
Position on Chromosome 3

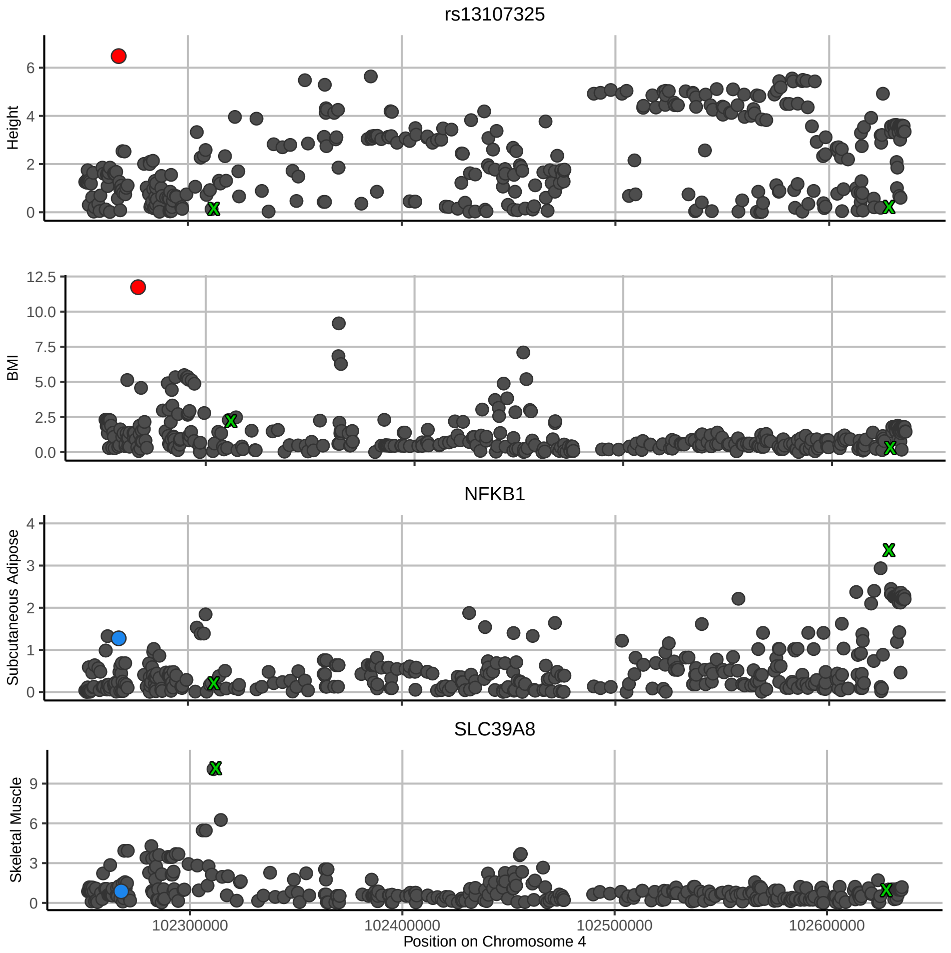

rs13107325
Height
BMI
NFKB1
Subcutaneous Adipose
SLC39A8
Skeletal Muscle
102300000
102400000
102500000
102600000
Position on Chromosome 4

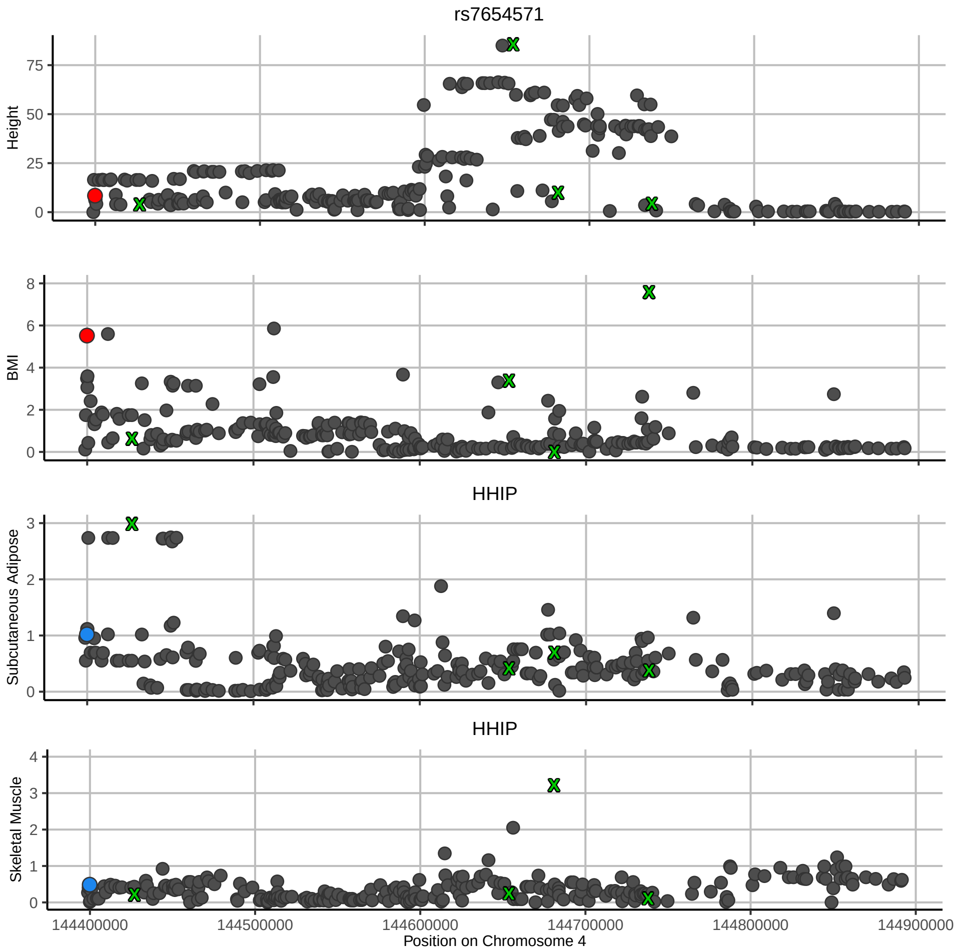
rs7654571
Height
BMI
HHIP
Subcutaneous Adipose
HHIP
Skeletal Muscle
144400000
144500000
144600000
144700000
144800000
144900000
Position on Chromosome 4

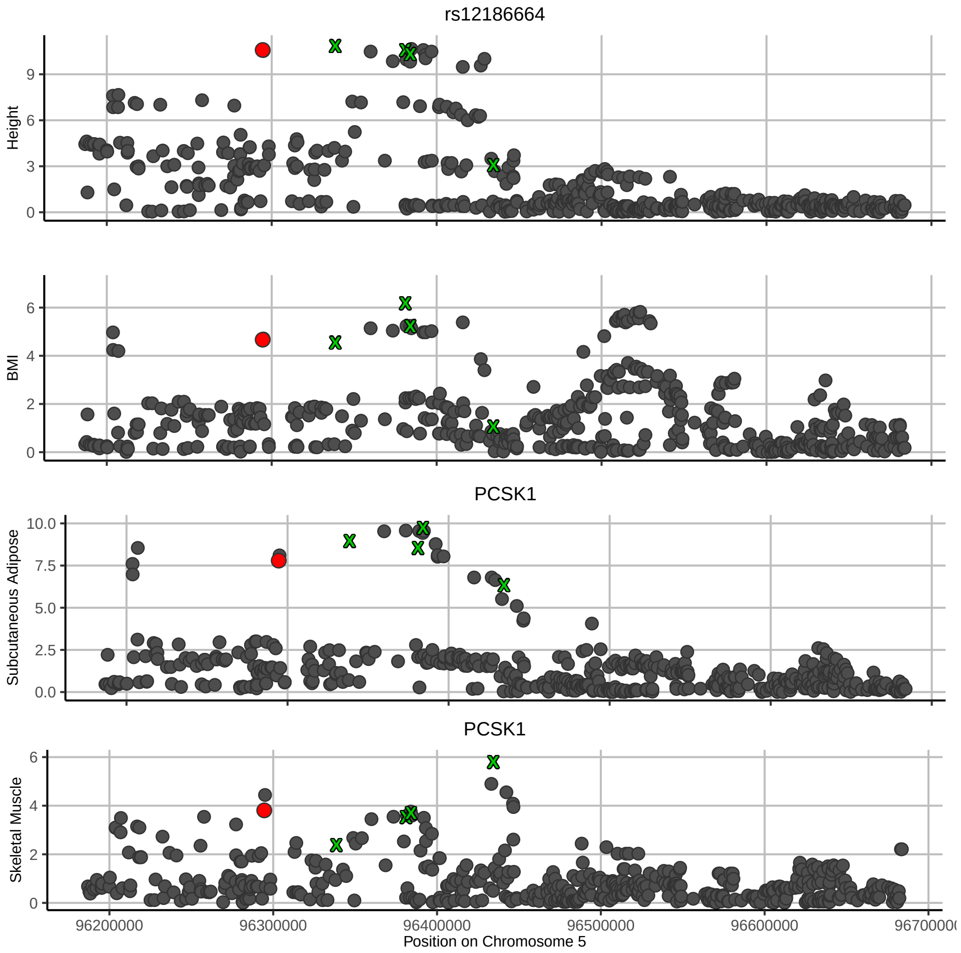

rs12186664
Height
9
6
3
0
BMI
6
4
2
0
PCSK1
Subcutaneous Adipose
10.0
7.5
5.0
2.5
0.0
PCSK1
Skeletal Muscle
6
4
2
0
96200000
96300000
96400000
96500000
96600000
Position on Chromosome 5

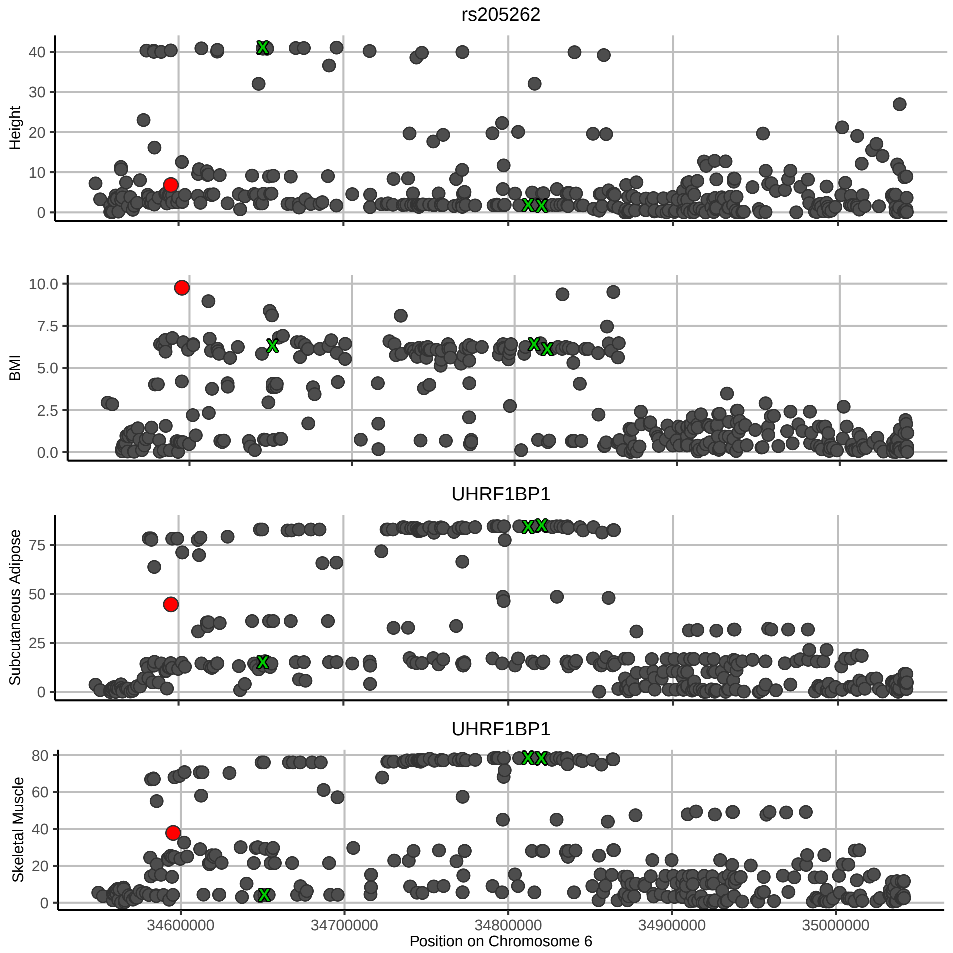

rs205262
Height
BMI
UHRF1BP1
Subcutaneous Adipose
UHRF1BP1
Skeletal Muscle
34600000
34700000
34800000
34900000
35000000
Position on Chromosome 6

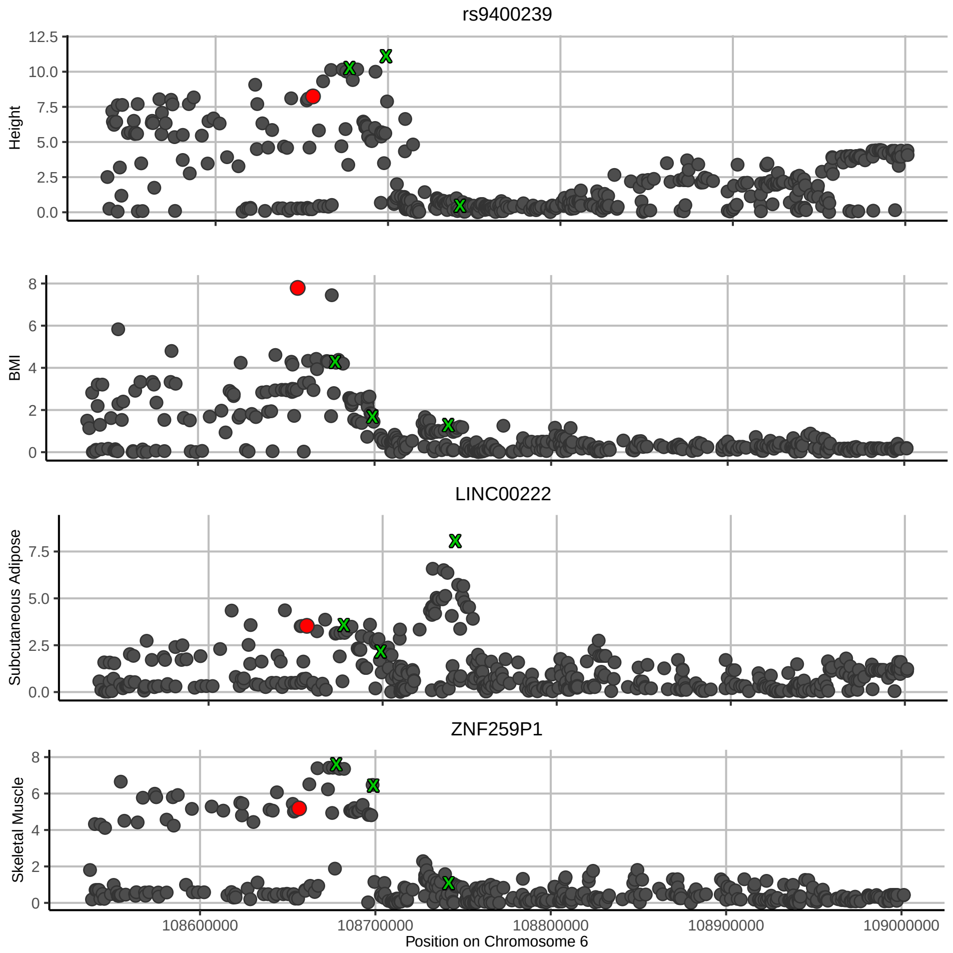

rs9400239
Height
12.5
10.0
7.5
5.0
2.5
0.0
BMI
8
6
4
2
0
LINC00222
Subcutaneous Adipose
7.5
5.0
2.5
0.0
ZNF259P1
Skeletal Muscle
8
6
4
2
0
108600000
108700000
108800000
108900000
109000000
Position on Chromosome 6

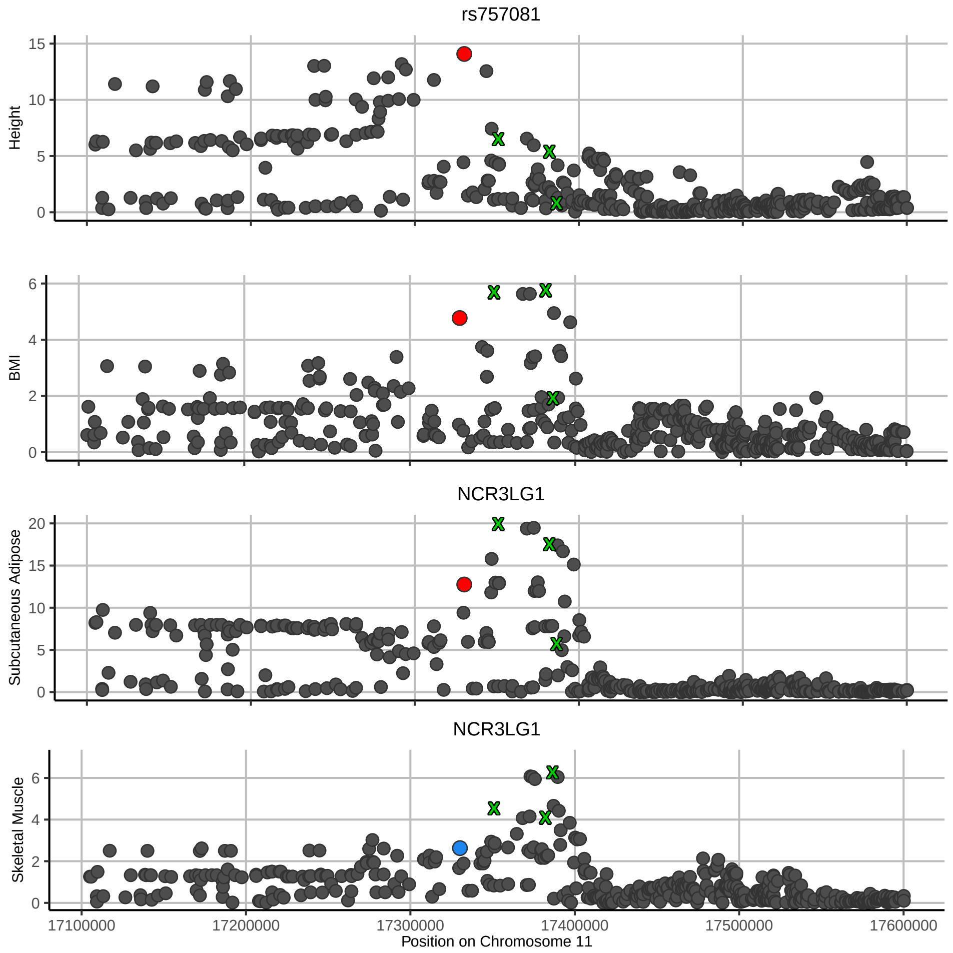

rs757081
Height
BMI
NCR3LG1
Subcutaneous Adipose
NCR3LG1
Skeletal Muscle
17100000
17200000
17300000
17400000
17500000
17600000
Position on Chromosome 11

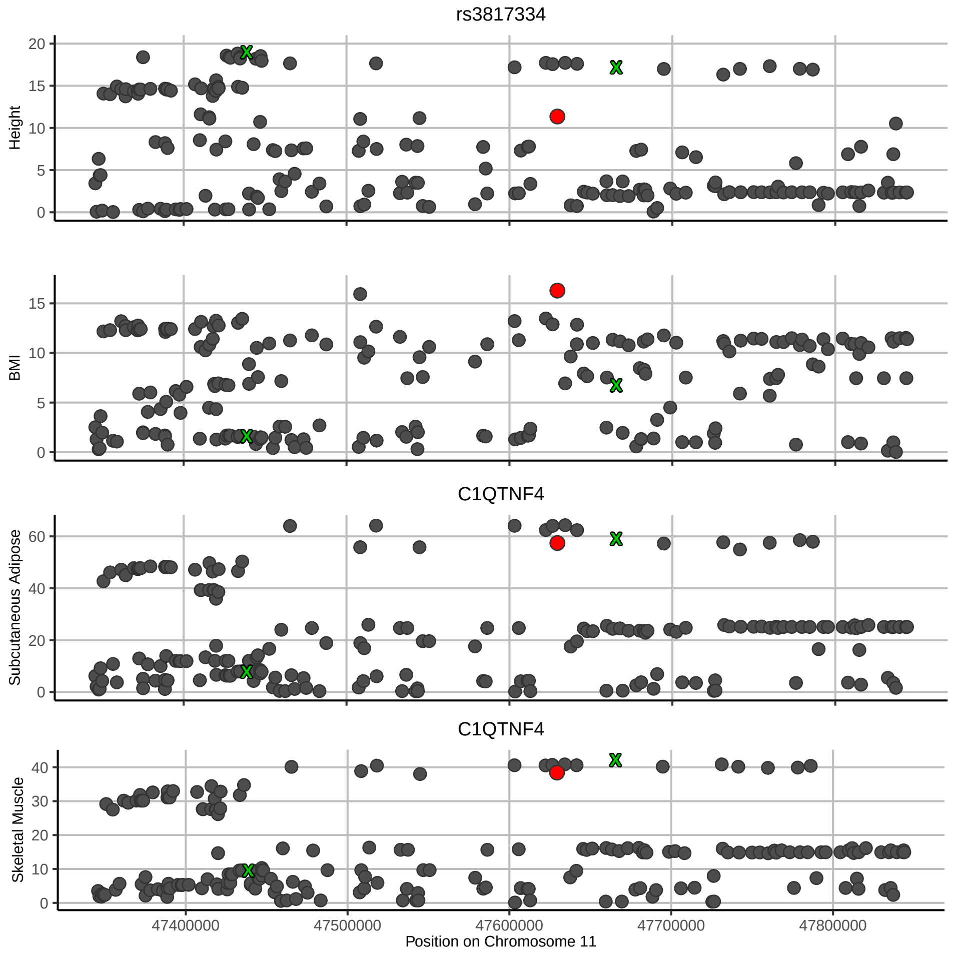
rs3817334
Height
BMI
C1QTNF4
Subcutaneous Adipose
C1QTNF4
Skeletal Muscle
47400000
47500000
47600000
47700000
47800000
Position on Chromosome 11

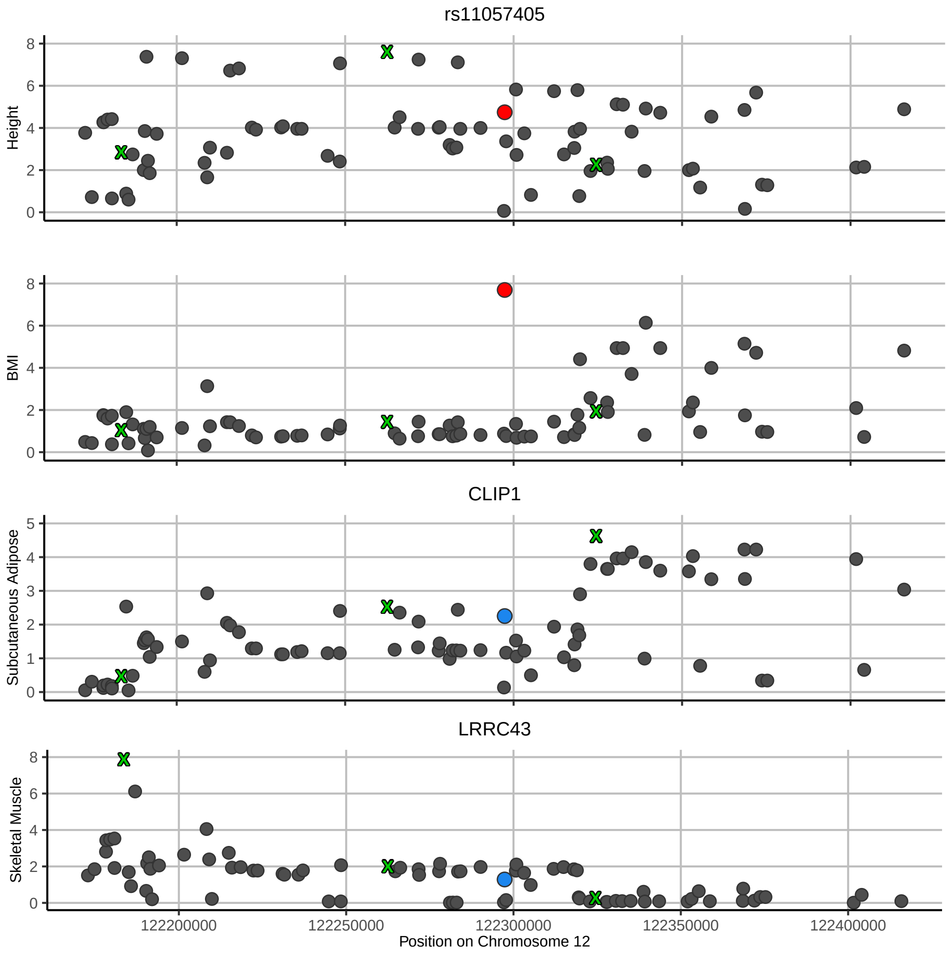

rs11057405
Height
BMI
CLIP1
Subcutaneous Adipose
LRRC43
Skeletal Muscle
122200000
122250000
122300000
122350000
122400000
Position on Chromosome 12

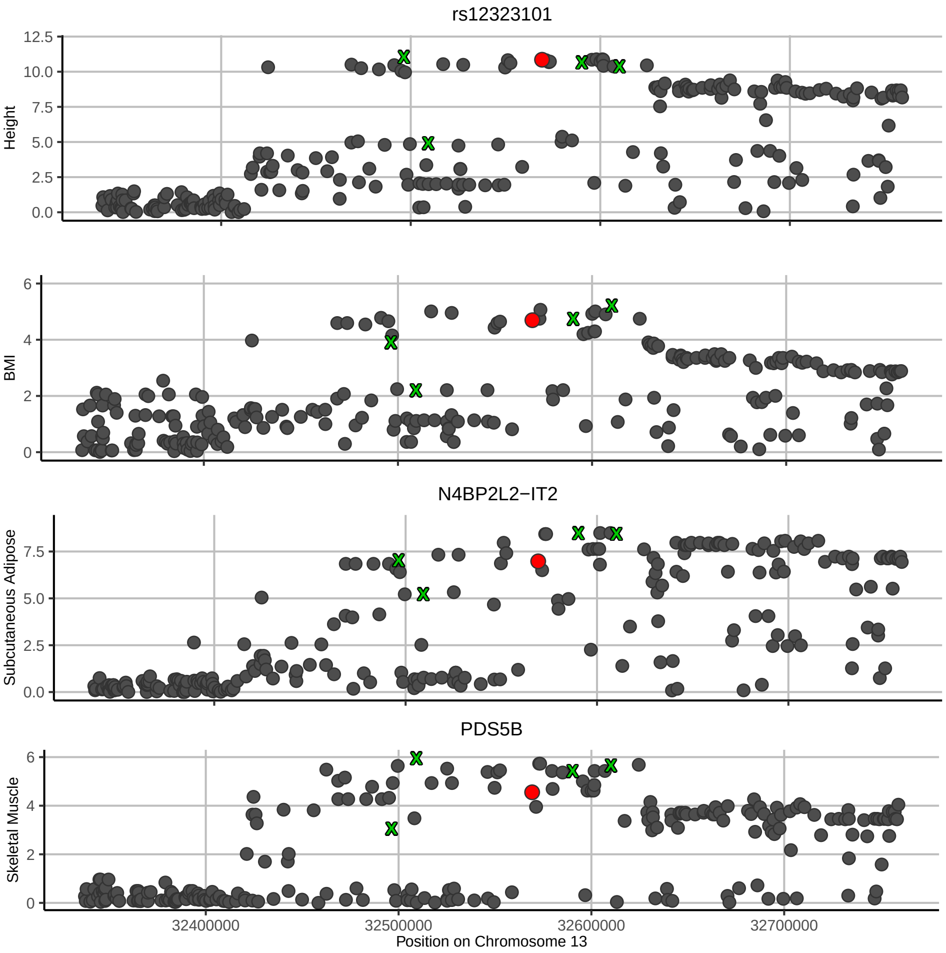
rs12323101
Height
BMI
N4BP2L2−IT2
Subcutaneous Adipose
PDS5B
Skeletal Muscle
32400000
32500000
32600000
32700000
Position on Chromosome 13

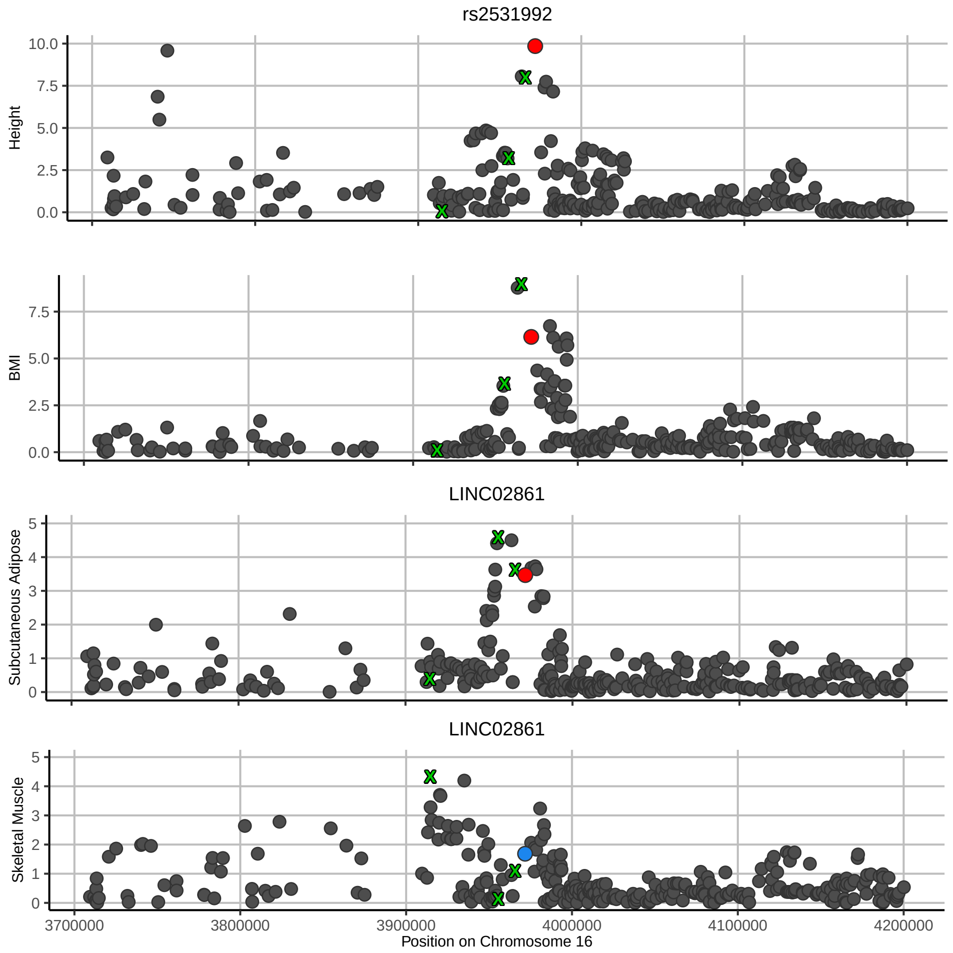

rs2531992
Height
BMI
LINC02861
Subcutaneous Adipose
LINC02861
Skeletal Muscle
3700000
3800000
3900000
4000000
4100000
4200000
Position on Chromosome 16

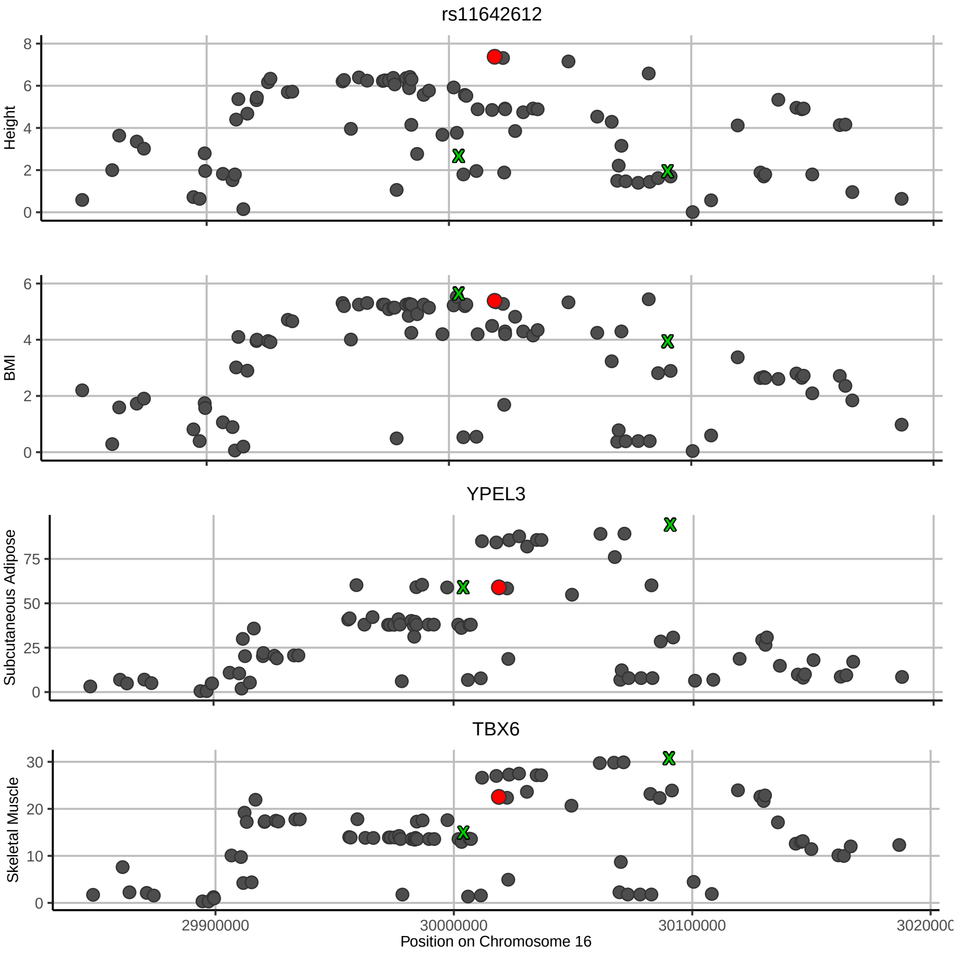

rs11642612
Height
BMI
YPEL3
Subcutaneous Adipose
TBX6
Skeletal Muscle
29900000
30000000
30100000
Position on Chromosome 16

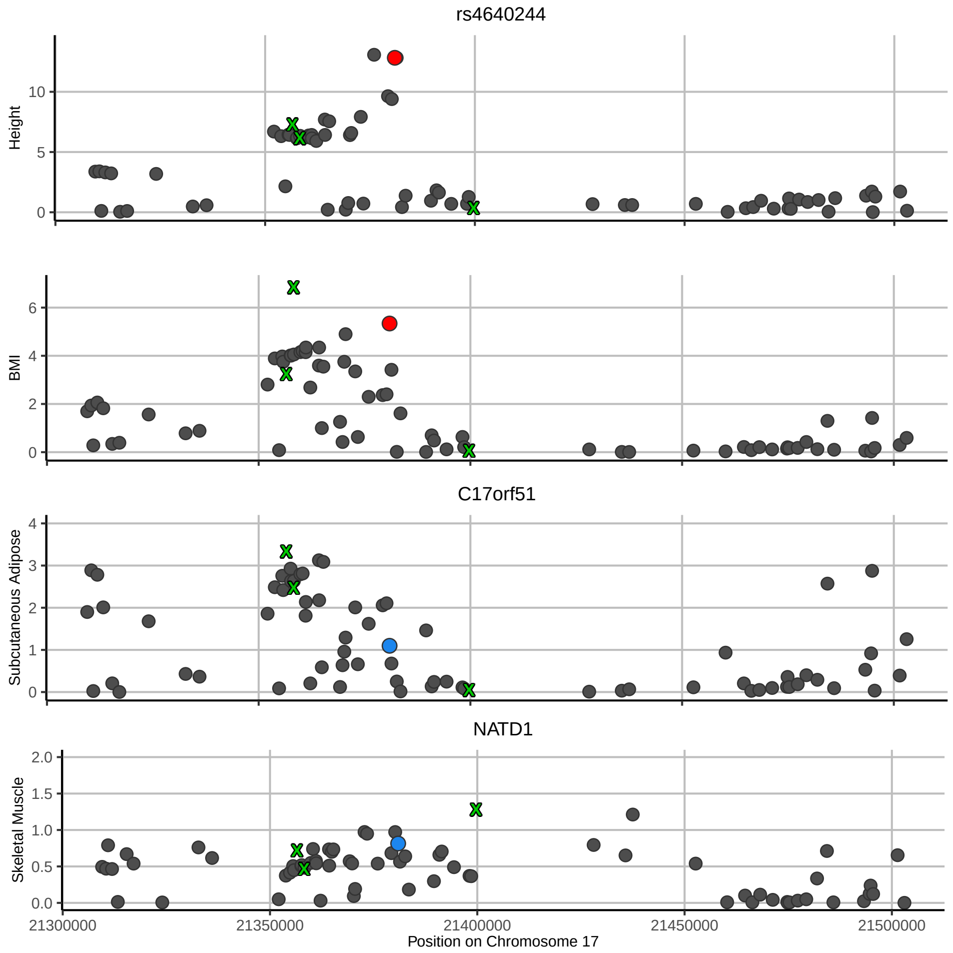

rs4640244
Height
0
5
10
BMI
0
2
4
6
C17orf51
Subcutaneous Adipose
0
1
2
3
4
NATD1
Skeletal Muscle
0.0
0.5
1.0
1.5
2.0
21300000
21350000
21400000
21450000
21500000
Position on Chromosome 17

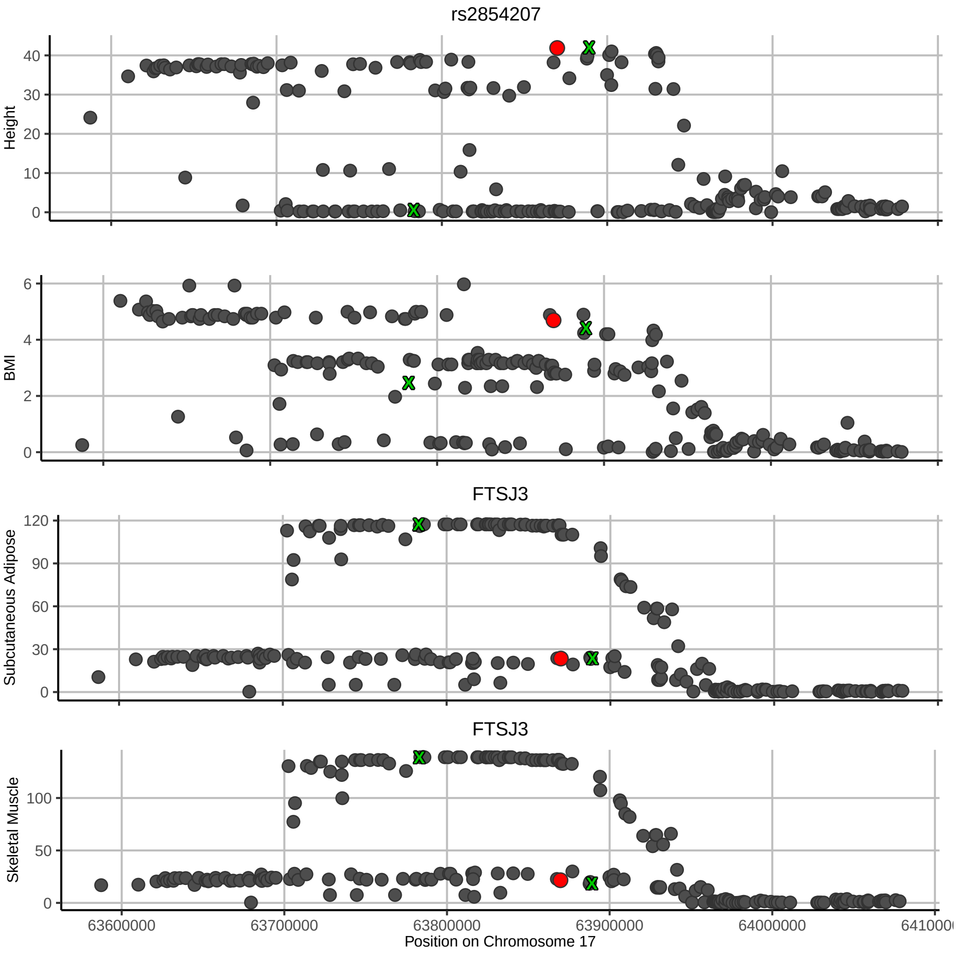
rs2854207
Height
BMI
FTSJ3
Subcutaneous Adipose
FTSJ3
Skeletal Muscle
63600000
63700000
63800000
63900000
64000000
Position on Chromosome 17

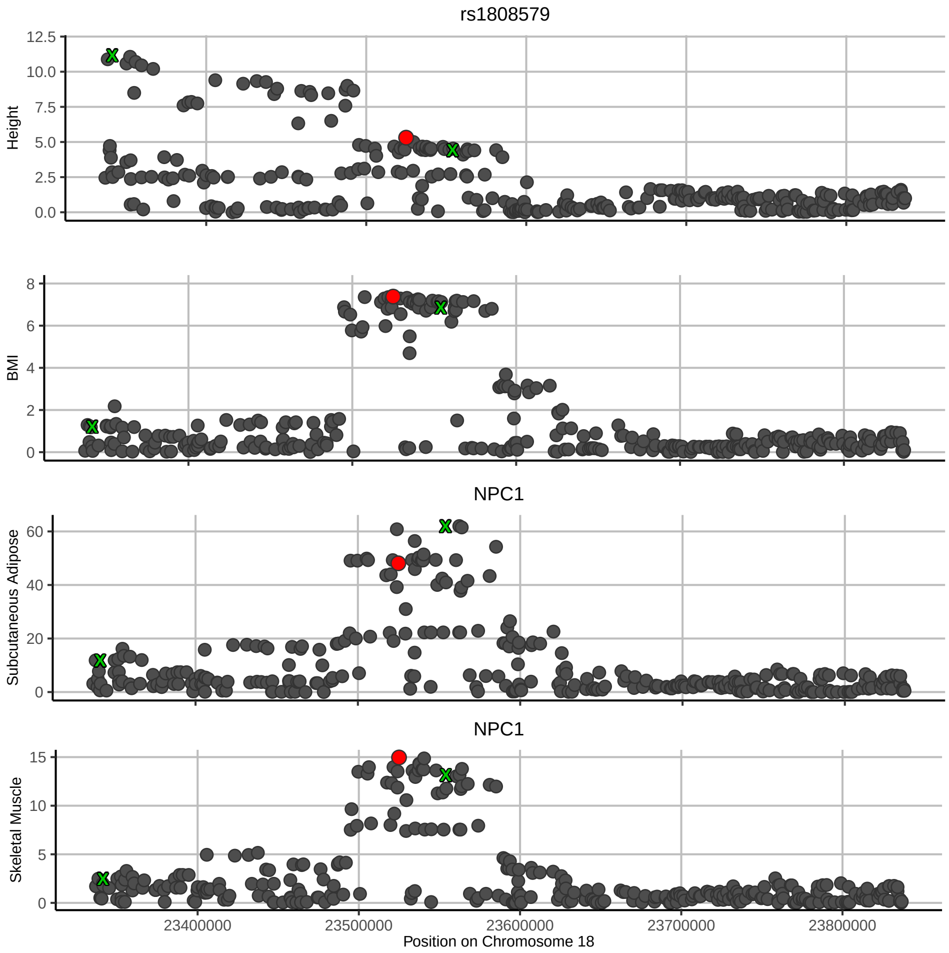
rs1808579
Height
12.5
10.0
7.5
5.0
2.5
0.0
BMI
8
6
4
2
0
NPC1
Subcutaneous Adipose
60
40
20
0
NPC1
Skeletal Muscle
15
10
5
0
23400000
23500000
23600000
23700000
23800000
Position on Chromosome 18

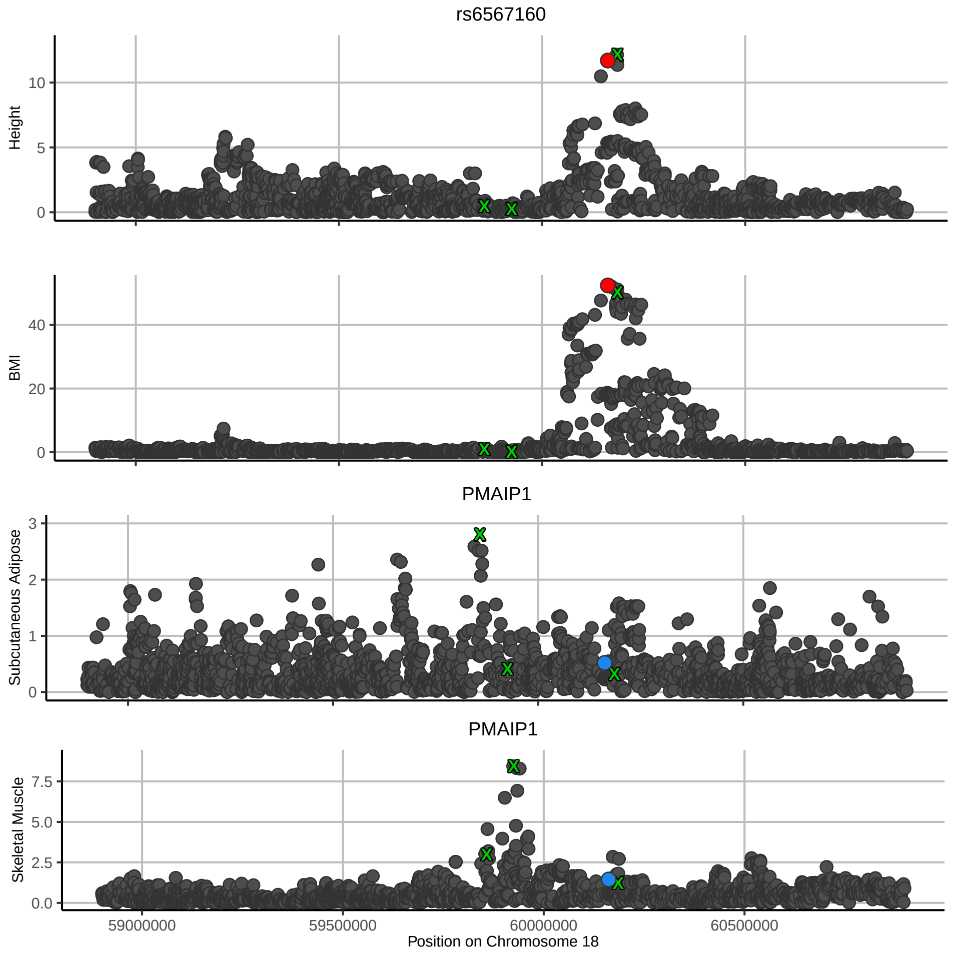
rs6567160
Height
BMI
PMAIP1
Subcutaneous Adipose
PMAIP1
Skeletal Muscle
59000000
59500000
60000000
60500000
Position on Chromosome 18

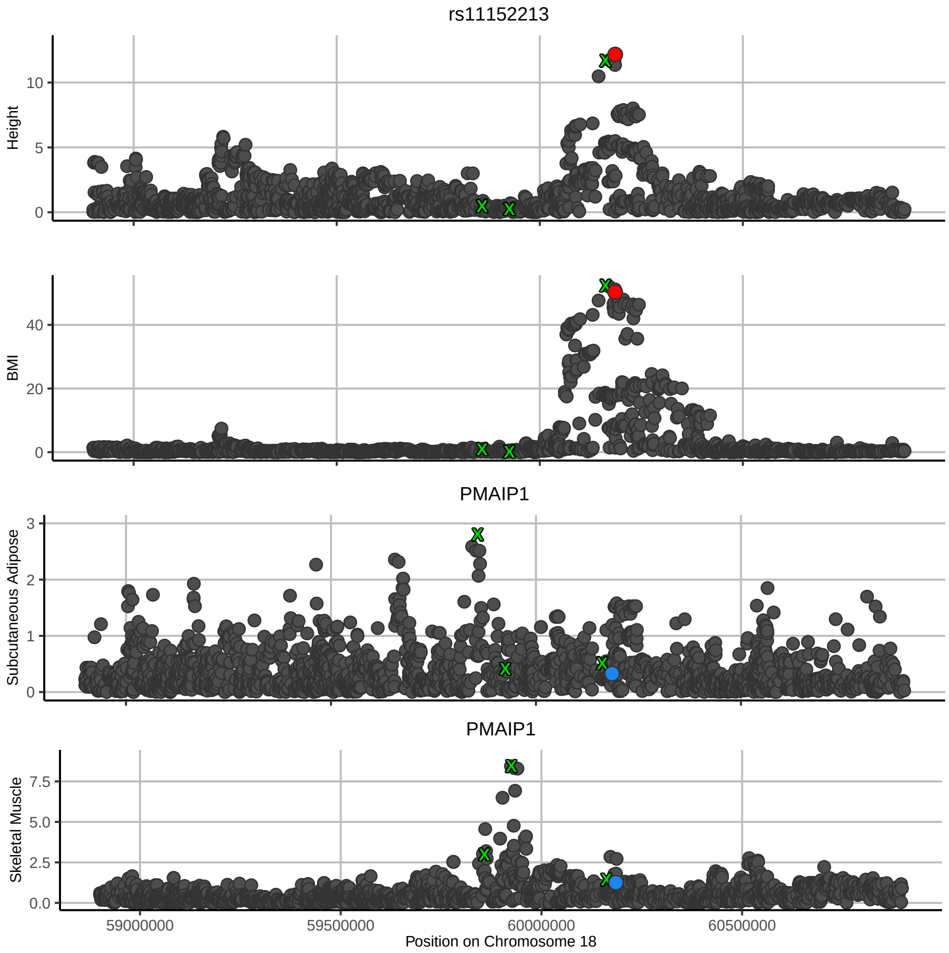

rs11152213
Height
10
5
0
BMI
40
20
0
PMAIP1
Subcutaneous Adipose
3
2
1
0
PMAIP1
Skeletal Muscle
7.5
5.0
2.5
0.0
59000000
59500000
60000000
60500000
Position on Chromosome 18

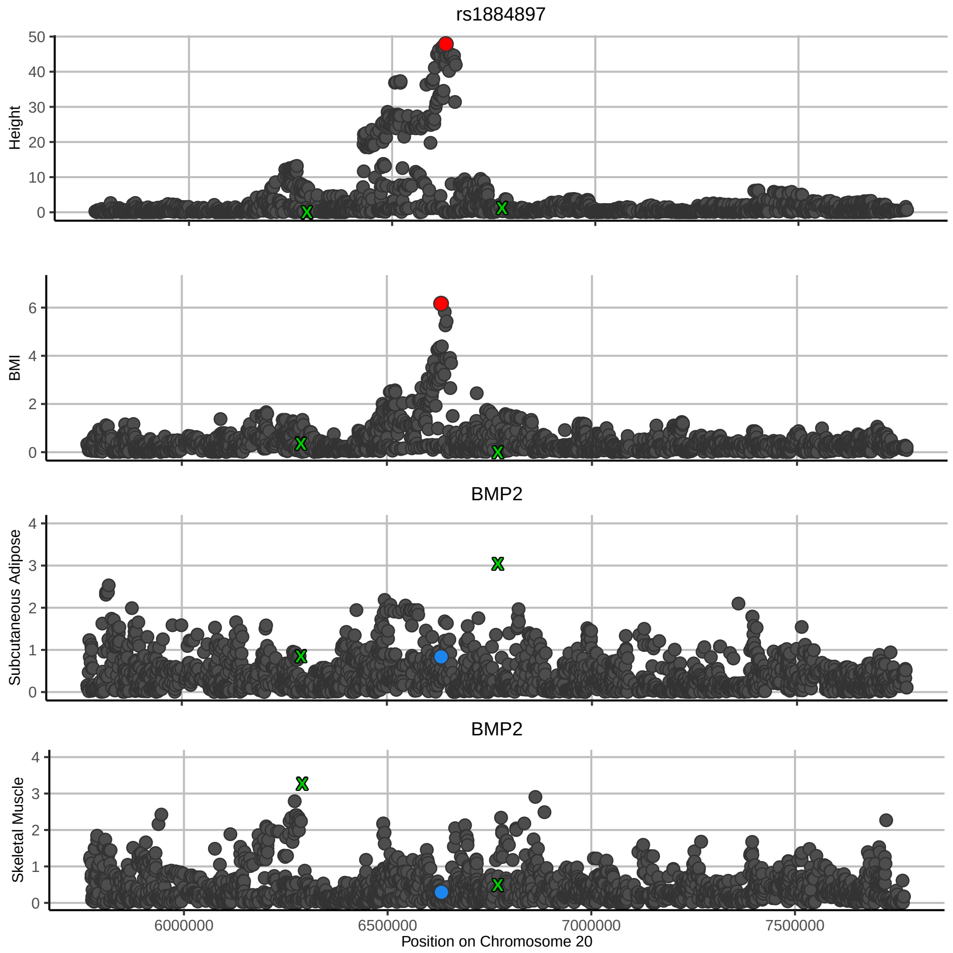

rs1884897
Height
BMI
BMP2
Subcutaneous Adipose
BMP2
Skeletal Muscle
6000000
6500000
7000000
7500000
Position on Chromosome 20
