# Supplementary material for: Primo: integration of multiple GWAS and omics QTL summary statistics for elucidation of molecular mechanisms of trait-associated SNPs and detection of pleiotropy in complex traits
Source: Genome Biol. 2020 Sep 11;21:236. doi: 10.1186/s13059-020-02125-w (PMC7488447; doi:10.1186/s13059-020-02125-w)
Supplement: Supplementary file 2 — Additional file 2 Review history. [file 13059_2020_2125_MOESM2_ESM.docx]

Review History

**First round of review**

**Reviewer 1**

**Were you able to assess all statistics in the manuscript, including the appropriateness of statistical tests used?**

No

**Were you able to directly test the methods?**

No

**Comments to author:**

This paper presents a timely method development to jointly analyze GWAS summary statistics and omic QTLs. The method is rigorous, and its utilities are supported by simulation and two interesting real data analyses.  This is very nice work and potentially has high impact. I do have a few comments and suggestions for the authors to consider.

1. in the introduction section, it was mentioned that "To integrate GWAS and omics QTL summary statistics, several methods have been proposed to identify trait-associated loci that share a common causal variant with 1-2 sets of omics QTLs (often referred to as "colocalization")"

then later

"It is desirable to develop new methods that can integrate multiple (i.e. more than three) sets of GWAS statistics and omics QTL statistics from different conditions/studies while accounting for study heterogeneity, potential sample correlations and linkage disequilibrium (LD)."

It is not clear why the existing methods can only study 1-2 sets of omics QTLs. In addition, do the existing methods "accounting for study heterogeneity, potential sample correlations and linkage disequilibrium (LD)"?

2. This is essentially a mixture distribution problem, where each component of this mixture distribution is one pattern of associations across J studies. What if some patterns do not exist. Does it lead to identifiability problem?

3. It may worth to go deeper to interpret the results from real data analysis. What kind of molecular mechanism has been discovered? Is the number of discoveries in each pattern higher or lower than expected by chance? For the methylation QTL analysis, are those CpGs at island, promoter? What if one apply some simple analysis to compare GWAS with one type of omic data such eQTL?

4. It would be helpful to explain why the t-statistics and p-value based approach have different results. Most readers would think they are equivalent until they read the method section. It seems the reason is that the difference is t-statistics is moderated ones rather than the original ones. At page 12, line 31,

"Here we made a tailored development in the genetic association context by calculating the sample variance for each SNP based on the t-statistic and the minor allele frequency assuming that covariates are independent from genotypes. "

It would be better to give the exact formula. It sounds like one needs both effect size and sample variance estimates to get the moderated t-statistics, but in practice we only have one t-statistic?

5. page 12, line 51

"Under the null hypothesis, the moderated t-statistic follows a t-distribution with a mean of zero and moderated degrees of freedom d_j in the j-th study" Is d_j estimated here or it is derived under null?

6. At page 13, line 26, about approximating t-distribution with normal distribution. How good are the approximation and why it is needed? How was the variance \sigma^{ikj}^2 being derived?

Minor concerns

1. Overall, this paper is well written, though it may benefit from some careful proof readings to simplify the presentation or some re-organizations. For example, the last paragraph of introduction is rather long. It starts with some novel features, and then followed by some summary of the method, and then overview of later sections. It might be better to first describe the components of the methods, then state its novelty in a separate paragraph, and finally use a third paragraph as overview for simulations and real data analyses.

2. page 7, line 18, "The true FDR [42, 44] for Fisher's method calculated from the nominal P-values are not well controlled due to correlations among test statistics, as expected.". Should it be the FDR is not well controlled or the **true FDR** is not well controlled? we cannot control the true FDR?

3. it may be helpful to define the term "lead SNP" upfront.

**Reviewer 2**

**Were you able to assess all statistics in the manuscript, including the appropriateness of statistical tests used?**

Yes: The statistical assumptions of this proposed method lack of theoretical justifications, and not sound in real studies.

**Were you able to directly test the methods?**

No

**Comments to author:**

In this manuscript, the authors proposed a new method for identifying genomic mechanisms of SNPs with respect to multiple (omics) traits, by using the test statistics from single variant tests per trait. This paper target to solve an interesting problem in multi-omics data analysis. However, I have concerns about the model assumptions for this proposed method.

    1. This proposed method is grounded in the assumption that all test statistics from single variant tests are of the same distribution under the NULL hypothesis without association with the trait, or of the same distribution under the alternative hypothesis with association with the trait. However, there is no theoretical results to support this assumption, nor this is what we see in real data. Especially for SNPs under the alternative hypothesis, their test statistic distribution is determined by how each SNP is involved in the causal mechanism of the studied trait, which is expected to vary from locus to locus.

    2. As for using p-values generated by single variant tests, we know that the p-values generated under the NULL hypothesis will follow a Uniform(0, 1) distribution. However, there is no evidence showing that p-values under the Alternative hypothesis are following the same distribution. We often see p-values vary from locus to locus depending on how much association evidence is identified per locus.

    3. When the authors discuss how to account for LD in a conditional analysis, the method is only conditioning on lead omics SNPs in the gene region. The LD among neighborhood SNPs is not discussed. We often seen neighborhood SNPs are highly correlated in GWAS loci. Then how the authors can determine which of the highly correlated SNPs is the true causal SNP, or the true SNP involved in the multi-omics mechanism?

    4. Note that the authors assume t-distributions for the test statistic from single variant test, which makes sense. But in the conditional analysis, the authors assume all test statistic are following a Gaussian distribution. How should we understand this conflict?

    5. In the simulation, I think simulating test statistics directly from the assumed t-distributions is not realistic. Instead, I think SNP effect-sizes should be first simulated from a normal distribution for those causal SNPs, then simulate phenotypes and perform single variant tests to obtain test statistics.

    6. As shown in the simulation results in the supplementary text, the method is sensitive to the pre-specification of the π_k values, especially when over-specifying the π_k values.

    7. In the application results, I am wondering what proportion of the identified SNPs with a particular association pattern actually have significant single variant test p-values for at least one trait? How we might be able to validate these findings?

    8. I did not see any figure legends included in the manuscript, which make it very hard to understand all figures.

**Authors Response**

**Point-by-point responses to the reviewers’ comments:**

Response to reviewers for the manuscript 'Primo: integration of multiple GWAS and omics QTL summary statistics for elucidation of molecular mechanisms of trait-associated SNPs and detection of pleiotropy in complex traits'

*We first would like to thank the AE and two reviewers for taking the time and making valuable suggestions to our manuscript. We believe that we have addressed all the reviewers’ comments, clarifying details regarding models and assumptions, and provided additional simulations and empirical evidence supporting our models and assumptions. Below we provided point-to-point responses to both reviewers’ comments.*

Reviewer #1: This paper presents a timely method development to jointly analyze GWAS summary statistics and omic QTLs. The method is rigorous, and its utilities are supported by simulation and two interesting real data analyses. This is very nice work and potentially has high impact. I do have a few comments and suggestions for the authors to consider.

1.	In the introduction section, it was mentioned that "To integrate GWAS and omics QTL summary statistics, several methods have been proposed to identify trait-associated loci that share a common causal variant with 1-2 sets of omics QTLs (often referred to as "colocalization")" then later "It is desirable to develop new methods that can integrate multiple (i.e. more than three) sets of GWAS statistics and omics QTL statistics from different conditions/studies while accounting for study heterogeneity, potential sample correlations and linkage disequilibrium (LD)."

It is not clear why the existing methods can only study 1-2 sets of omics QTLs. In addition, do the existing methods "accounting for study heterogeneity, potential sample correlations and linkage disequilibrium (LD)"?

*Our Response:*

*In the following paragraphs, we individually review existing widely-used colocalization methods: coloc, moloc, enloc and eCAVIAR. To summarize, the existing methods can only study 1-2 sets of omics QTLs with GWAS at a time. The three methods, coloc, enloc and eCAVIAR were explicitly developed to study exactly 2 traits at a time (one complex trait from GWAS and one omics trait from an omics QTL study), and moloc is designed for analysis of exactly 3 traits (one complex trait from GWAS and two multi-omics traits from QTL studies). In analyzing more than 3 traits, one may apply existing methods to pairs of multiple traits, however, the multiple testing adjustment and the composite hypothesis testing become complicated. That motivates the current work.*

*None of the existing methods account for potential sample correlations in their analyses. This might be because, if considering only one GWAS and one omics QTL study, it is reasonable to assume that GWAS and omics QTL studies are from different samples for existing methods. All existing methods allow for the presence of LD, but only eCAVIAR incorporates LD correlation estimates directly. Study heterogeneity is not discussed by the methods, but by performing colocalization using only summary statistics of different traits, all methods should have accounted for study heterogeneity to some degree. Thus, the primary innovation of Primo is the extension to integrate summary statistics from more than three studies or phenotypes (e.g. 1 GWAS and > 2 QTL studies; or multiple GWAS and multiple QTL studies). And in this setting, since multi-omics traits may be measured from independent or overlapping samples, methods to account for sample correlations are necessary while existing methods did not address this point. As such, carefully accounting for potential sample correlations among different traits from different studies is another key innovation of the work.*

*More specifically,*

*coloc (PMID:24830394) is a Bayesian statistical test for colocalization that analyzes exactly two traits in a single genomic region to quantify the probability that the two traits share one causal variant in the region. The authors note that a major focus is interpreting patterns of (inferred) LD in the locus. The method assumes that traits are measured in unrelated individuals and that effect sizes for the two traits are independent. coloc also assumes that there is no more than one causal variant for each trait in the region, an assumption that is violated in regions harboring allelic heterogeneity as discussed in PMID:27866706. It requires specification of prior probabilities for being associated with only the first trait, only the second trait and both traits. As discussed and demonstrated in PMID:28278150, analysis results may be sensitive to this prior specification.*

*moloc (PMID:29579179) extends methodology of coloc to exactly three traits as is implemented in its R package. Like coloc, moloc assumes that traits are measured in unrelated individuals, that effect sizes for the traits are independent, and that there is no more than one causal variant for each trait in the region. The method requires prior specification for the probabilities of each causal association pattern (referred to as “configurations”). And for J=3, that is a total of 2𝐽 = 8 prior parameters.*

*enloc (PMID:28278150) uses a Bayesian hierarchical model that treats the latent association status of omics QTLs as variant-level annotations for candidate SNPs of complex traits in a regression framework. It is implemented to study exactly one complex trait GWAS with exactly one QTL study. In recognition of the importance of LD, the method studies one LD block at a time, but it does not directly incorporate LD correlation estimates in its analysis. enloc does not adjust for potential sample correlation between the two traits being analyzed.*

*eCAVIAR (PMID: 27866706) is a colocalization method that performs Bayesian fine- mapping within each LD block to obtain estimates of the posterior inclusion probability for each variant of being causal in a GWAS study and an eQTL study. By assuming that the prior probability of the same variant being causal in both a GWAS and eQTL study is independent, the colocalization posterior probability for each variant can be obtained as the product of the posterior probabilities of causality in each trait. In its analysis, eCAVIAR incorporates LD correlation estimates, which can be obtained from reference panels if individual level data is not available. The method does not adjust for potential sample correlation between the two traits being analyzed.*

*Changes we made: On page 17-18 of the main text in the subsection “The connection of Primo to “colocalization” and meta-analysis methods”, we summarized the main points above.*

2.	This is essentially a mixture distribution problem, where each component of this mixture distribution is one pattern of associations across J studies. What if some patterns do not exist. Does it lead to identifiability problem?

*Our Response:*

*The reviewer is correct that the problems motivating Primo are essentially mixture distribution problems. When J is low (2-3), there are only a limited number of possible patterns and most of the patterns exist in the genome. When J increases, as in our work, we explored J=3- 5, some of the patterns may not exist or may be indistinguishable. Here we would like to clarify and emphasize one point. Most of the inference presented in the work are based on collapsed patterns when J is relatively large and some specific patterns do not exist.*

*The motivating analysis of the method is to provide a comprehensive multi-omics mechanism interpretation of known GWAS SNPs and study how they affect complex traits. As illustrated in Figure 1, when J is relatively large and there are too many possible patterns, many of the patterns can be flexibly collapsed. That way, Primo can provide a fairly flexible quantification of some specific patterns of interest. As an example, in Figure 1C when studying GWAS with e-, me- and pQTL statistics, one may be interested in the collapsed pattern of a GWAS SNP being also a multi-omics QTLs and one can quantify the probabilities by collapsing patterns 10-16. As another example, if one is interested in the GWAS SNP being also an eQTL, one may collapse patterns involving associations to a complex trait and expression trait regardless of association status to other omics traits (Patterns 10, 13, 14 and 16) in Figure 1C. Since all of the patterns are mutually exclusive, when collapsing those patterns, one only needs to add together the posterior probabilities of those patterns of interest to quantify the joint probability. Therefore, unlike some mixture distribution problems, where the assignment of the SNPs to each specific pattern is of interest, Primo benefits from the knowledge of all possible mixture components and we are most interested in the collapsed patterns that do exist in the genome and can be interpreted (etc. GWAS SNP being also an eQTL). Additionally, the collapsed patterns alleviate the known challenge in mixture distribution problems when the individual mixing components are not distinguishable. Meanwhile, the assignment of known GWAS SNPs to collapsed patterns is also more robust in comparison with assigning to one of the specific patterns.*

*In terms of identifiability, as illustrated in Figure 1A, Primo separated the pattern-specific joint density (𝐷𝑘) estimation and the estimation of 𝜋𝑘’s in two separate steps. This is different than simultaneously estimating both in most mixture distribution problems. Therefore, identifiability is not an issue. In the subsection “Primo as a general framework for assessing joint associations across data types” in the Results section, we discussed this point: “In estimating a mixture distribution of K (K=2𝐽) components, the performance of estimation and subsequent inference depend on how well different mixing components separate from each other. When K is moderate to large, it is challenging to simultaneously estimate the distributions of mixing components 𝐷𝑘's and the mixing proportions 𝜋𝑘's. Different from previous work, Primo first estimates the pattern- specific multivariate density function 𝐷𝑘 for each of the association pattern by borrowing information across SNPs and traits. See Methods section for detailed estimation procedures when J sets of association statistics were calculated from independent or correlated samples. Then Primo estimates 𝜋𝑘's via the Expectation-Maximization algorithm. When 𝐷𝑘's are reasonably- estimated, the one-step estimates of 𝜋𝑘's can well capture the overall proportions of different association patterns and there is no need to re-iterate and re-estimate 𝐷𝑘's and 𝜋𝑘's. …”*

*Furthermore, we have confirmed the performance of Primo in the absence of some patterns through simulations. In many simulations, we only simulated SNPs of selected patterns, confounding patterns, but not all possible patterns. And when we are also focusing on the selected, confounding patterns for inference, the performance is still quite good.*

*Changes we made: we added some discussions of collapsed patterns in the results section page 5, second full paragraph.*

3.	It may worth to go deeper to interpret the results from real data analysis. What kind of molecular mechanism has been discovered? Is the number of discoveries in each pattern higher or lower than expected by chance? For the methylation QTL analysis, are those CpGs at island, promoter? What if one apply some simple analysis to compare GWAS with one type of omic data such eQTL?

*Our Response:*

*We thank the reviewer for this suggestion since we believe the “deeper” interpretation has added significant value to the real data analysis. As discussed in the paper, we identified 17 breast cancer susceptibility loci uniquely associated with cis-methylation levels but not expression levels in either tumor or normal tissues. As such, those GWAS loci might affect breast cancer risk uniquely via modulation of cis-methylation levels rather than cis-transcriptional expression levels. In follow-up analysis, we compared the CpG targets of the cis-methylation-only associated loci (“meQTL only”) to CpG targets of loci also associated with expression levels (“e-meQTLs or e- me-pQTLs, i.e., multi-omics QTLs”) and checked for enrichment among several bio features. These included locational relation to CpG islands, exons/introns, and promoter/enhancer regions. Figure A below (Figure 4A in the manuscript) compares the distribution of locational relation to CpG islands for “meQTL only” CpG targets and “multi-omics QTL” CpG targets identified by Primo to all CpG sites on the Illumina 450k array. Compared to randomly selected CpG sites, CpG targets of the identified multi-omics QTLs were more likely to be in CpG Island (CpGI) shores (p < 0.05) and less likely to be in open sea regions (p < 0.01). The enrichment in shores is consistent with literature reporting the importance of DNA methylation alterations in CpGI shores in cancer cells   and the relationship between CpGI shore methylation and geneexpression (PMID: 19151715; 21821115; 28155687).*

*We also checked for enrichment of the two types of CpG targets (multi-omics QTL verus meQTL only) in introns, exons, enhancers and promoters. The results are presented in Figure B below (Figure 4B in the manuscript). CpG targets of multi-omics QTLs were enriched in exons (p< 0.01) and promoter regions (p < 0.001) as might be expected given the roles of those features in gene transcription. CpG targets of meQTL only loci were very depleted in promoter regions (p < 0.0001), which also may be expected since hypo/hypermethylation of CpG sites in these regions would be expected to affect global transcription levels. CpG targets of meQTL only breast cancer susceptibility loci were enriched in introns (p < 0.001) and enhancer regions (p < 0.01). A recent review article of the role of Pre-mRNA splicing noted that DNA methylation in cis-acting splicing regulatory elements (such as intronic splicing silencers or enhancers) is associated with various cancers, including breast cancer (PMID: 30464224), but future research is needed to develop a comprehensive picture of the cellular processes underlying these associations.*

Figure A. Distributions of relation to CpG island of CpG targets of Primo identified meQTLs.

Figure B. Fold enrichment or depletion in genomic features of CpG targets of Primo identified meQTLs.

*Because estimates of the proportion of SNPs that are QTLs vary in the literature, we use the analyzed data to estimate whether the number of discoveries in each pattern is higher or lower than by chance. As noted in response #7 to reviewer 2, each GWAS SNP-gene-methylation- protein quartet found to be significant for “at least one QTL” has at least one marginal QTL P- value < 0.00069. In the analyzed data, 14.0%, 6.2%, 17.6% and 0.71% of SNPs have a marginal p-value < 0.00069 for normal gene expression, tumor gene expression, tumor DNA methylation, and tumor protein abundance, respectfully. Using these estimates, the expected proportions of SNPs expected to be associated with at least 1, 2, 3, and 4 traits, respectively, by chance are: 34.0%,* ***4.3%, 0.2% and 0.001****%. The observed proportions of SNPs associated with at least 1, 2,*

*3, and 4 traits, respectfully are: 32.5%,* ***16.6%, 5.7%, 0.6%.*** *Therefore,* ***the method identified many more SNPs associated with at least 2 traits, at least 3 traits and all 4 traits than expected by chance alone*** *(the observed proportion associated with at least 1 should be similar to the expected proportion given how the marginal expectations were estimated).*

*Simple analyses that compare GWAS with one or more omicsQTL studies would not achieve similar results. As discussed above, Primo accounts for several key aspects in analyzing and integrating GWAS with multi-omics QTL summary statistics, including accounting for sample overlapping, LD, sample heterogeneity, as well as the complications in analyzing multiple traits from different studies with only summary statistics. For example, if a GWAS SNP is also having an eQTL p-value of 0.001, by simple analyses examining the eQTL p-value of a GWAS SNP, one may identify the GWAS SNP being also an eQTL. However, that may not be true. As shown in Figure 2 second panel, the GWAS SNP may be having a low eQTL p-value simply because it is in LD with another true eQTL. Primo analysis would adjust for other lead SNPs in the region and account for LD, and will report whether the GWAS SNP remains an eQTL after adjusting for other lead SNPs. Figure 3 presented another real example that in the region of the gene KLHDC7A, if one only analyzed GWAS and normal eQTL statistics from GTEx, the GWAS SNP has a relatively low eQTL p-value while if jointly analyzing omics QTLs also from tumor samples from TCGA, the GWAS SNP rs2992756 is identified as a multi-omics QTL in the tumor samples. That shows the advantages of our method over existing methods that can only analyze 1 GWAS + 1 omics QTL at a time.*

*Changes we made: We added additional interpretations and enrichment analysis of the breast cancer susceptibility loci uniquely associated with cis-methylation levels and those associated with both cis-methylation levels and cis-gene expression in either tumor or normal tissues. See page 10, last full paragraph, in the main text. We also added a new Figure 4.*

4.    It would be helpful to explain why the t-statistics and p-value based approach have different results. Most readers would think they are equivalent until they read the method section. It seems the reason is that the difference is t-statistics is moderated ones rather than the original ones. At page 12, line 31, "Here we made a tailored development in the genetic association context by calculating the sample variance for each SNP based on the t-statistic and the minor allele frequency assuming that covariates are independent from genotypes. " It would be better to give the exact formula. It sounds like one needs both effect size and sample variance estimates to get the moderated t-statistics, but in practice we only have one t-statistic.

*Our Response:*

*The reviewer is correct that one reason for the slight differences in results between the t- statistic and p-value based approaches is the use of the moderated t-statistics, since the moderated t-statistics will adaptively estimate the empirical null distribution as a t-distribution with a moderated degrees of freedom. Other slight differences may arise from the fact that the estimation procedures use different distributions (e.g. scaled t-distribution versus mixture of chi-squared) and may not estimate parameter values that yield equivalent results. Nonetheless, as shown in simulations, there is substantial overlap and similarity in results produced by the two different methods.*

*To use the t-statistics version of Primo, users provide the effect size estimates (e.g. regression coefficients) and their standard errors. In practice (i.e. in public datasets), quite often effect size and standard error estimates are available if t-statistics are provided. If for some reason the effect sizes and standard errors are not available, users may instead use the p-value based approach of Primo (if not directly provided, p-values could be calculated from the provided t- statistics). Additionally, if F-tests or other second-order tests are used in association analysis, or if one-sided t-tests/p-values are preferred when a same direction of association effects are expected for biological reasons, then the p-value version of Primo would be helpful.*

5.	page 12, line 51 "Under the null hypothesis, the moderated t-statistic follows a t- distribution with a mean of zero and moderated degrees of freedom d_j in the j-th study" Is d_j estimated here or it is derived under null?

*Our Response:*

*The moderated degrees of freedom, 𝑑𝑗, is estimated using the limma algorithm described by Smyth (PMID:16646809). We have avoided repeating derivations from Smyth’s landmark work in our manuscript so as not to confuse readers about our contributions and developments versus existing ones. Here we provide relevant information for context.*

*The moderated degrees of freedom, 𝑑𝑗, is calculated as the sum of two terms: 𝑑𝑗   = 𝑑0𝑗     +*

*𝑑1𝑗. Here, 𝑑1𝑗  is the (original) degrees of freedom of the summary statistics in study 𝑗.  𝑑0𝑗  is  the*

*degrees of freedom hyperparameter for the prior on the unknown variances, 𝜎2, of effect sizes. In practice, 𝑑0𝑗 is estimated following an empirical Bayes approach, using moments of a scaled F- distribution as described by Smyth (PMID:16646809, section 6.2). For estimation, we use functions implemented in the limma package in R, which is available on Bioconductor. The parameter 𝑑0𝑗  is analogous to a tuning parameter in the ridge penalty.*

*In the manuscript, we simplified the discussion by assuming that the degrees of freedom were the same for all variants in study j (same # covariates). Note that the Primo R package does allow SNPs’ degrees of freedom vary, though the moderated degree of freedom 𝑑0𝑗 is estimated by and shared among all SNPs, and so if 𝑑1𝑗 vary by SNP, then the moderated degrees of freedom are SNP-specific: 𝑑𝑖𝑗   = 𝑑0𝑗  +  𝑑1𝑖𝑗.*

*Those details are provided on page 14 in the main text.*

6.	At page 13, line 26, about approximating t-distribution with normal distribution. How good are the approximation and why it is needed? How was the variance \sigma^{ikj}^2 being derived?

*Our Response:*

*In estimating the pattern specific density functions, 𝐷𝑘, for 𝐾 = 2𝐽 patterns, we first obtained the marginal (study-specific) empirical null and alternative distributions as t- distributions, approximated the t-distributions with normal, then incorporated the same estimated sample correlation matrix estimated under the null pattern to estimate the multivariate density function 𝐷𝑘’s as a multivariate normal distribution. The reviewer is correct that one may also directly use a multivariate t-distribution instead of normal approximation. The difference would be minor since most of the summary statistics are calculated from a study with a relatively large sample. So normal well approximates t-distributions. We chose to use normal for three reasons: first, using the normal approximation for the t-distribution will gain computational efficiency. The multivariate  normal  density  calculations  implemented  in  R  are  quite  fast.  Second,  in     the conditional Primo analysis adjusting for confounding SNPs, the conditional distribution of multivariate normal is statistically and computationally much easier than conditional distribution of multivariate t-distributions. Third, some GWAS summary statistics for binary traits may be based on Z-tests rather than t-tests and the test-statistics (for log odds ratios) are truly normally distributed.*

*Changes we made: We edited the main text on page 14 to clarify those details.*

Minor concerns

1.	Overall, this paper is well written, though it may benefit from some careful proof readings to simplify the presentation or some re-organizations. For example, the last paragraph of introduction is rather long. It starts with some novel features, and then followed by some summary of the method, and then overview of later sections. It might be better to first describe the components of the methods, then state its novelty in a separate paragraph, and finally use a third paragraph as overview for simulations and real data analyses.

*Our Response: Agreed. We rearranged the last paragraph of introduction as suggested. See page 2-3.*

2.	page 7, line 18, "The true FDR [42, 44] for Fisher's method calculated from the nominal P-values are not well controlled due to correlations among test statistics, as expected.". Should it be the FDR is not well controlled or the **true FDR** is not well controlled? we cannot control the true FDR?

*Our Response: We thank the reviewer for catching the imprecise wording. The goal in using the term “true FDR” was to differentiate the “true” FDR (# false positive findings/ # significant findings) across simulations from the FDR estimated by Storey’s q-value method based on p- values from Fisher’s method. We have corrected the wording in the resubmission. See page 7, end of the second full paragraph.*

3.	it may be helpful to define the term "lead SNP" upfront.

*Our Response: We added a definition of lead SNP on page 2: “a ‘lead SNP’ is the SNP with the smallest association P-value with a particular trait in the region”.*

Reviewer #2:

In this manuscript, the authors proposed a new method for identifying genomic mechanisms of SNPs with respect to multiple (omics) traits, by using the test statistics from single variant tests per trait. This paper target to solve an interesting problem in multi-omics data analysis. However, I have concerns about the model assumptions for this proposed method.

1.	This proposed method is grounded in the assumption that all test statistics from single variant tests are of the same distribution under the NULL hypothesis without association with the trait, or of the same distribution under the alternative hypothesis with association with the trait. However, there is no theoretical results to support this assumption, nor this is what we see in real data. Especially for SNPs under the alternative hypothesis, their test statistic distribution is determined by how each SNP is involved in the causal mechanism of the studied trait, which is expected to vary from locus to locus.

*Our Response:*

*The reviewer is astute to point out that the alternative distributions may vary from locus to locus. The test statistics under the NULL are assumed to follow the same distribution across variants and loci. In the moderated-t version of Primo, we estimated the empirical null distributions for each study as moderated t-distributions (i.e., t-distributions with estimated degrees of freedom, and the degrees of freedom was estimated from the empirical data using the limma package). In the p-value version of Primo, we assume that the null distribution of –2log(P-value) follows a chi- square distribution with degree of freedom of 2.*

*However, it should be noted that the method actually does not assume that test statistics follow the same exact distribution under the alternative. Under the alternative, the test-statistics from the moderated-t version of Primo are assumed to follow scaled t-distributions, but the scaling factor for the alternative distribution of SNP 𝑖, 𝑣𝑖𝑗, is SNP-specific. To simplify discussion and not generate confusion over our contributions versus those of Smyth (PMID:16646809), we avoided discussion of how the limma algorithm estimates the scaling factor in the manuscript. To paraphrase Smyth, the moderated t-statistic 𝑡̃𝑖𝑗 for SNP 𝑖 follows a scaled t-distribution under the alternative hypothesis:*

*where 𝑣0𝑗 is the variance hyperparameter for the prior placed on nonzero effect size coefficients and 𝑤𝑖𝑗 is a SNP-specific weight for SNP 𝑖. In the Primo analysis presented in the manuscript, we set 𝑤𝑖𝑗 = 1/(2𝑁𝑗 ∗ MAF𝑖 ∗ (1 − MAF𝑖)) for different SNPs. This is consistent with the literature assuming variant effect sizes are inversely proportion to MAF. When covariates information is available, we may set 𝑤𝑖𝑗 = (𝑋𝑖𝑗′𝑋𝑖𝑗)−1 where 𝑋𝑖𝑗 is the covariate for SNP 𝑖 in study 𝑗. Potentially, other information such as annotation can also be incorporate to estimate the SNP-specific empirical alternative distributions via specification of 𝑤𝑖𝑗 if those information are available. Via the limma algorithm, we estimate 𝑣0𝑗 from the data, using the standard errors of the effect size coefficients, degrees of freedom, and minor allele frequency information. Then the scaling factor is 𝑣𝑖𝑗  = (1 + 𝑣0𝑗/𝑤𝑖𝑗)1/2.*

*Changes we made: To clarify this point, we added additional explanation in the Methods section. See page 13-14.*

2.	As for using p-values generated by single variant tests, we know that the p-values generated under the NULL hypothesis will follow a Uniform(0, 1) distribution. However, there is no evidence showing that p-values under the Alternative hypothesis are following the same distribution. We often see p-values vary from locus to locus depending on how much association evidence is identified per locus.

*Our Response:*

*In the P-value-based Primo, the alternative distribution for each locus are indeed assumed to be different. Here we provide some more details.*

*Under the alternative hypothesis, for each locus, the negative 2 of log-transformed p-values will follow a non-central chi-squared distribution. When analyzing all loci in the genome, negative 2 of log-transformed p-values are assumed to follow a mixture of different (non-central) chi- squared distributions. Statistical literature has shown that a mixture of chi-squared distributions with different degrees of freedom can be well-approximated by a single scaled chi-squared*

*distribution with a different degree of freedom,  ∑𝑚 𝑤𝑖𝜒2   ≈ 𝐴𝜒2          (Solomon H, Stephens MA.*

*𝑖	𝑑𝑖	𝐵*

*Distribution of a Sum of Weighted Chi-Square Variables. J Amer Statist Assoc.1977;72:881– 885). Therefore, we agree that the alternative distribution may vary from locus to locus, and indeed that was reflected in our model and algorithm. In our algorithm, the overall empirical alternative distribution of a specific study (a mixture of chi-squared distributions) can be well approximated by a single chi-squared distribution. In other words, the single chi-squared distribution under the alternative is a mathematical approximation of the mixture of alternative distributions for different loci, rather than an assumption.*

*In the presented manuscript, we also have used simulations to demonstrate the robustness of our method to the presence of multiple alternative distributions (e.g. as may vary across loci). In Scenario 2 (page 7, paragraph 1), the effect sizes were simulated from N(0,𝜎2) distributions, where 𝜎2 = 0.25, 0.5 and 1 each in one third of loci. Thus, the alternative distributions of p-values varied by loci. As shown in the simulation results, the p-value based version of Primo performed well in the simulations.*

*Changes we made: To further clarify the confusion, we added additional details in the manuscript on page 16, second paragraph.*

3.	When the authors discuss how to account for LD in a conditional analysis, the method is only conditioning on lead omics SNPs in the gene region. The LD among neighborhood SNPs is not discussed. We often seen neighborhood SNPs are highly correlated in GWAS loci. Then how the authors can determine which of the highly correlated SNPs is the true causal SNP, or the true SNP involved in the multi-omics mechanism?

*Our response:*

*Similar to colocalization methods in the literature, the goal of Primo is not to identify the true causal SNP, but rather given a known trait-associated SNP, the goal of Primo here is to explain how the trait-associated SNP may affect the complex trait of interest via potential multi-omics mechanisms in which cellular contexts. In both applications, although we used the data from the entire genome to estimate the key parameters needed in Primo analysis, our analyses actually focus on only the known GWAS SNPs. The purpose of our analysis is to answer whether the GWAS SNPs affect the complex trait of interest through modulation of expression/methylation/protein abundance levels of which cis-gene in which cellular context. This question is not trivial as there are often many cis-genes and there are potentially many cis-multi-omics traits and many cell types. Since the mechanism can be dynamic, i.e., GWAS SNP might affect a cis-gene expression in only certain tissue types or cell types, it is warranted to have a method/algorithm that can jointly analyze multiple sets of omicsQTL summary statistics from independent or overlapping samples.*

*So in the proposed analysis, a major confounding factor are the neighborhood SNPs that are omics QTLs. As shown in Figure 2 in the main text, we focus on only the GWAS SNP (red/blue dot in the Figure) and consider other omics QTLs in the neighborhood, and Primo will assess whether the GWAS SNP is an eQTL/meQTL/pQTL conditional on other lead SNPs.*

*For identifying true causal SNPs, when neighborhood SNPs are in high or even perfect LD with the GWAS SNP of interest, using statistical methods alone may not be able to identify true causal SNPs.  Again this is not the goal of Primo.*

*Primo is a general framework for multi-study association analysis. One may potentially apply Primo in other analyses to identify multi-omics QTLs with only sets of omics QTLs summary statistics as input, but no GWAS statistics. But the goal of the analysis would be different than what is presented in the current work, and interpretations would also be different.*

*Changes we made: We apologize for the confusion and this is in fact a key point. We thank the reviewer to point it out. We now have emphasized the point that we are not aiming to identify causal GWAS SNPs in the current work, but rather, the goal is to focus on only known trait- associated (GWAS) SNPs and to use multi-omics summary statistics from different cellular contexts to comprehensively interpret how GWAS SNPs may affect the complex trait(s). See page 3 the last paragraph of introduction and also the first paragraph in the discussion section on page 12.*

4.	Note that the authors assume t-distributions for the test statistic from single variant test, which makes sense. But in the conditional analysis, the authors assume all test statistic are following a Gaussian distribution. How should we understand this conflict?

*Our Response:*

*We use the Gaussian approximation for t-distributions for three reasons: 1) when samples of multiple studies are correlated, we could estimate the sample correlations under the null and use it in the multivariate density functions to capture sample correlations and make it distinguishable from biological correlations. So in this case, using multivariate normal distribution is computationally more efficient than multivariate t-distributions with a non-diagonal correlation matrix. 2) As the reviewer has pointed out that in the conditional analysis, Gaussian and conditional Gaussian distributions are statistically and computationally more convenient than conditional t-distributions. 3) When the complex trait of interest is binary, the test statistics for log odds ratios may be normally distributed instead of t-distributed by nature. Although it should be noted that we may still use moderated-t distributions to estimate the empirical null distribution to account for potential violations of normal assumptions in the real data, and if so, the empirical null distribution would not be N(0,1) but N(0, 𝜎2) with 𝜎2 being empirically estimated. Given most GWAS and omics QTL studies have sample sizes large enough for a good approximation, we choose to first estimate the moderate t-distributions and then approximate them with Gaussian.*

*Changes we made: To clarify this point, we added additional explanation on page 15 in the main text.*

5.	In the simulation, I think simulating test statistics directly from the assumed t- distributions is not realistic. Instead, I think SNP effect-sizes should be first simulated from a normal distribution for those causal SNPs, then simulate phenotypes and perform single variant tests to obtain test statistics.

*Our Response: We thank the reviewer for this suggestion. In the resubmission, we followed this suggestion and redid all of our simulations (Table 1-3). We simulated genotype data, and simulated phenotypes data from simulated genotypes with causal SNPs’ effect sizes from normal distributions, and then perform single variant tests to obtain summary test statistics as suggested. Moreover, in the conditional LD simulation (Table 3), to simulate genotype data with a realistic LD structure, we used the sim1000G package (PMID: 30646839) to simulate 1 million variants for 1000 subjects using chromosomes 8, 9 and 10 in the CEU 1,000 Genomes population. We divided the genotypes into regions of 1000 consecutive SNPs in order to form gene regions. And we simulated phenotypes/traits and calculated single variant test statistics as input for Primo analyses.*

*The improved simulations have helped demonstrate the robustness of the proposed Primo method, in particular in the case of conditional analysis where we simulated genotypes using real reference panel data from the 1000 genomes study. As shown in the results in Tables 1-3, Primo continued to demonstrate reasonable power and control of FDR.*

*Changes we made: We have updated the entire simulation section according to the reviewer’s suggestion. See page 6-9 of the main text. In particular, we rewrote the subsection “Evaluation of the performance of Primo conditional association analysis accounting for LD and sample correlations,” with realistic LD structures simulated from real genome data.*

6.	As shown in the simulation results in the supplementary text, the method is sensitive to the pre-specification of the π_k values, especially when over-specifying the π_k values.

*Our Response: We would like to clarify this point. First, the Primo algorithm only requires the pre-specification of 𝜃1’s (j=1,…,J studies), where 𝜃1  is the proportion of study-specific non-null statistics. We actually estimated the 𝜋𝑘’s ( 𝑘 = 1,2, … . , 𝐾 = 2𝐽) from the data. This is an advantage of Primo in comparison with existing and widely-used methods, coloc and moloc. When there are J=3 studies, the Primo algorithm requires only the pre-specification of 3 parameters while moloc would require the pre-specification of 23=8 parameters, some of which are less reported in the literature (or unknown).*

*Next, we respectfully disagree with the reviewer that the method is sensitive to the pre- specification of prior parameters. The purpose of showing 10-fold over-specification of 𝜃1’s may lead to biased estimation of 𝜋𝑘’s (Supplementary Table S1b) and slightly inflated FDR (Table 1- 3 in the main text) is more of advice for future users than acknowledging it as a limitation. 10-fold mis-specification of a key parameter is a substantial mis-specification in reality (such as 100 GWAS hits versus 1000 GWAS hits). For most GWAS and omics QTL studies, this is the type of mis-specification that one may avoid.*

*In the simulations presented in Table 1-3, we showed that when 𝜃1’s are correctly specified in a certain range or is substantially under-specified, then Primo would yield reasonable estimation of 𝜋𝑘’s and nice or even conservative control of FDR. When 𝜃1’s are under-specified, there might be some loss of power.  However, if 𝜃1’s       are substantially over-specified, the performance of Primo may be affected. So our suggestion to future users are, if 𝜃1 is not well known, we suggest under-specification rather than over-specification. In the GTEx analysis and related colocalization literature, often 𝜃1is specified in the range of 0.001 to 0.0001 for omics QTL studies and 10−5  to 10−6 for GWAS studies  (e.g.  PMID: 24830394, 26053495, 25743184, 19763151). The    GTEx consortium used 10−4 and 10−6 for eQTL and GWAS, respectively, in the colocalization analysis, also erring on the side of caution. Therefore, the over-specification of the prior parameters used in our simulations are indeed quite over-specified, increasing those parameters by 10-100 folds. And we observed only slightly inflated FDR when those parameters are specified way out of range. Thus, we argue that our method is not quite sensitive to the pre-specification of parameters   𝜃1’s and we suggest not to over-specify the parameter.*

*Moreover, we believe that our prior parameter specification and sensitivity to specification are an advantage over existing methods. The widely used method coloc and its extension moloc is known to be very sensitive to the specification of 𝜋𝑘, and many of the 𝜋𝑘’s are unknown. (e.g., what proportion of SNPs are GWAS SNPs and also eQTL but not meQTL?) There is limited prior knowledge  from  the  literature.  And  Primo  estimates  those  𝜋𝑘’s based  on  pre-specified 𝜃1’s (j=1,…,J studies) and the empirical data.*

*Changes we made: We see the confusions in the discussion of pre-specification of parameters, and now we emphasized this point in both simulations and Methods section and in the Discussion. We also changed the wording in the discussion. See pages 6, 7, 12-13.*

7.	In the application results, I am wondering what proportion of the identified SNPs with a particular association pattern actually have significant single variant test p-values for at least one trait? How we might be able to validate these findings?

*Our Response:*

*This is a good question. The answer to this question depends on the data. By jointly analyzing multiple sets of summary statistics from GWAS and omics QTL summary statistics, Primo  will  borrow  information  across  studies.  And  the  proportion  of  SNPs  in  a  particular association pattern that actually have significant single variant test p-values for at least one omics trait may also depend on the nature of the traits and the prevalence of the pattern in the genome.*

*Here we provide some examples on the single variant test p-values of GWAS SNPs analyzed by Primo: In the breast cancer susceptibility analysis (Application I), we identified all GWAS SNP- gene-methylation-protein quartets that were significant for “at least one QTL” in the analysis by Primo. For each quartet, we extracted the minimum p-value from the 4 QTL studies for “associations with at least one omics trait”. The largest of these minimum p-values was 0.00069 (corresponding to a t-statistic of 3.42) among all SNPs of interest. In other words, all quartets significant for “at least one QTL” had at least one QTL p-value less than or equal to 0.00069.*

*We also checked the test statistics for the Height-BMI analysis (Application II) identifying cases of pleiotropy while integrating eQTL summary statistics from adipose and skeletal muscle tissue. For each GWAS SNP-gene1-gene2 trio that was significantly associated with both complex traits in the Primo analysis, we extracted the minimum p-value of eQTLs and the second complex trait (for known height-associated SNPs, the secondary trait is BMI; and for known BMI- associated with SNPs, the secondary trait is height). And the largest of these minimum p-values was 0.0026 (corresponding to a t-statistics of 3.01).*

*That is, most if not all of our findings do show some evidences of association to at least one trait. Some of those associations might not survive multiple testing adjustment in simple analysis, and Primo analysis borrows information across studies and the results and the significance of the “identified SNPs” to at least one trait would depend on the data structure.*

*In Application II, we presented a replication study of the 32 pleiotropic SNPs being associated with both height and BMI, and we used GWAS summary statistics from UK Biobank as a replication study. At the P < 0.0008 cutoff (the Bonferroni threshold is calculated as 0.05/32/2 complex traits since there are two traits), 27 out of the 32 SNPs were associated with both traits in the UK Biobank, including 17 of the 18 SNPs that were also associated with gene expression. In Supplemental Materials, we also presented another set of analysis integrating GWAS summary statistics of Crohn's disease and ulcerative colitis with eQTL summary statistics from sigmoid colon and transverse colon tissues from GTEx. So many of our results can be replicated/validated in different studies.*

*See page 10-11 in the main text and Supplementary Materials.*

8.	I did not see any figure legends included in the manuscript, which make it very hard to understand all figures.

*Our Response:*

*We apologize for the difficulty since the legends were separated from the figures according to journal submission guideline. They are on page 22 of the main text, before Tables. In the resubmission, we included all four main Figures with Figure legends for reviewer’s convenience and attached it to the back of the response letter. Please excuse us for the redundancy.*

**Second round of review**

**Reviewer 1**

The authors have addressed all my comments

**Reviewer 2**

It is good to see the authors revised their simulation design and clarify their method descriptions. However, I still have the following major concerns.

*     The biggest concern I have is still about the justification of the distribution of t-statistics under alternative hypothesis. When the authors say they estimated f_0 and f_1 using genome-wide summary statistics per trait and their f_1(t_ij) is variant-specific with a variant-specific variance scale parameter, it seems that only the shared degrees of freedom of the scaled-t distribution under alternative distribution will be estimated using genome-wide summary statistics. The variant-specific variance scale parameter was derived directly from summary statistics. Then the f_1 distribution should be indexed with the scale parameter, such as f_1(t_ij; v_ij).

*     When using p-values, the authors estimate the shared alternative distribution f_1, a mixture of non-central chi-squared distribution, as a scaled chi-square distribution. Shouldn't the mixture of non-central chi-squared still have non-zero mean?

*     Another concern I have is that the authors seems treating all cis-eQTL or other cis- molecular QTLs generated with respect to different genes, epigenetic markers, or protein abundance as a group of summary statistics generated with respect to the same quantitative trait. This is not clear in the application studies. If this is the case, then the Primo will have conflict with analyzing genome-wide SNPs versus gene/epigenetic-marker/protein specific cis- QTL, and will have to consider different correlation structure among different molecular quantitative traits with respect to each gene/epigenetic-marker/protein.

*     It is also not clear in the application studies, did the authors only analyzed those SNPs within known GWAS loci and shared among those cis- molecular QTL, or actually applied Primo with genome-wide variants.

*     By the way, the replication studies the authors mentioned in the application section is only validating the GWAS signals in a different GWAS study, but not about the identified association pattern by Primo.

*     Figure 2 is confusing. As shown by Figure 3, the signal will be able to be picked up just by simply screen through each set of summary stat without requiring any more complicated statistical method like Primo.

**Authors Response**

**Point-by-point responses to the reviewers’ comments:**

Reviewer #2: It is good to see the authors revised their simulation design and clarify their method descriptions. However, I still have the following major concerns. 1. The biggest concern I have is still about the justification of the distribution of t-statistics under alternative hypothesis. When the authors say they estimated f_0 and f_1 using genome-wide summary statistics per trait and their f_1(t_ij) is variant-specific with a variant-specific variance scale parameter, it seems that only the shared degrees of freedom of the scaled-t distribution under alternative distribution will be estimated using genome-wide summary statistics. The variant-specific variance scale parameter was derived directly from summary statistics. Then the f_1 distribution should be indexed with the scale parameter, such as f_1(t_ij; v_ij).

*Our responses: Under the alternative, the f_1 distribution for each variant is a scaled t-distribution with moderated degree of freedom and a scaling parameter, 𝑣𝑖𝑗. On page 14 of the manuscript line 5-12, we provided the formula for the scaling parameter:*

*𝑣𝑖𝑗 = (1 + 𝑣0𝑗⁄𝑤𝑖𝑗) ^½^*

*where 𝑣0𝑗 is estimated using genome-wide summary statistics per trait and is estimated using the limma package in R [1], and 𝑤𝑖𝑗 is a variant-specific weight. Note that 𝑣0𝑗 is the key parameter capturing the deviation of alternative distribution from a standard t-distribution, and when 𝑣0𝑗 is close to zero, the alternative distribution is close to the null. In this work, we set the variant-specific 𝑤𝑖𝑗 as a function of minor allele frequency of SNP while users may potentially incorporate other information and prioritize certain variants via this weight. So in addition to the shared degrees of freedom, the scaling factor is also estimated using genome-wide summary statistics while also accounting for variant-specific information. The f_1 distribution could be indexed with the scale parameter, such as f_1(t_ij; v_ij), and here in our work, 𝑓_1_(𝑡_𝑖𝑗_; 𝑣_𝑖𝑗_) = 𝑣_𝑖𝑗_𝑔(𝑡_𝑖𝑗_), where 𝑔(𝑡_𝑖𝑗_) is the density function for a standard t-distribution with a moderated degree of freedom for each study/trait.*

2. When using p-values, the authors estimate the shared alternative distribution f_1, a mixture of non-central chi-squared distribution, as a scaled chi-square distribution. Shouldn't the mixture of non-central chi-squared still have non-zero mean?

*Our responses: In the p-value version of Primo, we first take –2log(P) as our test statistics, under the null, the log-transformed statistics are distributed as 𝜒^2^_2_ . Under the alternative, p-values can be modeled as Beta distributions Beta(a,1) [2]. Given the distribution of p-value under the null is uniform (with density function being 1), the log likelihood ratio test statistics has the same distribution as -2log(p) under the alternative, and is distributed as a non-central chi-square variable [3]. Based on the reference [4], the distribution of a sum of positively weighted (central and non-central) chi-square variables can be well approximated by a scaled central chi-square distribution 𝐴(*$X_{p}^{2}$*) ^𝑟^ , where A is a scaling parameter, p is the approximated degree of freedom, and r is the power, and A, p, and r are determined by the first three moments of the mixture distribution. In our analysis, we used a simpler form 𝐴(*$X_{p}^{2}$*) with power r=1 to approximate the mixture of chi-squares for two reasons: 1) since the estimation and approximation of alternative distribution f_1 is only an intermediate step in our algorithm for calculating probabilities and as long as f_1 is well-separated from f_0, simulations already show that the algorithm is robust to mis-specification of parameters to some extents; 2) our algorithm only requires the precise approximation of the tail distribution of f_1 not the entire distribution and the first two moments, mean and variance, are sufficient to capture the tail distribution of f_1. Additionally, the computation of the first two moments, mean and variance, of the empirical distribution is easily achievable. Extensive simulations have supported our approximation. The reviewer is right that the mixture of non-central chi-squared distributions has non-zero mean and the approximated distribution, a scaled central chi-square 𝐴*$X_{p}^{2}$ *is of mean 𝐴𝑝 and variance 2𝐴^2^𝑝, with A>0 and A≠1.*

3. Another concern I have is that the authors seems treating all cis-eQTL or other cis- molecular QTLs generated with respect to different genes, epigenetic markers, or protein abundance as a group of summary statistics generated with respect to the same quantitative trait. This is not clear in the application studies. If this is the case, then the Primo will have conflict with analyzing genome-wide SNPs versus gene/epigenetic-marker/protein specific cis- QTL, and will have to consider different correlation structure among different molecular quantitative traits with respect to each gene/epigeneticmarker/protein.

*Our responses: Taking our Application I for breast cancer GWAS SNPs as an example, for each GWAS SNP, we are interested in whether the GWAS SNP is associated with the gene expression, epigenetic variation and protein abundance for the same gene. And for each GWAS SNP, there are generally multiple cis-genes, and each cis-gene may have multiple cis CpG sites, and up to one total protein abundance measure (some gene may not have a protein quantification and the pQTL statistic is set to zero). The input data information in the Application I is illustrated below. Each row is for a GWAS SNP, with its association statistics to a cis-gene expression from normal GTEx, and from tumor TCGA tissue, association statistic to a cis CpG site matched to the same cis-gene (within 50kb of the SNP) and association statistic to the matched same gene’s protein abundance measure.*

*The table above is an illustration of data structure of one GWAS SNP rs6597981; the actual input data of Primo is all summary statistics from all SNPs examined to those quantitative traits matched to the corresponding gene in each row.*

*So although we only focus on 157 known breast cancer GWAS SNPs, for each GWAS SNP, there are generally many rows for different combinations of (gene, CpG, protein) of the same gene in the cisregion, with a mean of 226.1 rows/combinations and a median of 36 rows for each GWAS SNP.*

*As an example, if the test statistics from the first row is reported by Primo the association pattern of (0,1,1,1) after conditional association analysis, the conclusion is that the GWAS SNP rs6597981 is also an e-me-pQTL in tumor tissues but not in the normal GTEx tissue.*

*Note that although rows in the example table are correlated, the correlation is local, and the limma algorithm in estimating f_0 and f_1 allows for statistics being locally correlated (via moderated degrees of freedom estimated using a hierarchical model).*

*Different correlation structures among different molecular quantitative traits (correlations among columns) are indeed accounted for in Primo and in fact that is one advantage of Primo over existing methods (existing methods assume all studies being independent). There are two sources of correlations among traits, due to overlapping samples and due to biological co-occurrences. The three tumor omics QTL summary statistics are from TCGA with different subsets of overlapping samples. We first estimated the sample overlapping correlation under the null, and then estimated 𝜋_𝑘_ ’s to capture different correlation structures among different molecular quantitative traits, for example, eQTLs and pQTLs of the same gene are often co-occurring while meQTL and pQTLs are less likely to co-occur. Those parameters are all estimated from the data and that is an advantage of our algorithm in comparison with existing literature asking users to pre-specify those parameters.*

4. It is also not clear in the application studies, did the authors only analyzed those SNPs within known GWAS loci and shared among those cis- molecular QTL, or actually applied Primo with genome-wide variants.

*Our responses: In Application I, we analyzed only 157 known GWAS SNPs reaching genome-wide significance and can be mapped to both GTEx and TCGA data (with eQTL and omics QTL summary statistics available). See page 9 of main text. In Application II for pleiotropy analysis, we analyzed only GWAS SNPs reaching genome-wide significance for one of the complex traits. In the height and BMI pleiotropic analysis, there are 683 GWAS SNPs were presented in both sets of GWAS summary statistics reaching genome-wide significance and could also be mapped in GTEx. Of the 683 GWAS SNPs, 612 were associated with height, 78 with BMI and among them 7 were associated with both traits. Note that the 683 GWAS SNPs are the SNPs we analyzed. We did calculate key parameters such as 𝜋_𝑘_ ′𝑠 and estimated study-specific density functions, 𝑓_0𝑗_ ’s and 𝑓_1𝑗_ ’s using genome-wide variants. In comparison with existing methods requiring the user to specify 𝜋_𝑘_ ′𝑠, we demonstrated via simulations that the data-driving 𝜋𝑘 estimation using all summary statistics is an advantage of Primo. We rewrote part of the application studies to make this point clearer.*

5. By the way, the replication studies the authors mentioned in the application section is only validating the GWAS signals in a different GWAS study, but not about the identified association pattern by Primo.

*Our responses: The second application analyzed 683 GWAS SNPs that are reported to reach genomewide significance for being associated with either height or BMI (only 7 of which reached genome-wide significance for both), and the 32 SNPs we identified among the 683 are the ones found to be associated with both height and BMI while some (18) of them are also associated with cis-expression levels of a same gene in adipose and muscle tissues from GTEx. To be more precise, for the four studies, associations to height, BMI, cis-expression in adipose, and the same cis-gene expression in muscle, the target association pattern of interest is “at least associated with both height and BMI regardless of the association status to a cis-gene’s expression in adipose and muscle”. And the replication study is replicating the identified and target association pattern (associations with both height and BMI) using a different GWAS study.*

6. Figure 2 is confusing. As shown by Figure 3, the signal will be able to be picked up just by simply screen through each set of summary stat without requiring any more complicated statistical method like Primo.

*Our responses: Figure 2 shows a conceptual illustration of the motivation of Primo: some associations of a GWAS SNP to a cis-omics trait are due to being in LD with a real cis-omics QTL (the eQTL in the figure). Figure 3 is one of the findings of a GWAS SNP being also a multi-omics QTL from the real data analysis. Figure 3 represents the cleanest possible finding from real data while Supplemental Figure 2 provided the Locus-zoom plots for the 27 validated SNPs from the second data analysis for detecting pleiotropy effects and most of them show complicated patterns. When jointly analyzing multiple sets of summary statistics, it is non-trivial to determine the appropriate significance threshold for claiming associations and conditional associations in specific studies while accounting for study-study correlation, heterogeneity, LD and other factors. As more and more studies are jointly analyzed, it will be more likely to observe associations by random chance, and different studies have different power and different proportions of true associations; moreover, some associations of a GWAS SNP to an omics trait are due to being in LD with a true omics QTL; and some studies are correlated (e.g., two sets of eQTL summary statistics from GTEx, or three sets of tumor omics QTLs) – those complicated data structures motivate the Primo development.*

REFERENCE

1. Smyth, G.K., Linear models and empirical bayes methods for assessing differential expression in microarray experiments. Stat Appl Genet Mol Biol, 2004. 3: p. Article3.
2. Heard, N.A. and P. Rubin-Delanchy, Choosing between methods of combining p-values. Biometrika, 2018. 105(1): p. 239-246.
3. Banerjee, M., Likelihood ratio tests under local alternatives in regular semiparametric models. Statistica Sinica, 2005. 15(3): p. 635-644.
4. Solomon, H. and M.A. Stephens, Distribution of a Sum of Weighted Chi-Square Variables. Journal of the American Statistical Association, 1977. 72(360): p. 881-885.
